# Clinical impact of pharmacogenomics in pediatric care: insights extracted from clinical exome sequencing

**DOI:** 10.3389/fgene.2025.1574325

**Published:** 2025-05-29

**Authors:** Simran Maggo, Yachen Pan, Dejerianne Ostrow, Jenny Q. Nguyen, Jaclyn A. Biegel, Matthew A. Deardorff, Xiaowu Gai

**Affiliations:** ^1^ Bernard J. Dunn School of Pharmacy, Shenandoah University, Winchester, VA, United States; ^2^ Center for Personalized Medicine, Department of Pathology and Laboratory Medicine, Children’s Hospital Los Angeles, Los Angeles, CA, United States; ^3^ Personalized Care Program, Department of Pathology and Laboratory Medicine, Children’s Hospital Los Angeles, Los Angeles, CA, United States; ^4^ Department of Pathology and Laboratory Medicine, Keck School of Medicine, University of Southern California, Los Angeles, CA, United States; ^5^ Linda T. and John A. Mellowes Center for Genomic Sciences and Precision Medicine, Medical College of Wisconsin, Milwaukee, WI, United States; ^6^ Department of Pediatrics, Medical College of Wisconsin, Milwaukee, WI, United States

**Keywords:** pharmacogenomic (PGx) research, pharmacogenetic testing, bioinformatic analyses, admixed american pharmacogenomics, pharmacogenomics data

## Abstract

**Introduction:**

Pharmacogenomic (PGx) testing improves drug efficacy and reduces risk of toxicity for commonly prescribed medications, with most pharmacogenomic studies largely focused on individuals of European descent to date. The impact of pharmacogenomic testing in a racially diverse population is still emerging, especially for Admixed American patients.

**Methods:**

In this study, we assessed the frequency of actionable PGx variants by analyzing anonymized exome sequencing data of a racially diverse cohort of 1777 pediatric patients, collected for routine clinical genetic diagnosis at Children‘s Hospital Los Angeles (CHLA). Utilizing exome data, we used the Illumina DRAGEN germline pipeline v4.2, to determine the predicted phenotypes of 25 pharmacogenes including *HLA‐A* and *HLA‐B*, including CPIC Level A genes and genes recommended for PGx testing by the U.S. Food and Drug Administration. To assess cross-platform consistency, we compared our results to those generated by PyPGx, a pharmacogenomic genotyping tool developed by the same author as Stargazer. As the distribution of PGx alleles is ancestry specific, we estimated genetic ancestry bioinformatically using the Somalier tool.

**Results:**

Genetic ancestry analysis demonstrated that 62% of our cohort was Admixed American, followed by 23% European, 8% East Asian, 5% African American, and 2% South East Asian. Actionability analysis showed that: 1) 93% of all exome cases had at least one actionable PGx phenotype, 2) one in five cases (22%) had at least three actionable PGx phenotypes, and 3) CYP2B6 (54%) and CYP2D6 (33%) had the highest number of actionable phenotypes. Further analysis revealed notable differences, including higher rates of poor metabolizers for CYP2B6 and variations in CYP2D6 metabolizer statuses, in PGx phenotypes compared to previously collated frequencies in the PharmGKB database, especially within the Admixed American population.

**Discussion:**

In conclusion, our study reinforces the importance of PGx testing, underscores the diversity of PGx variation in ancestral backgrounds, and supports the clinical utility of preemptive PGx testing using exome or genome sequencing approaches.

## Introduction

The field of pharmacogenomics began to develop in the 1950s (Pirmohamed, 2011), and has advanced considerably with the advent of molecular biology techniques. Economic analyses have shown cost savings of approximately $4000 USD per patient per year by incorporating pharmacogenomic testing for drug selection for patients with depression (Maciel et al., 2018). However, the clinical implementation of pharmacogenomics in specialties such as cardiology, hematology and oncology has been underutilized, despite the finding that most patients have at least one actionable pharmacogenomic genotype (Mizuno et al., 2022; Klein et al., 2017; Luzum et al., 2021). Although challenges with interpreting DNA copy number or sequence variants previously limited the wide adoption of pharmacogenomic testing, this has largely been overcome by emerging consensus clinical guidelines (Abdullah-Koolmees et al., 2020), with strong contributions from several key organizations and databases, including Pharmacogenomics Knowledgebase (PharmGKB) (Hewett et al., 2002), the Clinical Pharmacogenetics Implementation Consortium (CPIC) (Relling et al., 2020), the Dutch Pharmacogenetics Working Group (DPWG) (Abdullah-Koolmees et al., 2020), the Pharmacogene Variation Consortium (PharmVar) (Gaedigk et al., 2018), and the Association for Molecular Pathology (AMP) Pharmacogenomics Working Group. With clear guidelines emerging, broader implementation of pharmacogenomic testing requires not only cost reduction but also the development of robust infrastructure, including clinical decision support (CDS) systems, data pipelines, and seamless integration of test results into electronic medical records (EMRs) (Klein et al., 2017; Luzum et al., 2021; Morris et al., 2024).

A potential way to reduce costs would be to interpret pharmacogenomic variants from previously generated data. Exome sequencing, which includes the targeted sequencing of all exons and intron boundaries in the genome has become an excellent first line tool for molecular diagnosis of suspected genetic disorders (Fung et al., 2020; Ross et al., 2020; Clark et al., 2018; Wang et al., 2013) as up to 80% of pathogenic disease variants are attributed to protein-coding variants. The ability to identify variations in many pharmacogenes, that is, genes responsible for drug metabolism, efficacy, and toxicity, increases the clinical utility of exomes to include pharmacogenomics (van der Lee et al., 2020; Ji and Shaaban, 2024). This is greatly facilitated by the development of multiple bioinformatics tools for genome-wide pharmacogenomic analyses, such as PharmCAT (Sangkuhl et al., 2020), Aldy (Hari et al., 2023), and PyPGx (Lee et al., 2022), which not only incorporate published clinical recommendations but also enable genotype calling, allele phasing, phenotype prediction, and automated interpretation to support clinical decision-making. Furthermore, the costs of exome and genome sequencing have been continuously dropping and in high throughput settings, and have reached less than $500 per sample. The ability to preemptively determine the genotypes of the most important pharmacogenes with exome and genome sequencing would obviate the need for different assays, reduce the cost of pharmacogenomic testing and greatly eliminate another hurdle in implementation.

Assessing the impact of pharmagogenomic testing on populations has additional barriers. To date, pharmacogenomic research has largely focused on populations of European descent, leading to a significant gap in knowledge regarding drug response in ancestral minorities, especially for Hispanic populations within the United States (Claudio-Campos et al., 2015). Hispanics are the largest minority group in the U.S., and as of the 2020 census, make up to 40% of the state of California’s population (Bureau USC, 2020). It is important to note that Hispanic is a term to represent a highly admixed population with ancestries primarily from Indigenous American, European, and African origins, contributing to unique genetic variability (Claudio-Campos et al., 2015; Maldonado et al., 2023). To eliminate disparities in providing pharmacogenomic testing, it is imperitive that robust data for all ancestral groups be gathered for appropriate variant interpretation.

In this study, we present results from a comprehensive pharmacogenomic analysis of 1777 pediatric clinical exome cases, demonstrating the expanded utility of exomes beyond traditional rare disease diagnostics. By leveraging bioinformatics tools such as DRAGEN and PyPGx, we were able to efficiently extract and interpret pharmacogenomic data, providing valuable insights into drug metabolism, efficacy, and toxicity for each patient. Through these efforts, we aim to contribute to the growing body of evidence supporting the clinical and economic benefits of pharmacogenomics and advocate for preemptive pharmacogenomic testing and its broader adoption in healthcare systems. This research is particularly important as it addresses the underrepresentation of Hispanic populations in pharmacogenomic studies, a significant gap in the current landscape of precision medicine. Furthermore, our results reinforce the necessity of incorporating pharmacogenomic data into EMRs and implementing CDS systems to facilitate real-time, evidence-based treatment recommendations.

## Methods

### Study cohort

The Center for Personalized Medicine (CPM) at Children’s Hospital Los Angeles (CHLA) has been providing clinical exome sequencing since 2016. Reanalysis of anonymized exome data sets was performed from 1,777 pediatric patients with suspected genetic disorders, along with the parents of a subset of these patients, for a total of 2,549 individuals. This research was approved by the CHLA-IRB (CHLA-17-00374-AM001). Genome data integrity and privacy was maintained as all data from samples processed at CPM are de-identified.

### Analytic workflow

**FIGURE 1:**
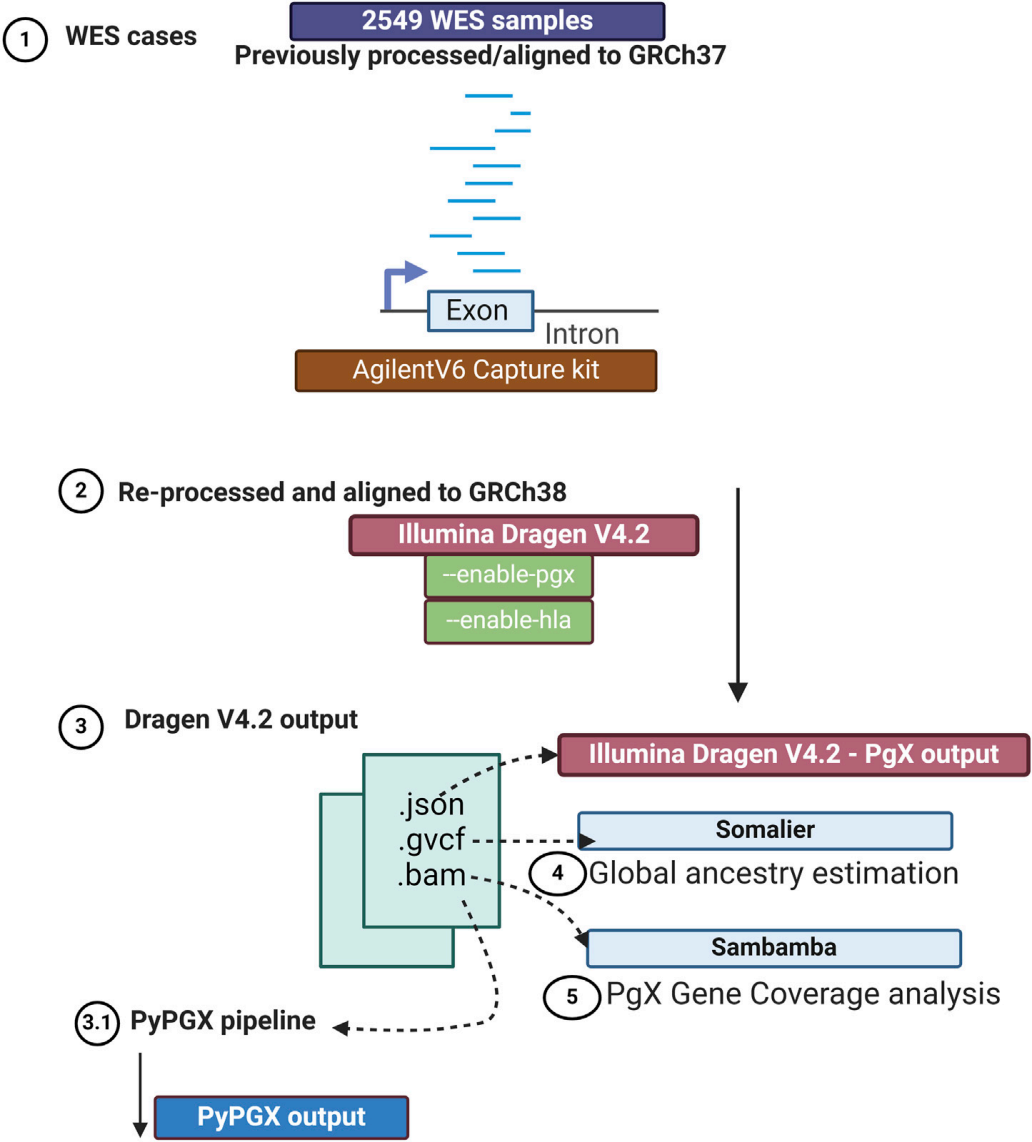
Bioinformatic workflow. As illustrated, 2549 previously sequenced exome cases were re-processed using DRAGEN V4.2. We activated specific flags (--enable pgx and--enable hla) to enable pharmacogenomic analysis. As shown in step 3, we utilized .gvcfs as it is recommended to use .gvcf for pharmacogenomic analysis. Furthermore, we used the results to estimate global genetic ancestry using the Somalier (Pedersen et al., 2020) tool. Coverage analysis of pharmacogenes was evaluated using the Sambamba (Tarasov et al., 2015) tool. Finally, we compared the pharmacogenomic output from DRAGEN V4.2 to that of PyPGx (Lee et al., 2022). Created with BioRender.com.

DNA was extracted from peripheral blood using a commercially available kit (Promega Maxwell RSC DNA Extraction Kit), or for reference samples, DNA was obtained from the Coriell Institute for Medical Research Biobank. Each exome sequencing library was generated from 500 ng of DNA sheared to target fragments of approximately 250bp in size, which were then captured using the Agilent SureSelect Human All Exon V6 (Agilent, CA, USA) plus a custom mitochondrial genome capture kit. (Falk et al., 2012). Paired-end 2 × 100 bp sequencing was performed using the Illumina NextSeq 500, HiSeq 4000, or Illumina NovaSeq X Plus sequencing system. For whole genome sequencing, 150 ng of DNA was sheared to an average size of approximately 350bp and libraries were created with the IDT xGen cfDNA and FFPE DNA Library Prep Kit. Libraries were sequenced at 2 × 150bp on the Illumina HiSeq 4000 or 2 × 165bp on the Illumina NovaSeq X Plus. As shown in Figure 1, Illumina DRAGEN version 4.2 with pharmacogenomic specific flags was utilized to realign these data sets to the GRCh38 reference genome. To assess exome sequencing coverage, a text file (Supplementary Data sheet S1) containing GRCh38 genomic locations of pharmacogenomic variants was used with the tool Sambamba (Tarasov et al., 2015) to assess the depth of coverage at pharmacogenomic variant positions. Genetic ancestry was estimated from .gvcf files using the bioinformatic tool Somalier (Pedersen et al., 2020).

### DRAGEN whole exome sequencing pipeline

Exome sequencing analysis was performed using the Illumina DRAGEN pipeline. Paired-end reads were aligned to the reference genome, and outputs were generated in sorted and indexed BAM format with duplicate reads marked. Variant calling was conducted to produce both VCF and GVCF files. Further details of the Dragen bioinformatic command line are provided in Supplementary Data sheet S2). The DRAGEN pharmacogenomic pipeline assesses the following genes *CYP2D6, CYP2B6, CACNA1S, CFTR, CYP2C19, CYP2C9, CYP3A5, CYP4F2, IFNL3, RYR1, NUDT15, SLCO1B1, TPMT, UGT1A1, VKORC1, DPYD, G6PD, MT-RNR1, BCHE, ABCG2, NAT2, F5, UGT2B17, HLA-A, HLA-B, HLA-C*. The majority of these genes are assigned CPIC Level A and/or are included in the FDA’s Table of Pharmacogenetic Associations. DRAGEN V4.2 examines .gvcf file (recommended) for known pharmacogenomic variants and presents results in JSON format. This was converted to a CSV file for ease of analysis using a custom Python script. An example JSON report (Supplementary Data sheet S2), CSV converted file (Supplementary Data sheet S3) and the Python script (Supplementary Data sheet S4) are provided in Supplementary Materials.

### PyPGx pipeline

The tool PyPGx (Lee et al., 2022) is a multifaceted pharmacogenomic Python package which has been developed by Lee, et al., who previously published the Stargazer pharmacogenomic pipelines (Lee et al., 2019a; Lee et al., 2019b). The current PyPGx tool assesses 87 pharmacogenes (Lee, 2024). We utilized the PyPGx pipeline to compare the pharmacogenomic output against DRAGEN V4.2. Briefly, we utilized *create-input-vcf* from PyPGx which uses BAM files to create a VCF file containing only those pharmacogenes assessed by the PyPGx tool (Lee, 2024). As per the recommended workflow, we also created necessary sample statistics and depth of coverage files necessary for analysis of pharmacogenes with copy number variation (CNV). We then utlized the GNU parallel tool (Tange, 2018) to run the PyPGx pipeline using the *run-ngs-pipeline* for each pharmacogene for all samples. The PyPGx tool returns results per gene in a TSV format with genotype, phenotype, haplotypes, alternative phase data and CNV data if available. Detailed documentation, example outputs and a step-by-step tutorial of the PyPGx tool are available online (Lee, 2024).

### Data analysis

Data manipulation, analysis and graphing were performed in R studio using R version 4.3.1. Pharmacogenomic phenotypes reported in this study are bioinformatically predicted based on available sequencing data and do not include functional assays or direct drug response measurements. Predicted phenotype classifications by both DRAGEN and PyPGx utilize established pharmacogenomic variant functionalities, as defined by standardized guidelines. Both tools report the genotype; however, when a variant’s functionality is not characterized or unknown, the tools classify the phenotype as indeterminate.

## Results

### Pharmacogenomic and coverage analysis

**FIGURE 2:**
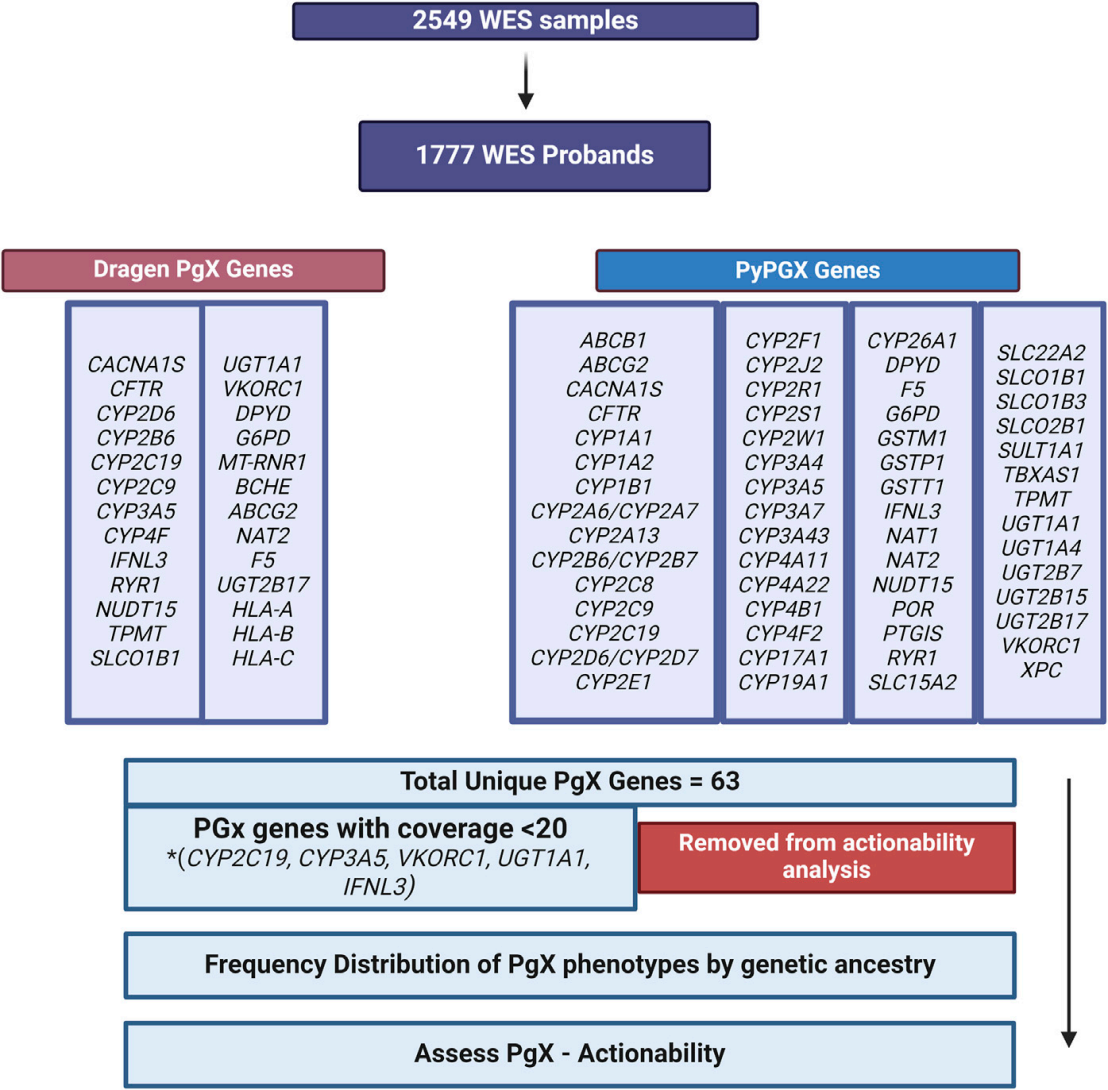
Workflow illustrating pharmacogenomic analysis. 1777 probands were assessed for pharmacogenes using DRAGEN V4.2 and PyPGx. * The exome capture kit used did not provide sufficient coverage for some important genetic variants crucial to determining proper haplotypes and therefore phenotypes (e.g., CYP2C19*17, CYP3A5*3). As a result five genes were excluded from our pharmacogenomic actionability analysis due to risk of incorrect phenotype assignment. Created with BioRender.com

**FIGURE 3:**
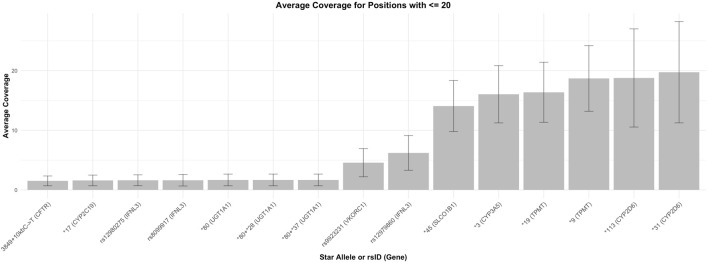
Inadequate (≤20X) coverage of important pharmacogenetic variants across all exome cases. Firgure shows average coverage (COV) in shaded bars and error bars show standard deviation (SD).

**FIGURE 4:**
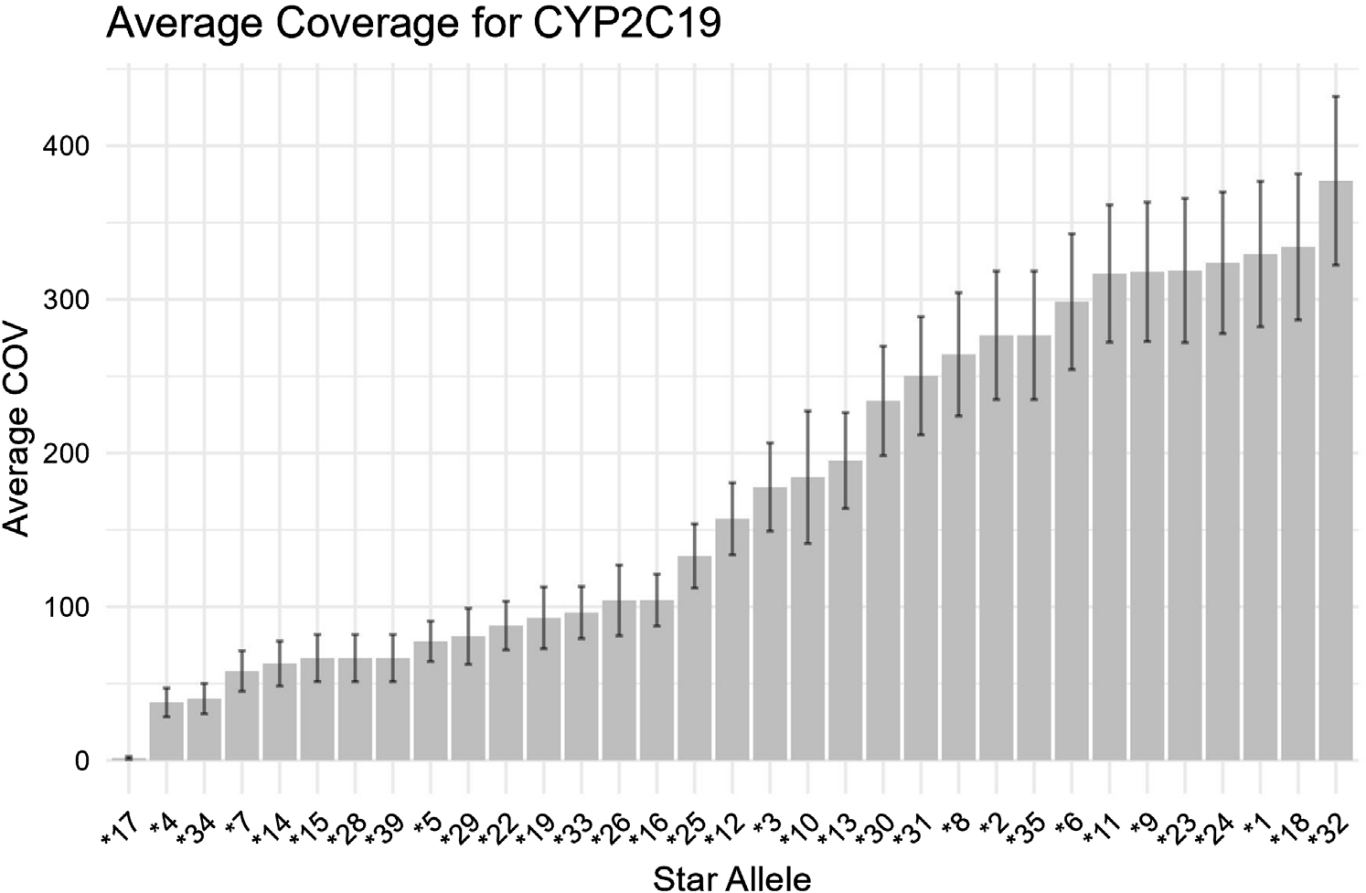
Average coverage of the CYP2C19 gene for all exome samples. As shown, coverage for the critical CYP2C19*17 SNP was close to zero. Firgure shows average coverage (COV) in shaded bars and error bars show standard deviation (SD).

A primary objective of this analysis was to assess pharmacogenomic specific information from exome data using the DRAGEN V4.2 pipeline. Secondly, we compared the pharmacogenomic output, specifically predicted pharmacogenomic genotype and phenotype classification of DRAGEN V4.2 against the pharmacogenomic pipeline PyPGx. For pharmacogenomic analysis and pipeline comparison we excluded parental samples (n = 772) from duos or trios and only analyzed probands (n = 1777; Figure 2). The average sequencing coverage for the exome and selected pharmacogenes was on average 162X, ensuring sufficient depth for reliable variant calling in these genes. However, we excluded five genes from analysis due to poor (<20X) or variable coverage (see Figure 3) in core pharmacogenomic variants. These variants (CYP1C19*17 – Figure 4, UGT1A1*80, CYP3A5*3, INFL3 variants–rs12980275, rs8099917, rs12979860, VKORC1 variant- rs9923231 (figures showing coverage for all genes assessed are shown in Supplementary Data sheet S5) are important haplotype-defining variants that occur in either promoter or intronic regions, and are not covered adequately by the exome capture kit utilized. Surprisingly, rs776746 which defines CYP3A5*3 is located one base prior to the start of exon four of *CYP3A5* had an average coverage of only 15X (Supplementary Data sheet S6). On examining the Agilent.bed file, we found that the CYP3A5*3 SNP (chr7:99672916) was not covered by the capture probeset, with the closest targeted regions flanking but not overlapping this position (chr7:99671711–99671921, chr7:99672662–99672783, and chr7:99674455–99674752). This explains the failure to detect CYP3A5*3 in the exome data.

The average coverage for each pharmacogene including variants used to determine haplotype and therefore phenotype are presented in Supplementary Data sheet S5. As a result, we limited our analysis to the following genes assessed by the DRAGEN V4.2 pipeline; *CYP2D6, CYP2B6, CACNA1S, CFTR, CYP2C9, CYP3A5, CYP4F2, RYR1, NUDT15, SLCO1B1, TPMT, DPYD, G6PD, MT-RNR1, BCHE, ABCG2, NAT2, F5, HLA-A, HLA-B, HLA-C*.

### Coriell sample assessment and comparison against genome sequencing

To evaluate the accuracy of the DRAGEN V4.2 and PyPGx pharmacogenomic pipelines, we assessed 10 DNA samples from the Coriell Institute of Medical Research with previously published pharmacogenomic data (Lee et al., 2022; Pratt et al., 2016) and conducted both exome and genome sequencing as described. The selected samples included those with challenging haplotypes, such as CYP2B6 *1/*6, CYP2D6 *1/*5 and CYP2D6 *2/2+2, which are difficult to assess due to structural variations and the close proximity of homologous pseudogenes that affect the accuracy of *CYP2D6* and *CYP2B6* genotyping (Desta et al., 2021; Turner et al., 2023; Chen et al., 2021; Tremmel et al., 2023). DRAGEN V4.2 utilizes the Cyrius tool, which accurately calls CNV from genome sequence data for *CYP2D6* and *CYP2B6*, while PyPGx utilizes a machine learning-based approach to estimate copy number and detect SVs and has demonstrated accuracy in calling CNVs from various NGS data sources (Lee et al., 2022; Sherman et al., 2024). Notably, two of the Coriell samples (NA17280, NA02016) also had existing consensus HLA typing data (Bettinotti et al., 2018). This study aimed to evaluate the pharmacogenomic accuracy of these pipelines, including their ability to accurately call CNVs in challenging genotypes, by leveraging well-characterized reference samples with known CNVs and previously established consensus genotypes (Pratt et al., 2016; Scott et al., 2021; Calendo et al., 2024; Gharani et al., 2024).

Consensus genotypes from the Genetic Testing Reference Materials Coordination Program (GET-RM) were available for seven of the ten samples (See Supplementary Table S1). Assessing the accuracy of genotyping across selected genes (*CYP2B6, CYP2C19, CYP2C9, CYP2D6, CYP3A5, CYP4F2, DPYD,* and *SLCO1B1*), PyPGx outperformed DRAGEN overall, particularly for genome data, where PyPGx achieved a 94.6% accuracy rate compared to DRAGEN’s 87.5%. For exome data, PyPGx also showed better performance with an 85.7% accuracy rate compared to DRAGEN’s 75.0%. Both methods performed perfectly for genes such as *CYP2C9, CYP4F2*, and *TPMT*, but PyPGx consistently outperformed DRAGEN for more complex genes, especially with genomes where PyPGx had 100% correct calls for *CYP2D6* and other challenging genes. As previously noted, while both callers successfully called *CYP3A5* accurately, the overall average read-depth across the *3 SNP was too low (<20×) to include this in our overall analysis. As already mentioned, the Agilent SureSelect Human All Exon probeset does not cover this region which explains the insufficient read depth in exome data, whereas whole-genome sequencing (WGS) is expected to provide full coverage at this locus. Furthermore, as noted for *CYP2C19* in NA12813 and NA19908, these samples were heterozygous and homozygous for the *17 SNP respectively, and were incorrectly genotyped using exomes, which significantly altered the final phenotype. This was also noteworthy for the *CYP2D6* and *CYP2B6* genes which were incorrectly assigned duplications, by both callers for exomes only. It is important to note that DRAGEN, which uses the Cyrius tool for *CYP2D6* and *CYP2B6* genotyping, advises using genomes for accurate genotyping. In some instances, the DRAGEN tool provided multiple potential genotype assignments likely due to limitations in allele phasing. For example, DRAGEN occasionally reported two possible *CYP2B6* genotype combinations (*1/*6 or *4/*9), whereas PyPGx assigned a single genotype (*1/*6) based on population frequency data. Further, for the *DPYD* gene, DRAGEN was unable to accurately call *9/*9, and for one Coriell sample, unable to genotype *1/*9. However, PyPGx was able to call these genotypes accurately. For *SLCO1B1*, DRAGEN also identified one sample with two potential genotype assignments (*4/*44 or *43/*1). These observations reflect the distinct approaches each software employs when allele phasing ambiguity is encountered; DRAGEN reports all possible genotype combinations, highlighting uncertainty, whereas PyPGx resolves ambiguities by selecting the most commonly reported genotype from population data. Therefore, several of these discrepancies are not strictly errors, rather limitations of phasing (i.e., is the allele in *cis* or *trans*) by the Dragen caller. PyPGx reports the most frequent diplotype when phasing is a concern using a method referred to as “Phase-by-extension algorithm“ as mentioned in the tools online documentation (Lee, 2024). Furthermore, “diplotyping” errors are likely due to the complexity of variants present in each star allele, especially for complex and/or pharmacogenes with extensive genetic variation (Gaedigk et al., 2018).

### Genetic ancestry of exome cases

**FIGURE 5:**
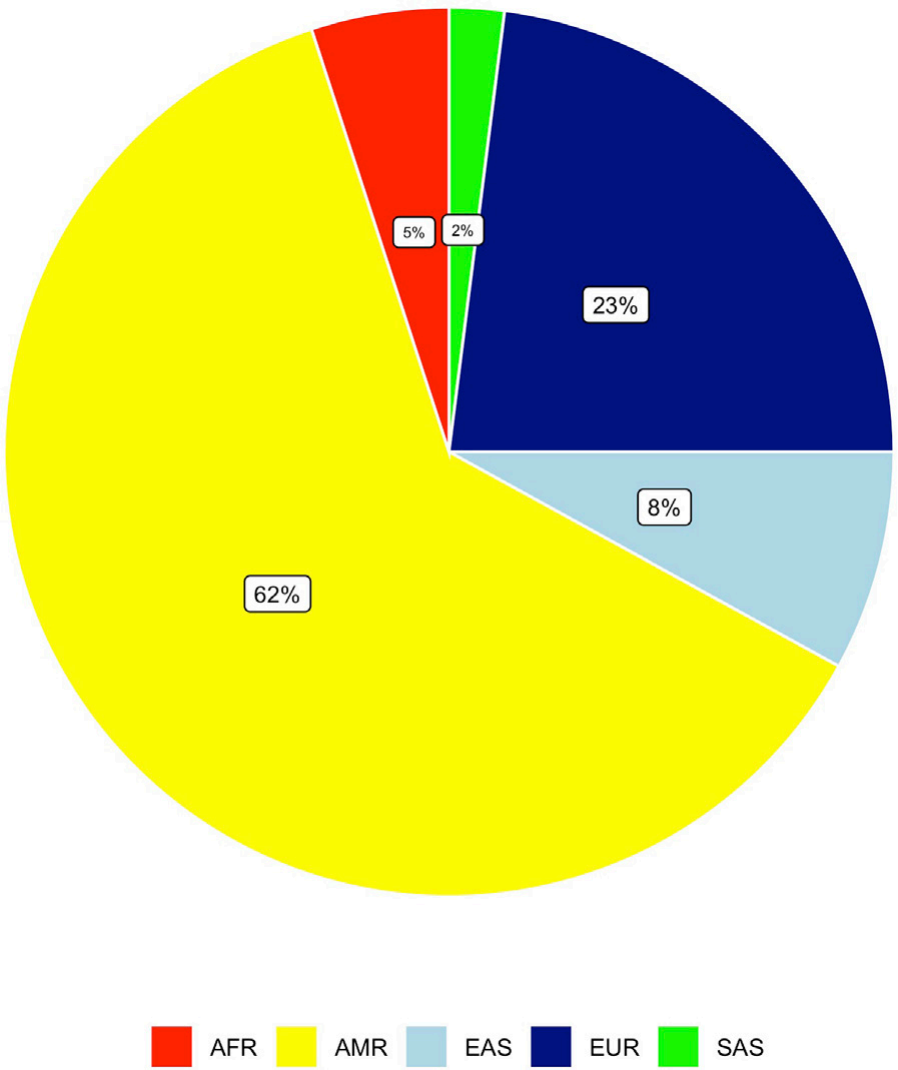
Genetic ancestry distribution of exome probands. Figure shows the 1000 Genomes super population categories identified in our cohort.

Genetic ancestry analysis of 1,777 probands indicated that 62% of all exome cases analyzed were predicted to be of AMR genetic ancestry (Figure 5), consistent with the geographical location of CHLA. Owing to the mixed ancestry makeup, we conducted phenotype analysis by predicted-ancestral proportions.

### Phenotype analysis

**FIGURE 6:**
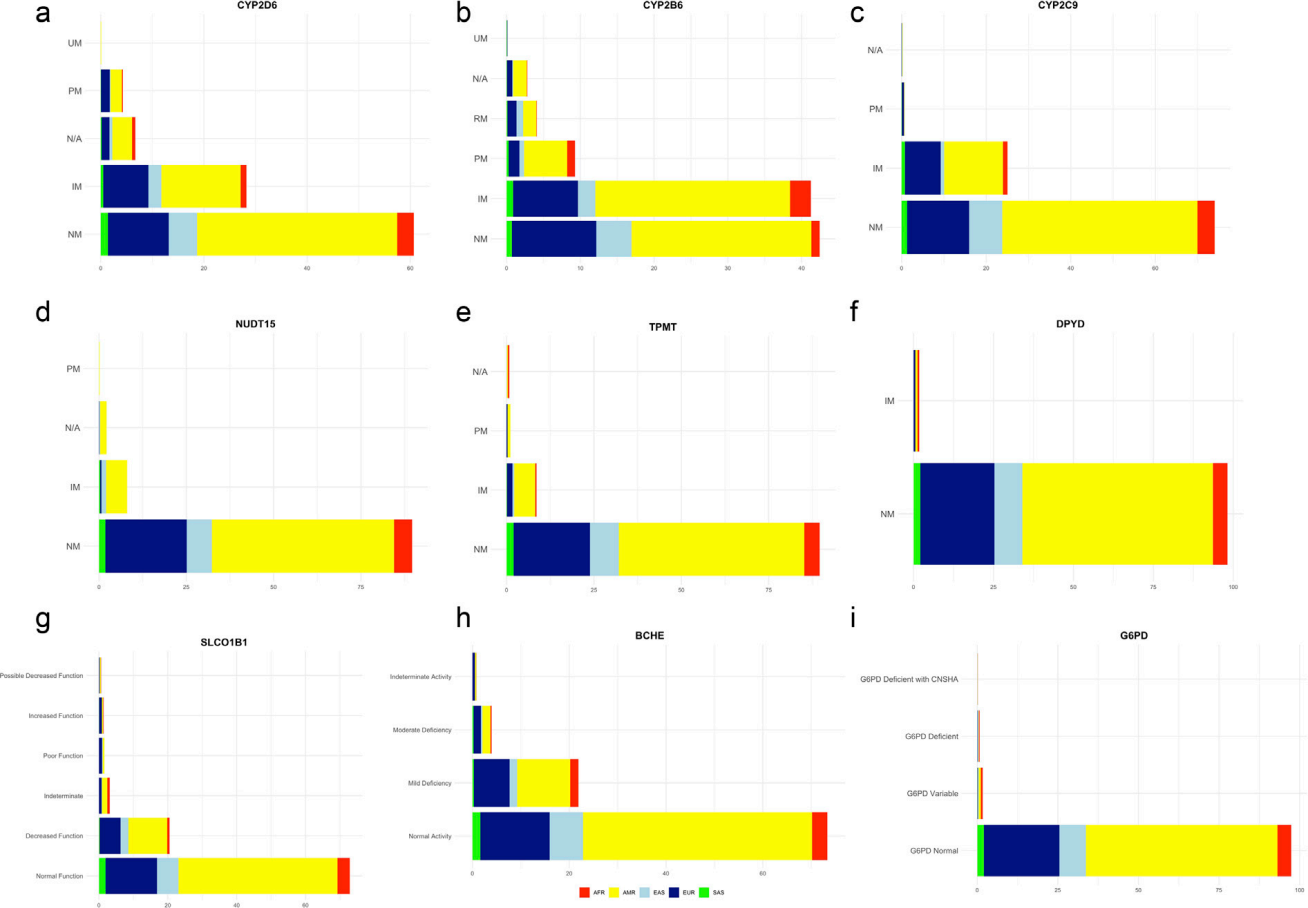
**(a–i)** Stacked bar plots showing the distribution of predicted pharmacogenetically relevant enzyme activity phenotypes by genetic ancestry (n = 1777). The y-axis represents phenotype categories, and the x-axis indicates the percentage of individuals within the total population. Phenotype abbreviations: NM, Normal Metabolizer; RM, Rapid Metabolizer; UM, Ultrarapid Metabolizer; IM, Intermediate Metabolizer; PM, Poor Metabolizer; N/A, Not available or Indeterminate phenotype classification.

Phenotypes as defined by DRAGEN V4.2 are shown in Figure 6a-i for the pharmacogenes *CYP2D6, CYP2B6, CYP2C9, NUDT15, TPMT, DPYD and SLCO1B1* (All gene graphs are presented in Supplementary Data sheet S7). Phenotypes for *G6PD* and *BCHE* were not automatically assigned by DRAGEN, only genotypes were provided. Existing guidelines from CPIC and Zhu et al. (2020) were utilized to ascertain phenotypes for *G6PD* and *BCHE* respectively (Gammal et al., 2023) (Zhu et al., 2020). Extended analysis for star alleles (Supplementary Table S2) and phenotypes (Supplementary Table S3) was also conducted and this data is available for each gene by predicted ancestry in Supplementary Materials.

### HLA Analysis

As part of the pharmacogenetic analysis pipeline, DRAGEN V4.2 reports *HLA-A*, *HLA-B* and *HLA-C* genotypes. The following *HLA* genotype-drug combinations were identified from CPIC (CPIC) and the literature (Manson et al., 2021) and defined as *HLA*-actionable. For the purpose of this analysis; B*57:01 = abacavir, B*57:01 = flucloxacillin, B*58:01 = allopurinol, A*31:01 = carbamazepine, B*15:11 = carbamazepine, B*15:02 = carbamazepine, B*15:02 = oxcarbazepine, B*15:02 = lamotrigine, B*15:02 = phenytoin, and B*15:02 = fosphenytoin. HLA-drug actionability analysis revealed differences based on genetic ancestry (Supplementary Data sheet S8). For example, phenytoin and allopurinol are less pertinent in AMR patients comparing to East Asian (EAS) patients, while the opposite is true for lamotrigine and flucloxacillin. The results from this analysis showed that approximately 8% of the population analyzed had at least one of the *HLA-*genotypes defined as actionable. As our exome data is anonymized we were unable to link to prescribed medication, however, extrapolating the data, we predicted the percentage of medication likely to be impacted, based on the prevalence of the selected *HLA-A* and *HLA-B* alleles in our population (Supplementary Data sheet S8).

### Actionability analysis

For this analysis we defined specific predicted pharmacogenomic phenotypes as “actionable”, and assigned them a value of “1”. A full list of possible phenotypes is as follows; Normal Function = 0, Decreased Function = 1, Poor Function = 1, Uncertain Susceptibility = 0, NA (No Call) = 0, Intermediate Metabolizer = 1, Normal Metabolizer = 0, Poor Metabolizer = 1, Rapid Metabolizer = 1, Normal = 0, Possible Decreased Function = 0, Increased Function = 1, Favorable Response = 0, Ultra Rapid Metabolizer = 1, Possible Intermediate Metabolizer = 0, Malignant Hyperthermia Susceptibility = 1, Normal Risk = 0, increased risk of aminoglycoside-induced hearing loss = 1, uncertain risk of aminoglycoside-induced hearing loss = 0, normal risk of aminoglycoside-induced hearing loss = 0, moderate deficiency–*BCHE* = 1, mild deficiency or normal activity–*BCHE* = 0*,* G6PD variable or deficient or G6PD deficient with CNSHA–*G6PD* = 1, G6PD Normal = 0. Further, the following *HLA* variants were assigned a value of “1′ B*57:01, B*58:01, A*31:01, B*15:11, B*15:02. The following genes were included in the actionability analysis; *ABCG2, CACNA1S, CFTR, CYP2B6, CYP2C9, CYP2D6, CYP4F2, DPYD, G6PD, BCHE, NAT2, NUDT15, RYR1, SLCO1B1, TPMT, MT-RNR1, HLA-A, HLA-B*.

**FIGURE 7:**
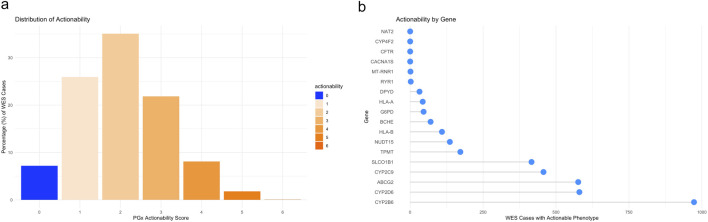
**(a)** shows the average % of actionable phenotypes per exome case, indicating that the majority – 92.8% of all exome cases had at least one actionable PGx phenotype. **(b)** shows the distribution of genes with actionable phenotypes. As shown CYP2B6 had the largest number of actionlable PGx phenotypes.

Using this criteria we observed that 92.8% (Figure 7a) of all exome cases assessed had at least one actionable pharmacogenomic variant. Furthermore, one in five individuals (21.8%) had at least three actionable pharmacogenomic variants. The top five pharmacogenes with actionable phenotypes (Figure 7b) were *CYP2B6, CYP2D6, ABCG2, CYP2C9 and SLCO1B1.* Actionability scores by gene are likely to reflect the presence of well-characterized deleterious or unregulated star alleles. Notably*, CYP2B6* exhibited higher actionability than *CYP2D6*, which is somewhat unexpected given the clinical significance of *CYP2D6* phenotypes. However, as previously noted, Dragen’s gene-specific caller is not suitable for identifying *CYP2B6/CYP2D6* genotypes from WES data, which may contribute to the presented data.

### Phenotype frequency and comparison with PharmGKB

An analysis of frequencies of the top five pharmacogenes with actionable phenotypes; *CYP2B6, CYP2D6, ABCG2, CYP2C9,* and *SLCO1B1* showed notable differences in metabolizer status (Table 1). This comparative analysis of exome data with existing data from PharmGKB across various ancestries shows ancestry-specific genetic variability in drug metabolism and immune response, particularly among Hispanic individuals (AMR genetic ancestry). For example, *CYP2B6* - exome data showed a higher prevalence of poor metabolizers (13.2%) compared to PharmGKB (9.5%) in the AMR group. For *CYP2D6*, the AMR cohort had notable fewer intermediate metabolizers (25.5% exome vs. 34.2% PharmGKB) and fewer ultrarapid metabolizers (exome-0.2% compared with PGKB-3.8%). Data for AMR was not available from PharmGKB for *SLCO1B1*, however, exome data for *SLCO1B1* showed notable differences for individuals predicted to be of African (AFR) ancestry. *ABCG2* phenotype frequencies were largely consistent between exome and PharmGKB (5.5% vs. 5%). For *CYP2C9*, the exome cohort showed a slightly higher frequency of normal metabolizers (76.6%) and a reduced proportion of poor metabolizers (0.2% vs. 1%). HLA allele analysis showed comparable frequencies of *HLA-A* and *HLA-B* alleles. It is important to note that this analysis of 1,777 exomes comprises predominantly (60%) individuals predicted to be of AMR genetic ancestry. Finally, we have included phenotype data from a recent comprehensive analysis of the 1000 genomes data using the PyPGx tool (Sherman et al., 2024). Given the inherent differences between the datasets we did not conduct statistical analyses.

**TABLE 1:** Phenotype frequency (%) comparison of the Whole Exome Sequencing cohort (CES) against data published on the PharmGKB (PGKB) website and a recent publication (LIT) (Tremmel et al., 2023). For population comparisons, AMR (Admixed American) from CES was compared against LAT (Latino) from PGKB due to differences in biogeographical classifications. Blank values for, PGKB, or LIT indicate values not available at the time of publication.

CYP2B6	AFR			AMR			EAS			EUR			SAS		
	CES	PGKB	LIT	CES	PGKB	LIT	CES	PGKB	LIT	CES	PGKB	LIT	CES	PGKB	LIT
Normal Metabolizer	22	33.4	25.4	34.2	33.6	35.4	54.5	48.6	51.2	48.2	43	51.2	34.2	42.2	30.9
Intermediate Metabolizer	54.9	46.6	47.7	42.1	42.2	43.5	26.9	32.6	33.1	37.1	38	35.1	42.1	36.5	45.4
Poor Metabolizer	20.9	15.7	21.5	13.2	9.5	16.4	7.1	4.4	5.2	6.2	7.4	5.8	13.2	6	15.1
Rapid Metabolizer	1.1	1.2	2.7	5.3	12.3	1.4	10.3	11.7	9.3	5.2	5.4	4.8	5.3	12.9	6.5
Ultrarapid Metabolizer	0	0	0	2.6	1.1	0	0	0.7	0.4	0.2	0.2	0.4	2.6	1	0.0
Indeterminate	1.1	3.1	2.2	2.6	1.4	3.1	1.3	2	0.8	3.1	6.1	2.8	2.6	1.5	2.0

CYP2D6	AFR			AMR			EAS			EUR			SAS		
	CES	PGKB	LIT	CES	PGKB	LIT	CES	PGKB	LIT	CES	PGKB	LIT	CES	PGKB	LIT
Normal Metabolizer (1.25 ≤ x ≤ 2.25)	62.6	31.4	50.1	64.3	54.6	65.1	62.8	52.1	52.4	49.6	48.2	48.4	65.8	57.8	59.7
Intermediate Metabolizer (0 < x < 1.25)	22	59.7	36.2	25.5	34.2	24.2	28.8	40	44.6	36.8	39.3	38.4	23.7	28.4	25.4
Poor Metabolizer (0)	3.3	2.3	2.1	3.6	3.1	3.5	1.9	0.8	0.0	7.1	6.5	6.2	2.6	2.4	2.9
Ultrarapid Metabolizer (>2.25)	0	2.6	5.1	0.2	3.8	3.2	0	0.8	1.0	0	2.3	3.0	0	1.5	1.2
Indeterminate	12.1		6.5	6.3		4.0	6.4		2.0	6.4		4.0	7.9		10.8

ABCG2	AFR			AMR			EAS			EUR			SAS		
	CES	PGKB	LIT	CES	PGKB	LIT	CES	PGKB	LIT	CES	PGKB	LIT	CES	PGKB	LIT
Normal Function	92.3	93.1	98.2	61.8	60.2	79.3	50	48	72.0	81.7	80.4	89.0	86.8	82.2	81.4
Decreased Function	7.7	6.7	1.8	32.7	34.8	19.9	42.9	42.6	23.8	17.1	18.6	10.2	10.5	16.9	18.2
Poor Function	0	0.1	0.0	5.5	5	0.9	7.1	9.4	4.2	1.2	1.1	0.8	2.6	0.9	0.4

CYP2C9	AFR			AMR			EAS			EUR			SAS		
	CES	PGKB	LIT	CES	PGKB	LIT	CES	PGKB	LIT	CES	PGKB	LIT	CES	PGKB	LIT
Normal Metabolizer	79.1	75.9	78.5	76.6	74.3	72.9	89.1	83.8	91.7	62	62.8	63.1	60.5	59.6	68.7
Intermediate Metabolizer	20.9	23.6	21.2	23.1	24.6	26.2	9.6	15.2	8.1	35.6	34.5	34.7	36.8	36.2	27.2
Poor Metabolizer	0	0.5	0.3	0.2	1	0.9	0	0.6	0.2	2.1	2.6	2.2	2.6	3.8	2.7
Indeterminate	0	0	0.0	0.2	0.1	0.0	1.3	0.5	0.0	0.2	0.1	0.0	0	0.4	1.4

SLCO1B1	AFR			AMR			EAS			EUR			SAS		
	CES	PGKB	LIT	CES	PGKB	LIT	CES	PGKB	LIT	CES	PGKB	LIT	CES	PGKB	LIT
Normal Function	69.2	98	51.7	76.7		50.4	70.5	75.7	72.2	63.2	65.6	39.2	89.5	86.5	72.6
Decreased Function	13.2	2	1.4	18.7		19.9	26.3	21.8	19.8	25.7	28.3	20.1	7.9	13	7.0
Possible Decreased Function	1.1	0	5.1	0.6		0.0	0.6	0.1	0.0	0.7	0	0.0	0	0	0.0
Poor Function	0	0	0.2	0.8		1.7	1.9	1.6	2.0	3.8	2.9	2.0	0	0.5	0.4
Increased Function	2.2	0		0.7			0	0		3.3	3		2.6	0	
Indeterminate	14.3	0	33.3	2.6		13.8	0.6	0.8	5.4	3.3	0.1	18.7	0	0.1	8.4

HLA - Allele	AFR			AMR			EAS			EUR			SAS		
	CES	PGKB		CES	PGKB		CES	PGKB		CES	PGKB		CES	PGKB	
HLA-A*31:01	0	0.99		1.63	4.52		0.96	3.45		0.59	2.64		0	3.3	
HLA-B*15:02	0.55	0.1		0	0.03		6.73	4.56		0	0.01		0	2.59	
HLA-B*57:01	0	0.61		0.56	1.39		0.32	0.98		3.09	3.6		5.26	6.81	
HLA-B*58:01	2.75	3.82		1.07	1.35		3.85	6.04		0.48	0.77		3.95	4.19	

## Discussion

This study provides a comprehensive pharmacogenomic analysis using data from exomes of a large cohort of 1,777 probands. We utilized two bioinformatics tools, DRAGEN V4.2 and PyPGx to call genotypes and assign phenotypes. While our findings suggest that PyPGx may be preferable for WES-based pharmacogenomic analyses, further validation in independent datasets is needed to assess tool performance across different sequencing platforms and variant types. These findings highlight the importance of selecting bioinformatics tools that are appropriately designed for the sequencing data type used in pharmacogenomic research and clinical implementation. The comparison of exomes to genomes highlights the limitations of exomes in capturing key pharmacogenomic variants. Exomes exhibited significant coverage gaps, particularly in non-coding regions crucial for correct phenotype assignment. In addition, for the pharmacogene *CYP2D6* which has a wide array of structural variation, duplications were often misassigned in exome data; however deletions such as *5 were accurately identified.

This study also reinforces the variation in pharmacogenetic phenotypes by genetic ancestry, with 62% of all exome cases analyzed predicted to be of AMR ancestry, adding to the pharmacogenomic knowledgebase for this population, and specifically for CHLA and the wider Los Angeles area. The analysis of *HLA* genotypes showed that approximately 8% of exome cases carry actionable *HLA* variants, especially for drugs used in neurology such as phenytoin and carbamazepine. A critical finding of our analysis is that 92.8% of exome cases had at least one actionable pharmacogenomic phenotype, with 21.8% having three or more. This study is unique as it utilizes exome sequencing data from a predominantly Hispanic pediatric cohort at CHLA, revealing that 62% of the population is predicted to be AMR. Such findings underscore the need for more inclusive pharmacogenomic research to improve precision medicine approaches in populations with complex ancestries that significantly influence pharmacogenetic variability (Claudio-Campos et al., 2015; de Andres et al., 2017; Popejoy, 2019; Magavern et al., 2022).

A recent study evaluating pharmacogenetics of 5,001 clinical exome cases reported that 95% of individuals carried one actionable phenotype (Lanillos et al., 2022). While our study reports ∼93%, it is important to note we excluded five important pharmacogenes due to limitations of the exome capture kit currently utilized. It would be safe to assume that inclusion of the pharmacogenes *UGT1A1, CYP3A5, CYP2C19, VKORC1* and *IFNL3* would have likely increased our percentage actionability. Further, recent analyses of large biobanks have shown similar pharmacogenetic actionability findings. A pharmacogenomic analysis of the UK-Biobank showed that 100% of all individuals (N = 200,044) had a pharmacogenetic variant of interest (Li et al., 2023). An analysis of 98,950 individuals from the All of US study, has reported that 100% of study participants carried a pharmacogenetic variant and 99% had a phenotype with prescribing recommendations (Haddad et al., 2024). Finally, an analysis of the Penn Medicine BioBank reported that 100% of individuals with genotype information (∼43,000) had at least one non-reference pharmacogenomic allele, and 98.9% had one or more actionable pharmacogenomic phenotypes (Verma et al., 2022). In addition, Verma *et al* (2022) reported that over 14% of patients were prescribed a medication for which they possess an actionable allele during the 8-year study period (Verma et al., 2022). While our analysis was limited to pharmacogenomic information, we can extrapolate medical specialties (neurology, pain, psychiatry, oncology, cardiology and others) which would benefit from integrating pharmacogenomic information into routine patient care. As shown in Figure 7b, the top five genes with actionable phenotypes were *CYP2B6, CYP2D6, ABCG2, CYP2C9 and SLCO1B1.* These five genes are involved in multiple drug-gene pathways (Supplementary Data sheet S9), and several of them have existing prescribing recommendations in the form of CPIC guidelines.

To replicate the requirements of clinical genetic sequencing, we conducted a depth of coverage analysis for each pharmacogenetically actionable allele and collected genotype information across all relevant loci. This step helped us to prevent inaccurate genotyping caused by low coverage. Among the pharmacogenes assessed, >99% of pharmacogenetic variants were covered at a diagnostic threshold of 20x. As previously mentioned, we excluded five genes from analysis due to poor (<20X) or variable coverage (see results, Figures 3, 4) in core pharmacogenomic variants. These variants (CYP1C19*17, UGT1A1 *80, CYP3A5*3, INFL3 variants–rs12980275, rs8099917, rs12979860, VKORC1 variant- rs9923231) are important phenotype defining variants, and as they occur in either promoter or intronic regions, they were not covered adequately by the exome capture kit utilized. It is important to note that next-generation reagent companies are now realizing the importance of covering clinically important intronic and intergenic variants and now offer extended exome capture kits, including important pharmacogenomic regions.

This analysis of 1,777 exomes from a predominantly Hispanic cohort revealed differences in the frequency of pharmacogenomic phenotypes when compared to those previously reported in the PharmGKB database (Hewett et al., 2002). Focusing on the AMR super population group, for *CYP2B6*, the exome data showed a higher proportion of poor metabolizers (13.2%) compared to that reported in PharmGKB (9.5%) and a lower frequency of rapid metabolizers (5.3% vs. 12.3%). In the case of *CYP2D6*, normal metabolizers were more prevalent in exomes (64.3%) relative to PharmGKB (54.6%), while exomes exhibited a reduced prevalence of ultrarapid metabolizers (0.2% vs. 3.8%). The lack of genetic reference data on admixed American populations (Secolin et al., 2019), particularly those with significant Hispanic ancestry, presents a critical challenge in pharmacogenomics (Zhang et al., 2019). Historically, the majority of pharmacogenomic studies have focused on individuals of European descent, with recent estimates suggesting that up to 86% of genomic research has been conducted in these populations (Popejoy, 2019).

### Limitations and future directions

There are limitations to note from our analysis.

This study provides valuable pharmacogenomic insights in an admixed population; however, its primary focus was on evaluating Dragen and PyPGx calls for known pharmacogenomic variants using standard clinical pipelines rather than assessing the impact of ancestry-specific reference genomes or graph genomes. Given that clinical pharmacogenomic databases such as PharmGKB and CPIC rely on hg38, and clinical sequencing workflows predominantly utilize this reference, we aligned our analysis with current clinical standards. While ancestry-aware approaches, such as graph genomes or population-specific references, may offer additional insights for certain genes, their implementation in clinical pharmacogenomics remains limited. Future work could explore the added benefits of these approaches in refining PGx variant interpretation across diverse populations. In addition, this study was designed to evaluate how Dragen and PyPGx bioinformatically predict known pharmacogenomic genotypes and phenotypes, rather than to identify novel or rare variants with potential pharmacogenomic impact. While our dataset may include sequencing data that could support such an analysis, our approach specifically focused on assessing these tools against established pharmacogenomic alleles as curated in databases such as PharmVar, PharmGKB, and CPIC. As a result, this study does not explore the potential contribution of novel or rare variants, including stop-gain/loss and frameshift mutations, which may have functional implications for drug response. Future research incorporating functional validation and expanded cohort analyses will be necessary to fully characterize the impact of rare pharmacogenomic variants, particularly in underrepresented populations such as the AMR cohort.

The exclusion of five important pharmacogenes from our actionability analysis is a major limitation. However, for the purposes of accurate phenotype assignment, these genes had to be excluded. The use of genomes will provide accurate pharmacogenetic information for alleles missed by exomes, as well as accurate detection of structural variation in pharmacogenes such as *CYP2D6* and *CYP2B6.* Further, it is important to note that genetic ancestry tools rely on reference populations such as the 1000 Genomes database. While such databases have improved representation from diverse populations, they could still be enhanced (Bolnick et al., 2007; Royal et al., 2010). Further, grouping populations into AMR as a single category can be misleading due to the substantial genetic diversity within this group. The grouping “AMR” encompasses individuals from various regions, including Central and South America, the Caribbean, and even parts of Europe (Maldonado et al., 2023; Koehl and Long, 2018). This diversity-within-diversity may result in significant differences in genetic markers, health risks, and responses to medications. Finally, the differences in phenotypic frequencies among the three datasets—our study population, PharmGKB (Hewett et al., 2002), and a recent pharmacogenetic study assessing 1000 Genomes data (Sherman et al., 2024) (Table 1) could be attributed to several factors. Our study population, primarily Hispanic reflects a unique genetic admixture characteristic of the diverse populations in the Southwestern United States. This admixture likely results in allele frequencies that differ from the more homogenous ancestry-specific datasets of PharmGKB and 1000 Genomes. In addition, methodological factors such as sample size, bioinformatic pipelines, criteria for genetic ancestry estimation or self-reported ancestry are likely contributing factors.

## Conclusion

In a recent white paper by the Pediatric Task Team of the Global Alliance for Genomics and Health, Friedman et al. (2024) evaluated the ethical, clinical, and policy considerations of actively screening and reporting secondary pharmacogenomic variants in children undergoing diagnostic genome-wide sequencing for suspected genetic diseases. The white paper highlighted potential benefits such as reducing adverse drug reactions and improving treatment efficacy, while addressing concerns about data storage and equitable access (Friedman et al., 2024). Analyzing pharmacogenomic markers as part of clinical exome or genome testing will allow clinicians to personalize medication regimens based on an individual’s own genetic makeup, improving therapeutic outcomes and minimizing adverse drug reactions. While exomes exhibit coverage gaps, particularly in non-coding regions crucial for correct phenotype assignment, application of exomes and genomes to both diagnose genetic disorders *and* inform pharmacogenomic strategies significantly broadens their clinical utility, making them powerful tools in molecular diagnostics. These results highlight and reinforce the staggering fact that close to 100% of patients undergoing pharmacogenetic screening have at least one actionable variant. This underscores the need to readily offer pharmacogenomic testing and integrate comprehensive pharmacogenomic data into EMRs with CDS systems to ensure accurate, safe and personalized therapeutic recommendations.

## Data Availability

A complete anonymized pharmacogenomic dataset including genotypes and phenotypes, and all further analyses as indicated in the manuscript are available via github. (https://github.com/Pharmacogenetecist/WESPGX_2024). Further requests for specific data can be forwarded to the corresponding authors.
