# Correction: Clinical impact of pharmacogenomics in pediatric care: insights extracted from clinical exome sequencing

**DOI:** 10.3389/fgene.2025.1639666

**Published:** 2025-07-08

**Authors:** Simran Maggo, Yachen Pan, Dejerianne Ostrow, Jenny Q. Nguyen, Jaclyn A. Biegel, Matthew A. Deardorff, Xiaowu Gai

**Affiliations:** ^1^ Bernard J. Dunn School of Pharmacy, Shenandoah University, Winchester, VA, United States; ^2^ Center for Personalized Medicine, Department of Pathology and Laboratory Medicine, Children’s Hospital Los Angeles, Los Angeles, CA, United States; ^3^ Personalized Care Program, Department of Pathology and Laboratory Medicine, Children’s Hospital Los Angeles, Los Angeles, CA, United States; ^4^ Department of Pathology and Laboratory Medicine, Keck School of Medicine, University of Southern California, Los Angeles, CA, United States; ^5^ Linda T. and John A. Mellowes Center for Genomic Sciences and Precision Medicine, Medical College of Wisconsin, Milwaukee, WI, United States; ^6^ Department of Pediatrics, Medical College of Wisconsin, Milwaukee, WI, United States

**Keywords:** pharmacogenomic (PGx) research, pharmacogenetic testing, bioinformatic analyses, admixed american pharmacogenomics, pharmacogenomics data

In the published article, there was an error in Table 1 as published. Table one, included two instances of SLCO1B1 The first instance should be CYP2B6**.** The corrected Table1 and its caption appear below.

**TABLE 1:** Phenotype frequency (%) comparison of the Whole Exome Sequencing cohort (CES) against data published on the PharmGKB (PGKB) website and a recent publication (LIT) (Tremmel et al., 2023). For population comparisons, AMR (Admixed American) from CES was compared against LAT (Latino) from PGKB due to differences in biogeographical classifications. Blank values for, PGKB, or LIT indicate values not available at the time of publication.

CYP2B6	AFR			AMR			EAS			EUR			SAS		
	CES	PGKB	LIT	CES	PGKB	LIT	CES	PGKB	LIT	CES	PGKB	LIT	CES	PGKB	LIT
Normal Metabolizer	22	33.4	25.4	34.2	33.6	35.4	54.5	48.6	51.2	48.2	43	51.2	34.2	42.2	30.9
Intermediate Metabolizer	54.9	46.6	47.7	42.1	42.2	43.5	26.9	32.6	33.1	37.1	38	35.1	42.1	36.5	45.4
Poor Metabolizer	20.9	15.7	21.5	13.2	9.5	16.4	7.1	4.4	5.2	6.2	7.4	5.8	13.2	6	15.1
Rapid Metabolizer	1.1	1.2	2.7	5.3	12.3	1.4	10.3	11.7	9.3	5.2	5.4	4.8	5.3	12.9	6.5
Ultrarapid Metabolizer	0	0	0	2.6	1.1	0	0	0.7	0.4	0.2	0.2	0.4	2.6	1	0.0
Indeterminate	1.1	3.1	2.2	2.6	1.4	3.1	1.3	2	0.8	3.1	6.1	2.8	2.6	1.5	2.0

CYP2D6	AFR			AMR			EAS			EUR			SAS		
	CES	PGKB	LIT	CES	PGKB	LIT	CES	PGKB	LIT	CES	PGKB	LIT	CES	PGKB	LIT
Normal Metabolizer (1.25 ≤ x ≤ 2.25)	62.6	31.4	50.1	64.3	54.6	65.1	62.8	52.1	52.4	49.6	48.2	48.4	65.8	57.8	59.7
Intermediate Metabolizer (0 < x < 1.25)	22	59.7	36.2	25.5	34.2	24.2	28.8	40	44.6	36.8	39.3	38.4	23.7	28.4	25.4
Poor Metabolizer (0)	3.3	2.3	2.1	3.6	3.1	3.5	1.9	0.8	0.0	7.1	6.5	6.2	2.6	2.4	2.9
Ultrarapid Metabolizer (>2.25)	0	2.6	5.1	0.2	3.8	3.2	0	0.8	1.0	0	2.3	3.0	0	1.5	1.2
Indeterminate	12.1		6.5	6.3		4.0	6.4		2.0	6.4		4.0	7.9		10.8

ABCG2	AFR			AMR			EAS			EUR			SAS		
	CES	PGKB	LIT	CES	PGKB	LIT	CES	PGKB	LIT	CES	PGKB	LIT	CES	PGKB	LIT
Normal Function	92.3	93.1	98.2	61.8	60.2	79.3	50	48	72.0	81.7	80.4	89.0	86.8	82.2	81.4
Decreased Function	7.7	6.7	1.8	32.7	34.8	19.9	42.9	42.6	23.8	17.1	18.6	10.2	10.5	16.9	18.2
Poor Function	0	0.1	0.0	5.5	5	0.9	7.1	9.4	4.2	1.2	1.1	0.8	2.6	0.9	0.4

CYP2C9	AFR			AMR			EAS			EUR			SAS		
	CES	PGKB	LIT	CES	PGKB	LIT	CES	PGKB	LIT	CES	PGKB	LIT	CES	PGKB	LIT
Normal Metabolizer	79.1	75.9	78.5	76.6	74.3	72.9	89.1	83.8	91.7	62	62.8	63.1	60.5	59.6	68.7
Intermediate Metabolizer	20.9	23.6	21.2	23.1	24.6	26.2	9.6	15.2	8.1	35.6	34.5	34.7	36.8	36.2	27.2
Poor Metabolizer	0	0.5	0.3	0.2	1	0.9	0	0.6	0.2	2.1	2.6	2.2	2.6	3.8	2.7
Indeterminate	0	0	0.0	0.2	0.1	0.0	1.3	0.5	0.0	0.2	0.1	0.0	0	0.4	1.4

SLCO1B1	AFR			AMR			EAS			EUR			SAS		
	CES	PGKB	LIT	CES	PGKB	LIT	CES	PGKB	LIT	CES	PGKB	LIT	CES	PGKB	LIT
Normal Function	69.2	98	51.7	76.7		50.4	70.5	75.7	72.2	63.2	65.6	39.2	89.5	86.5	72.6
Decreased Function	13.2	2	1.4	18.7		19.9	26.3	21.8	19.8	25.7	28.3	20.1	7.9	13	7.0
Possible Decreased Function	1.1	0	5.1	0.6		0.0	0.6	0.1	0.0	0.7	0	0.0	0	0	0.0
Poor Function	0	0	0.2	0.8		1.7	1.9	1.6	2.0	3.8	2.9	2.0	0	0.5	0.4
Increased Function	2.2	0		0.7			0	0		3.3	3		2.6	0	
Indeterminate	14.3	0	33.3	2.6		13.8	0.6	0.8	5.4	3.3	0.1	18.7	0	0.1	8.4

HLA - Allele	AFR			AMR			EAS			EUR			SAS		
	CES	PGKB		CES	PGKB		CES	PGKB		CES	PGKB		CES	PGKB	
HLA-A*31:01	0	0.99		1.63	4.52		0.96	3.45		0.59	2.64		0	3.3	
HLA-B*15:02	0.55	0.1		0	0.03		6.73	4.56		0	0.01		0	2.59	
HLA-B*57:01	0	0.61		0.56	1.39		0.32	0.98		3.09	3.6		5.26	6.81	
HLA-B*58:01	2.75	3.82		1.07	1.35		3.85	6.04		0.48	0.77		3.95	4.19	

The authors apologize for this error and state that this does not change the scientific conclusions of the article in any way. The original article has been updated.

