# Supplementary material for: Clinical impact of pharmacogenomics in pediatric care: insights extracted from clinical exome sequencing
Source: Front Genet. 2025 May 29;16:1574325. doi: 10.3389/fgene.2025.1574325 (PMC12159002; doi:10.3389/fgene.2025.1574325)
Supplement: Supplementary file 1 [file DataSheet7.pdf]

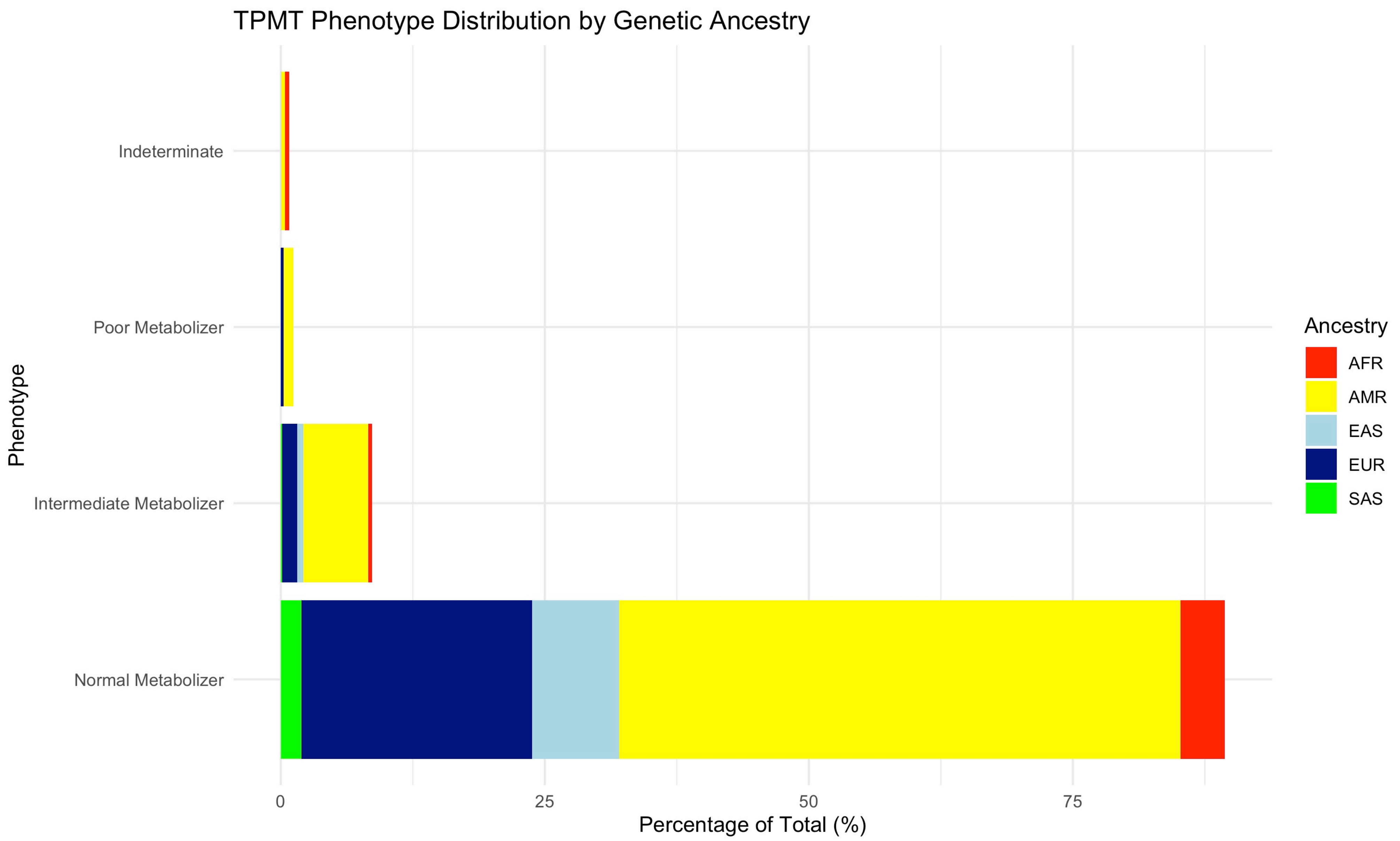
TPMT Phenotype Distribution by Genetic Ancestry
Indeterminate
Poor Metabolizer
Intermediate Metabolizer
Normal Metabolizer
Phenotype
0
25
50
75
Percentage of Total (%)
Ancestry
AFR
AMR
EAS
EUR
SAS

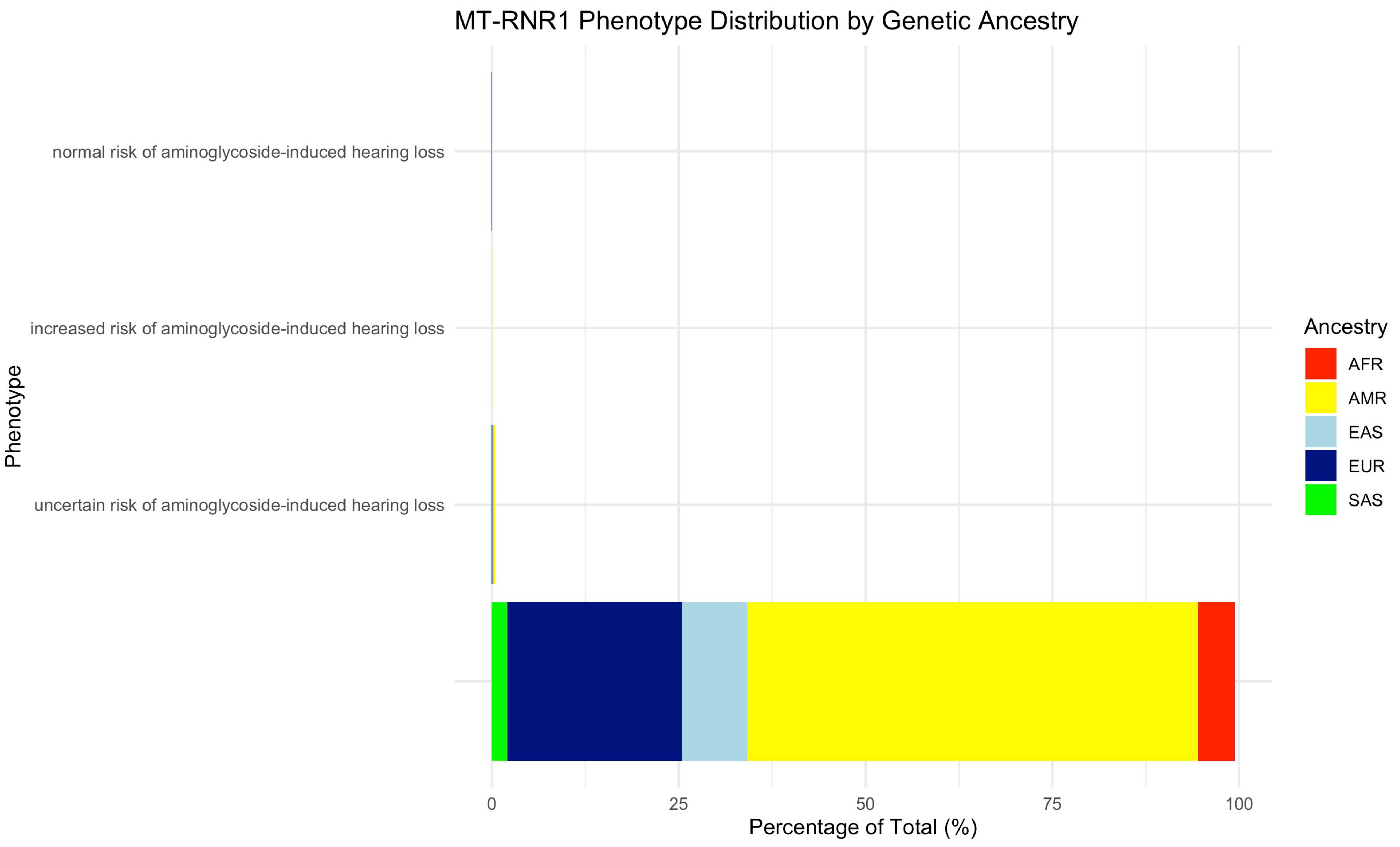
MT-RNR1 Phenotype Distribution by Genetic Ancestry
normal risk of aminoglycoside-induced hearing loss
increased risk of aminoglycoside-induced hearing loss
uncertain risk of aminoglycoside-induced hearing loss
Phenotype
0
25
50
75
100
Percentage of Total (%)
Ancestry
AFR
AMR
EAS
EUR
SAS

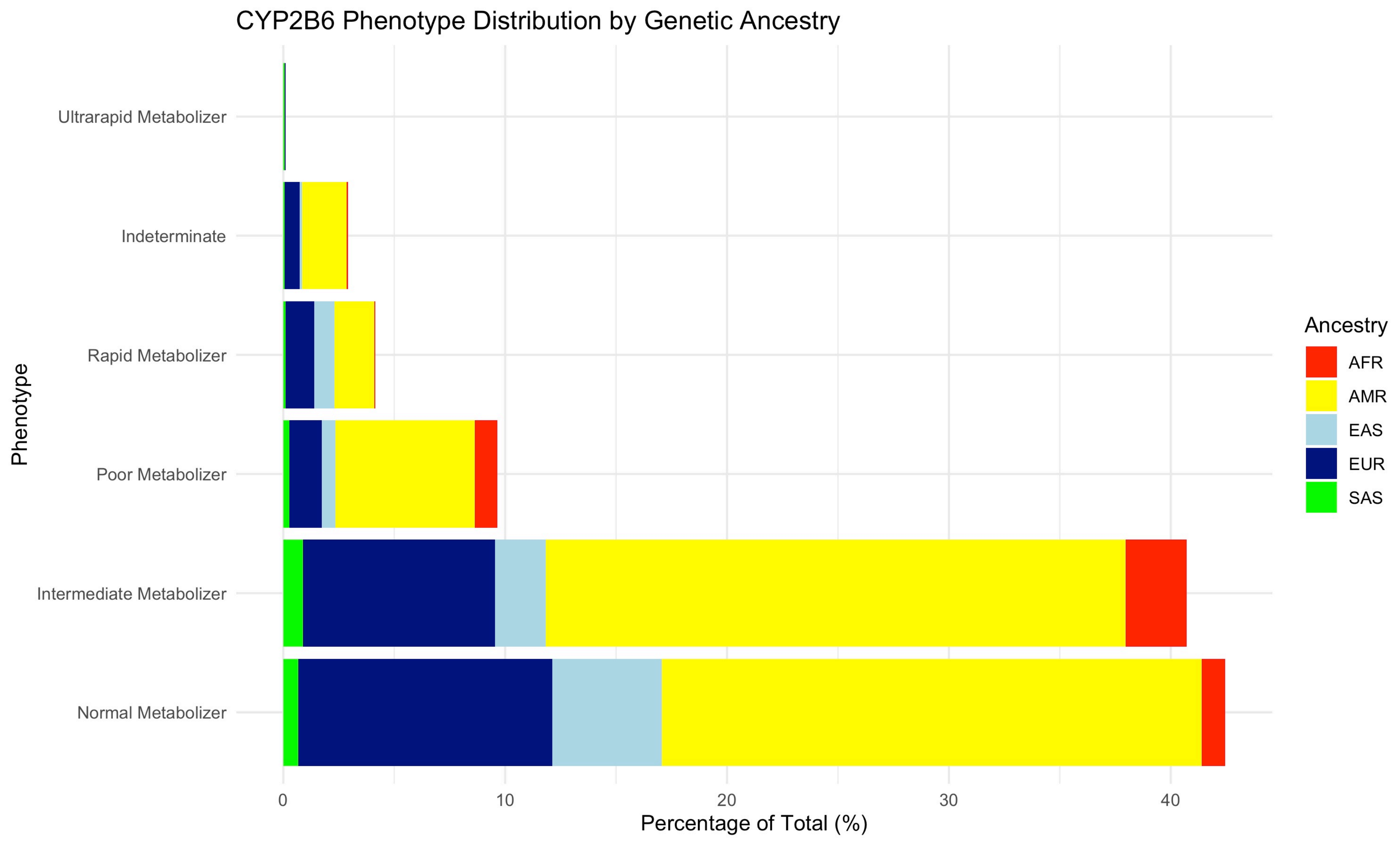
CYP2B6 Phenotype Distribution by Genetic Ancestry
Phenotype
Ultrarapid Metabolizer
Indeterminate
Rapid Metabolizer
Poor Metabolizer
Intermediate Metabolizer
Normal Metabolizer
0
10
20
30
40
Percentage of Total (%)
Ancestry
AFR
AMR
EAS
EUR
SAS

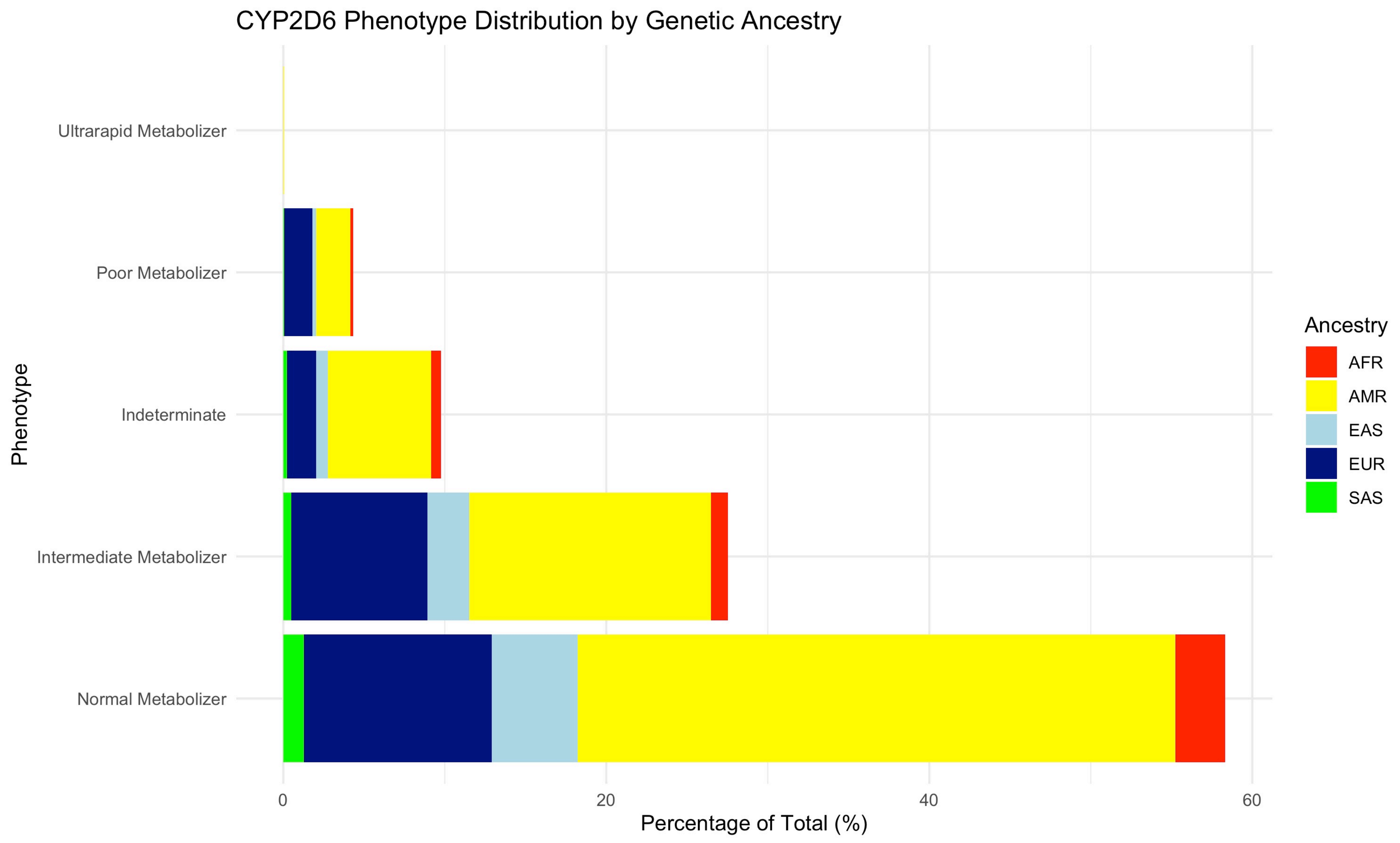
CYP2D6 Phenotype Distribution by Genetic Ancestry
Phenotype
Ultrarapid Metabolizer
Poor Metabolizer
Indeterminate
Intermediate Metabolizer
Normal Metabolizer
0
20
40
60
Percentage of Total (%)
Ancestry
AFR
AMR
EAS
EUR
SAS

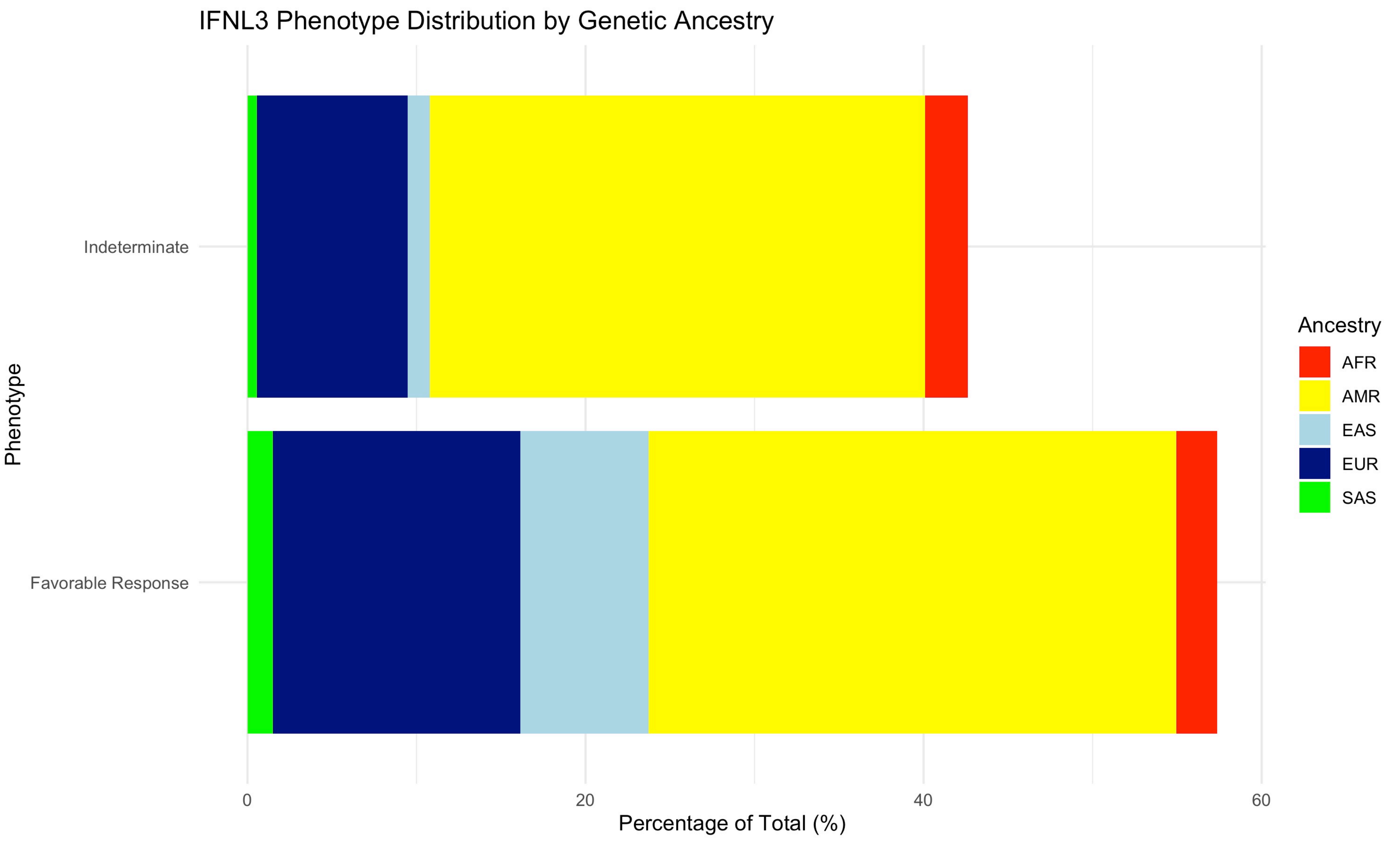
IFNL3 Phenotype Distribution by Genetic Ancestry
Phenotype
Indeterminate
Favorable Response
0
20
40
60
Percentage of Total (%)
Ancestry
AFR
AMR
EAS
EUR
SAS

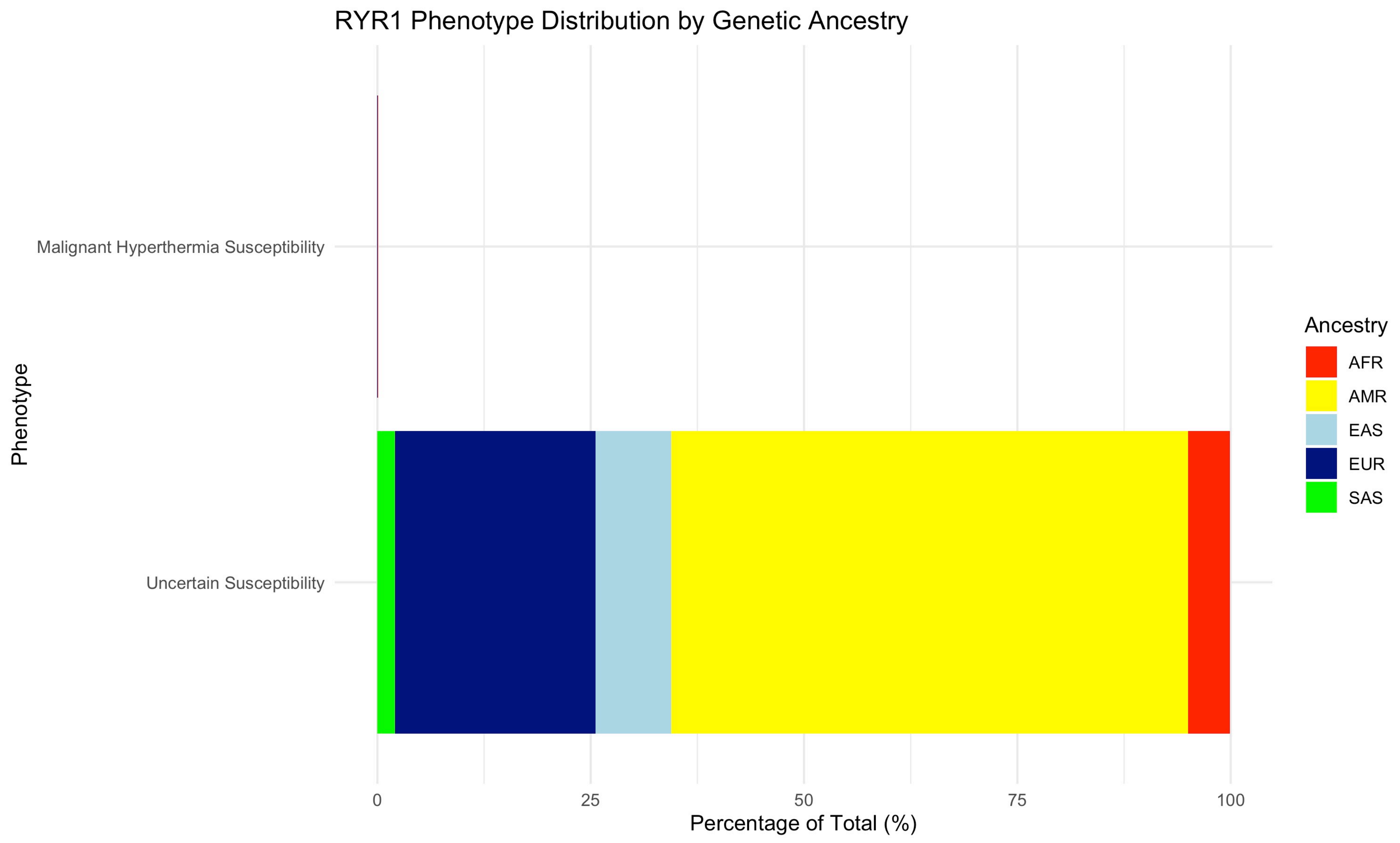
RYR1 Phenotype Distribution by Genetic Ancestry
Phenotype
Malignant Hyperthermia Susceptibility
Uncertain Susceptibility
0
25
50
75
100
Percentage of Total (%)
Ancestry
AFR
AMR
EAS
EUR
SAS

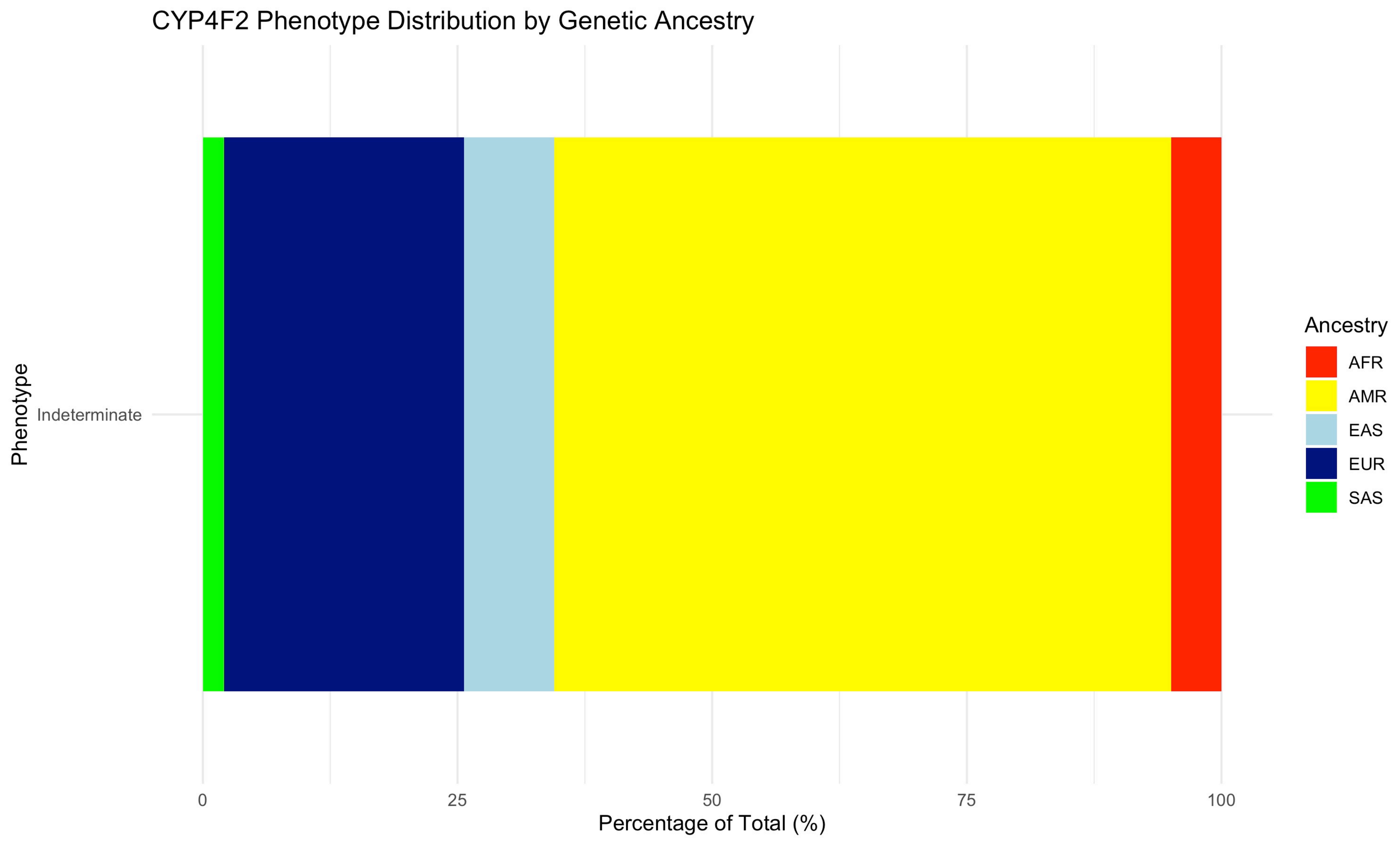
CYP4F2 Phenotype Distribution by Genetic Ancestry
Phenotype
Indeterminate
0
25
50
75
100
Percentage of Total (%)
Ancestry
AFR
AMR
EAS
EUR
SAS

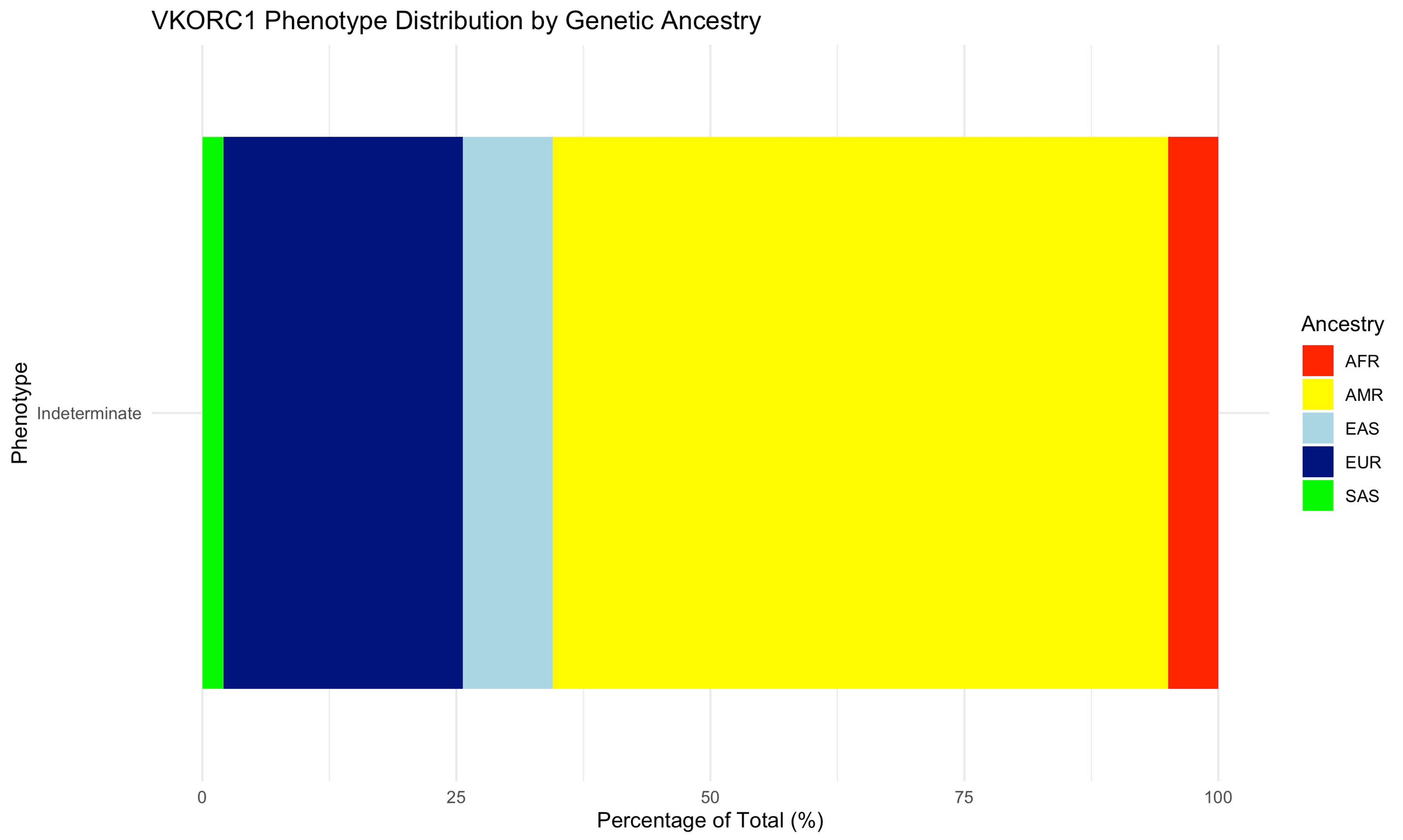

VKORC1 Phenotype Distribution by Genetic Ancestry
Phenotype
Indeterminate
0
25
50
75
100
Percentage of Total (%)
Ancestry
AFR
AMR
EAS
EUR
SAS

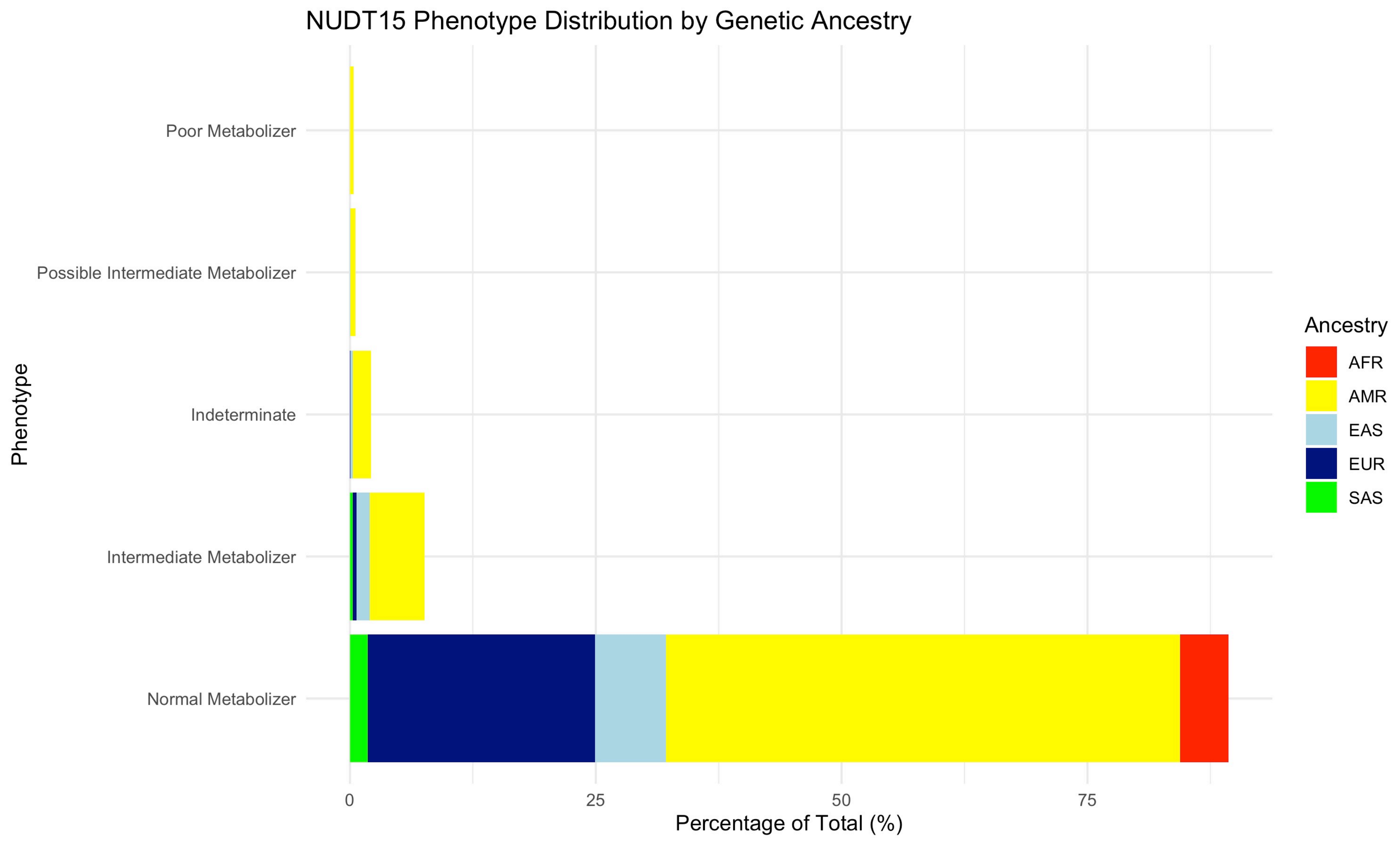
NUDT15 Phenotype Distribution by Genetic Ancestry
Phenotype
Poor Metabolizer
Possible Intermediate Metabolizer
Indeterminate
Intermediate Metabolizer
Normal Metabolizer
0
25
50
75
Percentage of Total (%)
Ancestry
AFR
AMR
EAS
EUR
SAS

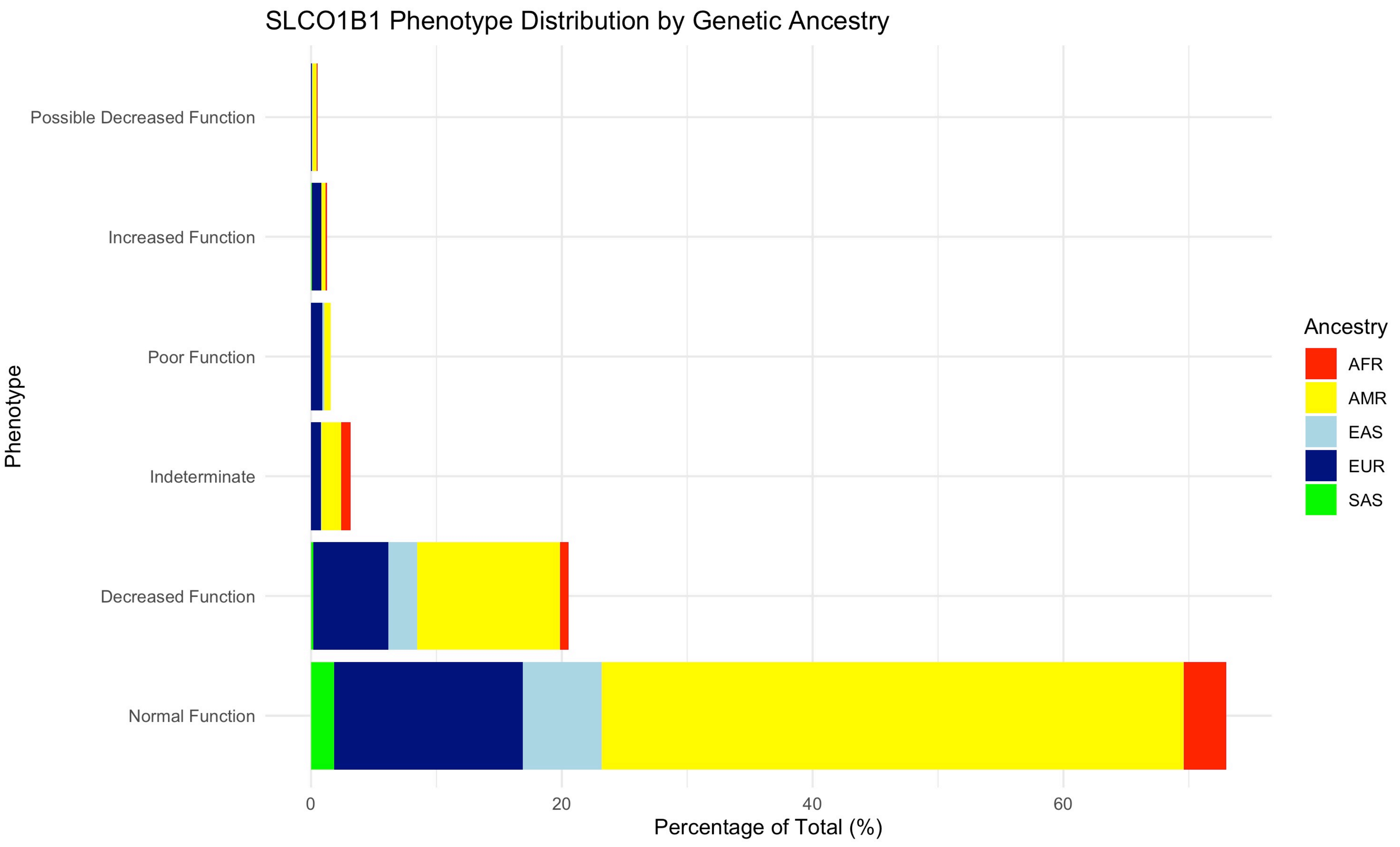
SLCO1B1 Phenotype Distribution by Genetic Ancestry
Phenotype
Possible Decreased Function
Increased Function
Poor Function
Indeterminate
Decreased Function
Normal Function
0
20
40
60
Percentage of Total (%)
Ancestry
AFR
AMR
EAS
EUR
SAS

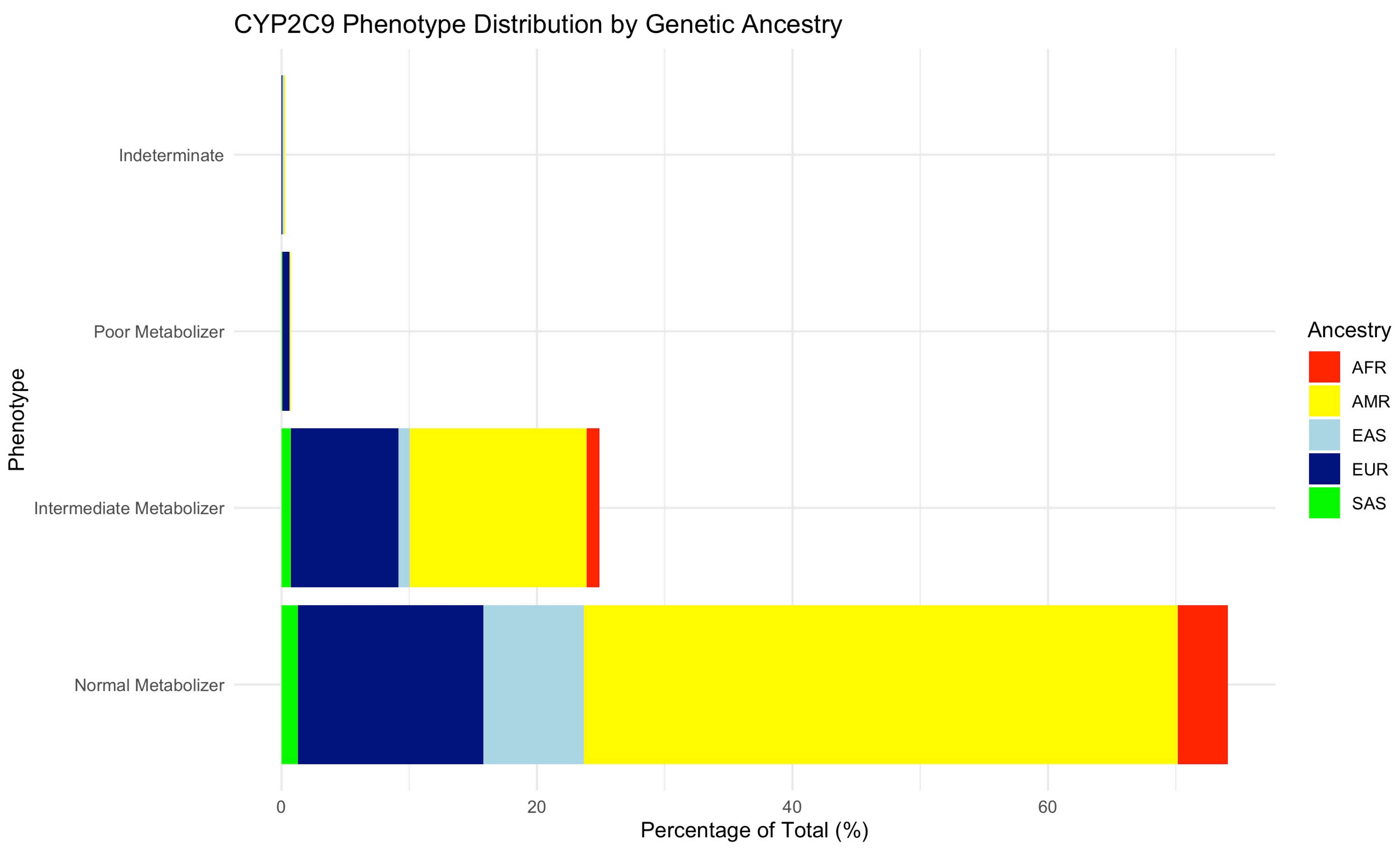
CYP2C9 Phenotype Distribution by Genetic Ancestry
Phenotype
Indeterminate
Poor Metabolizer
Intermediate Metabolizer
Normal Metabolizer
0
20
40
60
Percentage of Total (%)
Ancestry
AFR
AMR
EAS
EUR
SAS

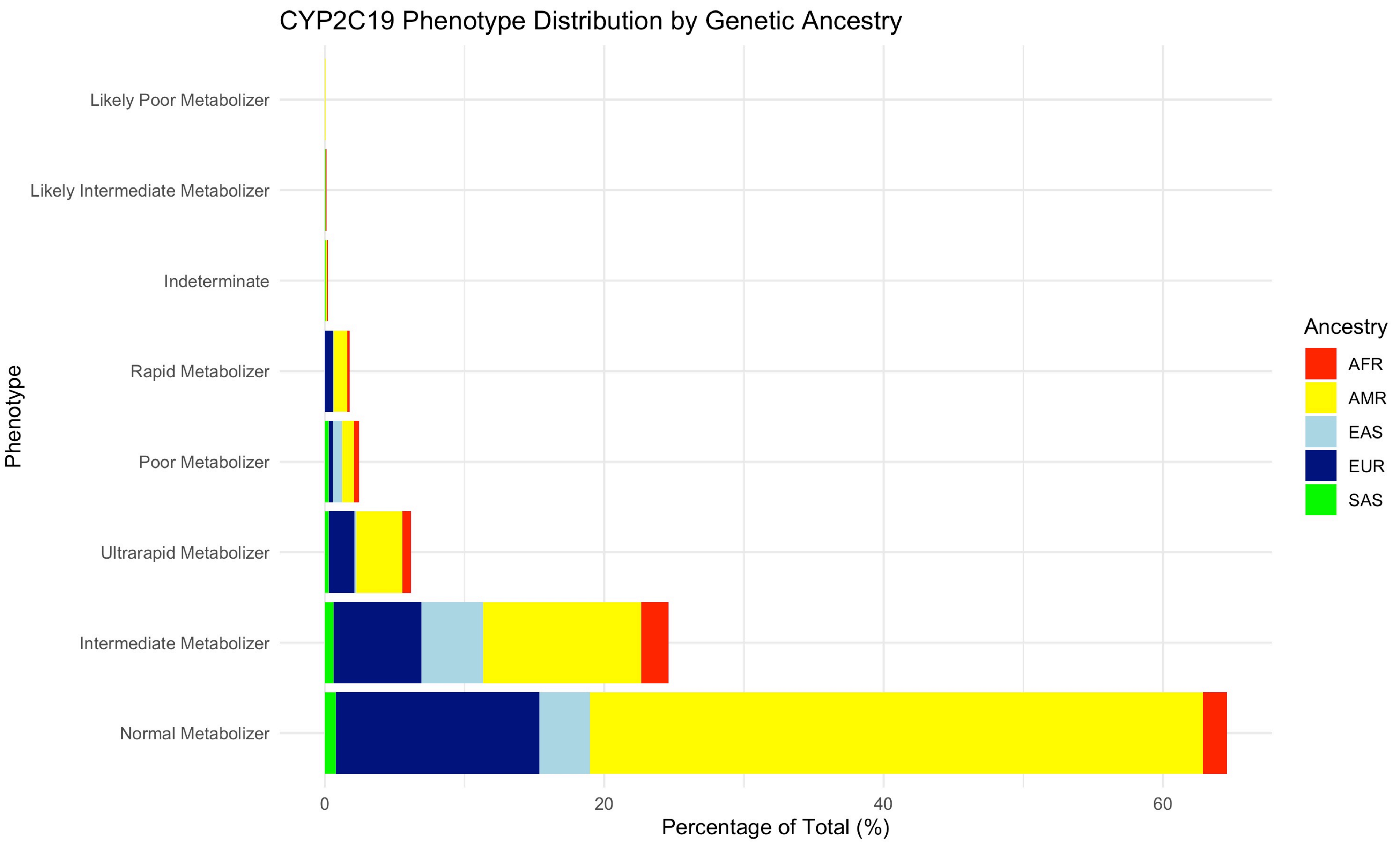
CYP2C19 Phenotype Distribution by Genetic Ancestry
Phenotype
Likely Poor Metabolizer
Likely Intermediate Metabolizer
Indeterminate
Rapid Metabolizer
Poor Metabolizer
Ultrarapid Metabolizer
Intermediate Metabolizer
Normal Metabolizer
0
20
40
60
Percentage of Total (%)
Ancestry
AFR
AMR
EAS
EUR
SAS

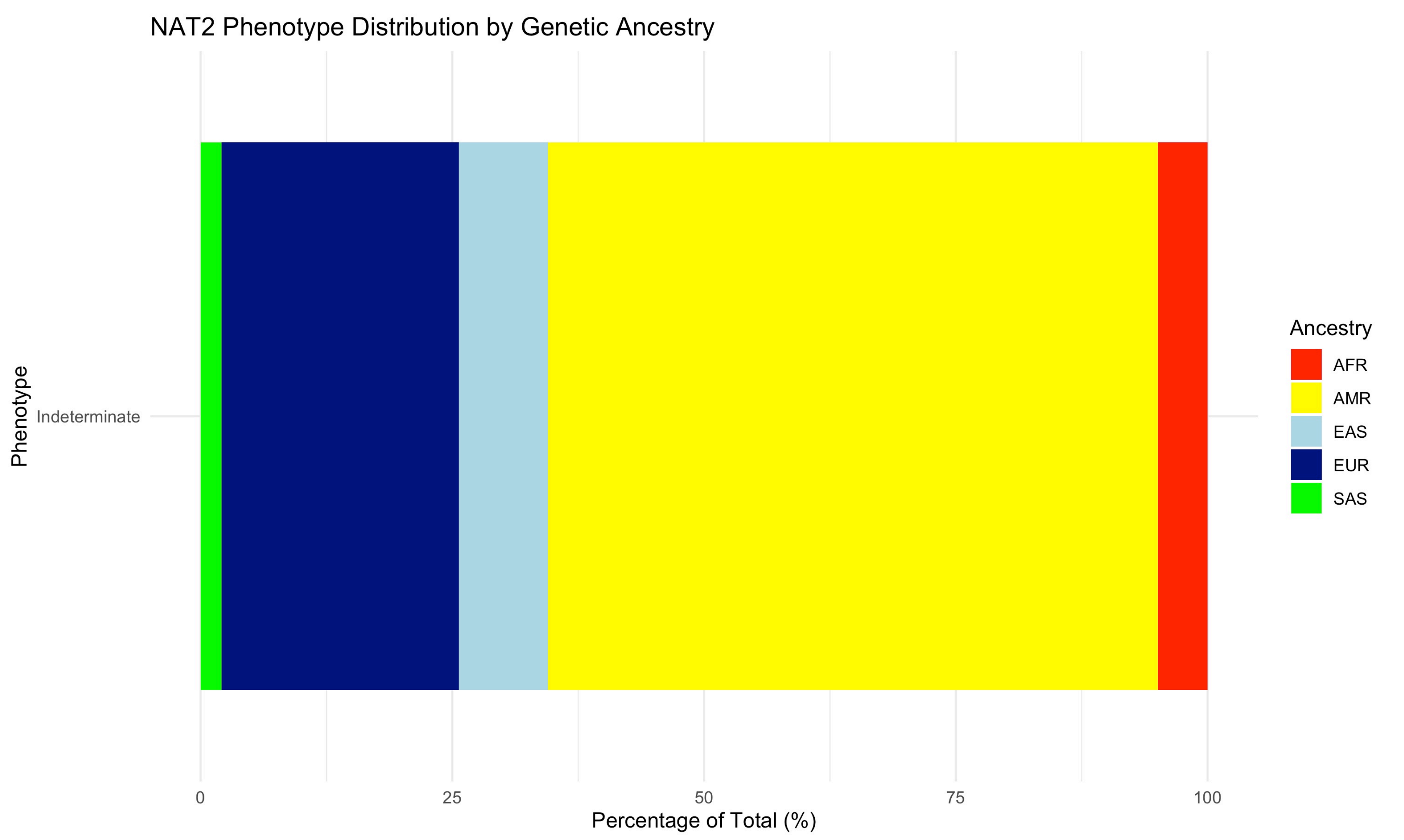
NAT2 Phenotype Distribution by Genetic Ancestry
Phenotype
Indeterminate
0
25
50
75
100
Percentage of Total (%)
Ancestry
AFR
AMR
EAS
EUR
SAS

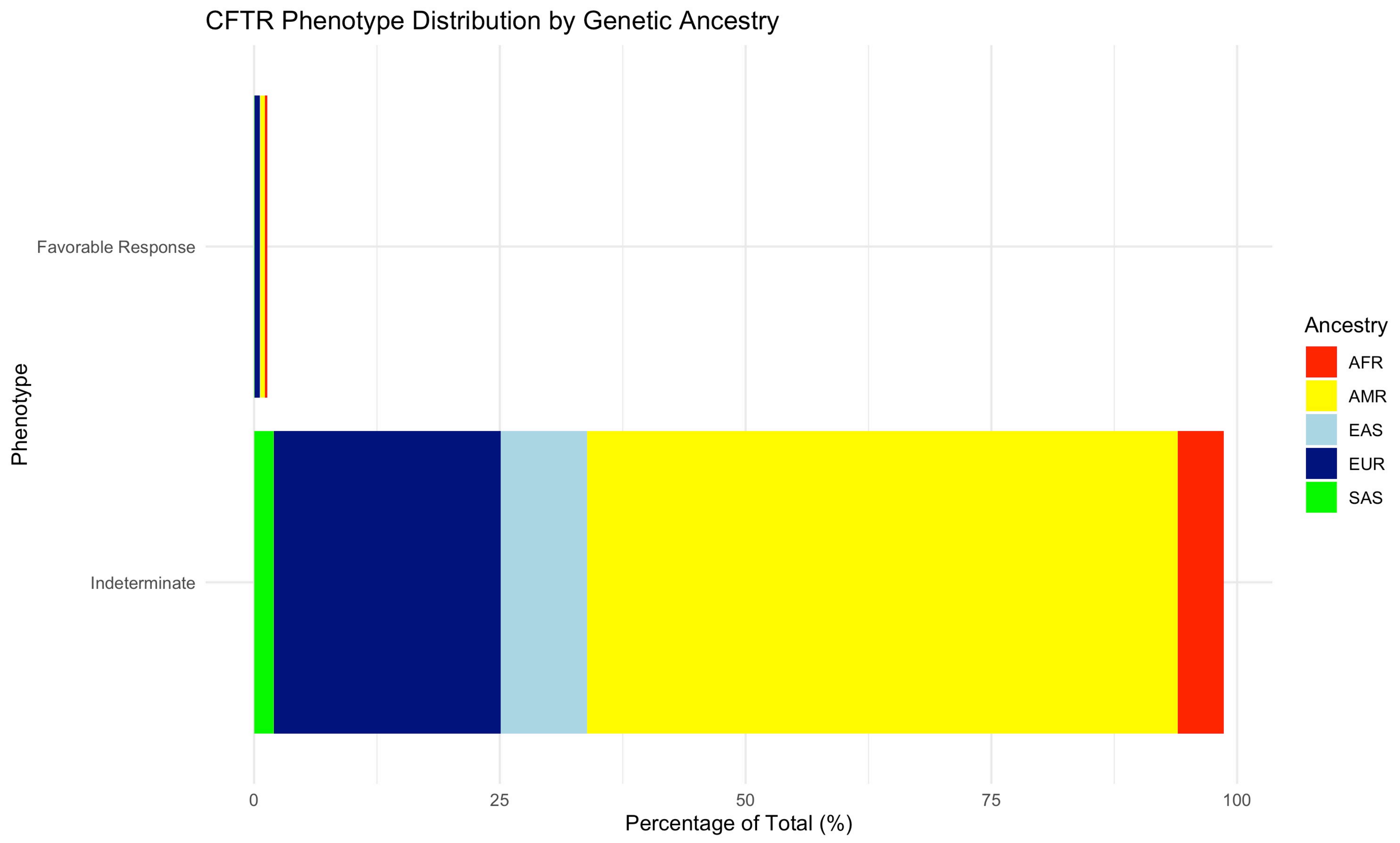
CFTR Phenotype Distribution by Genetic Ancestry
Phenotype
Favorable Response
Indeterminate
0
25
50
75
100
Percentage of Total (%)
Ancestry
AFR
AMR
EAS
EUR
SAS

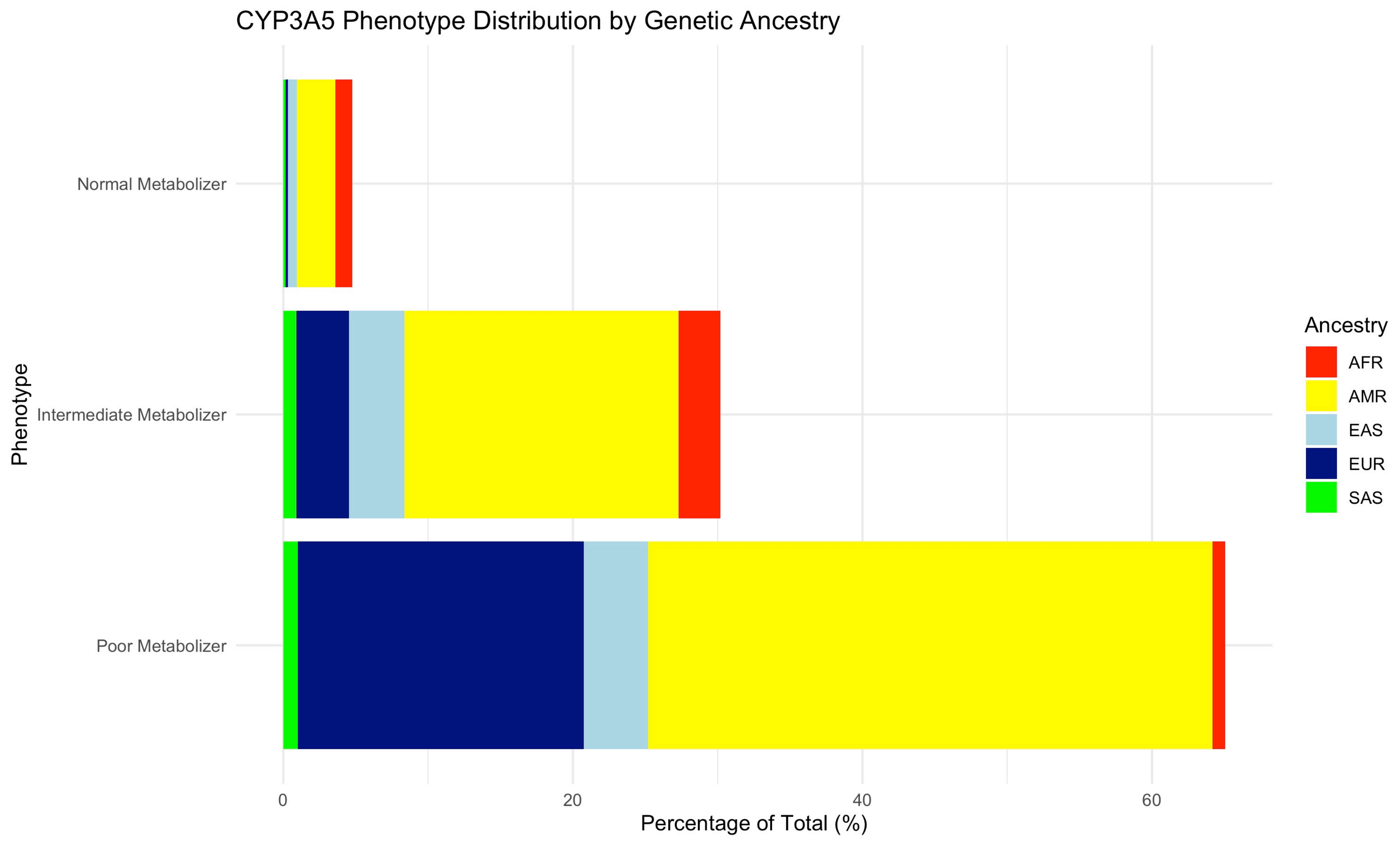
CYP3A5 Phenotype Distribution by Genetic Ancestry
Phenotype
Normal Metabolizer
Intermediate Metabolizer
Poor Metabolizer
0
20
40
60
Percentage of Total (%)
Ancestry
AFR
AMR
EAS
EUR
SAS

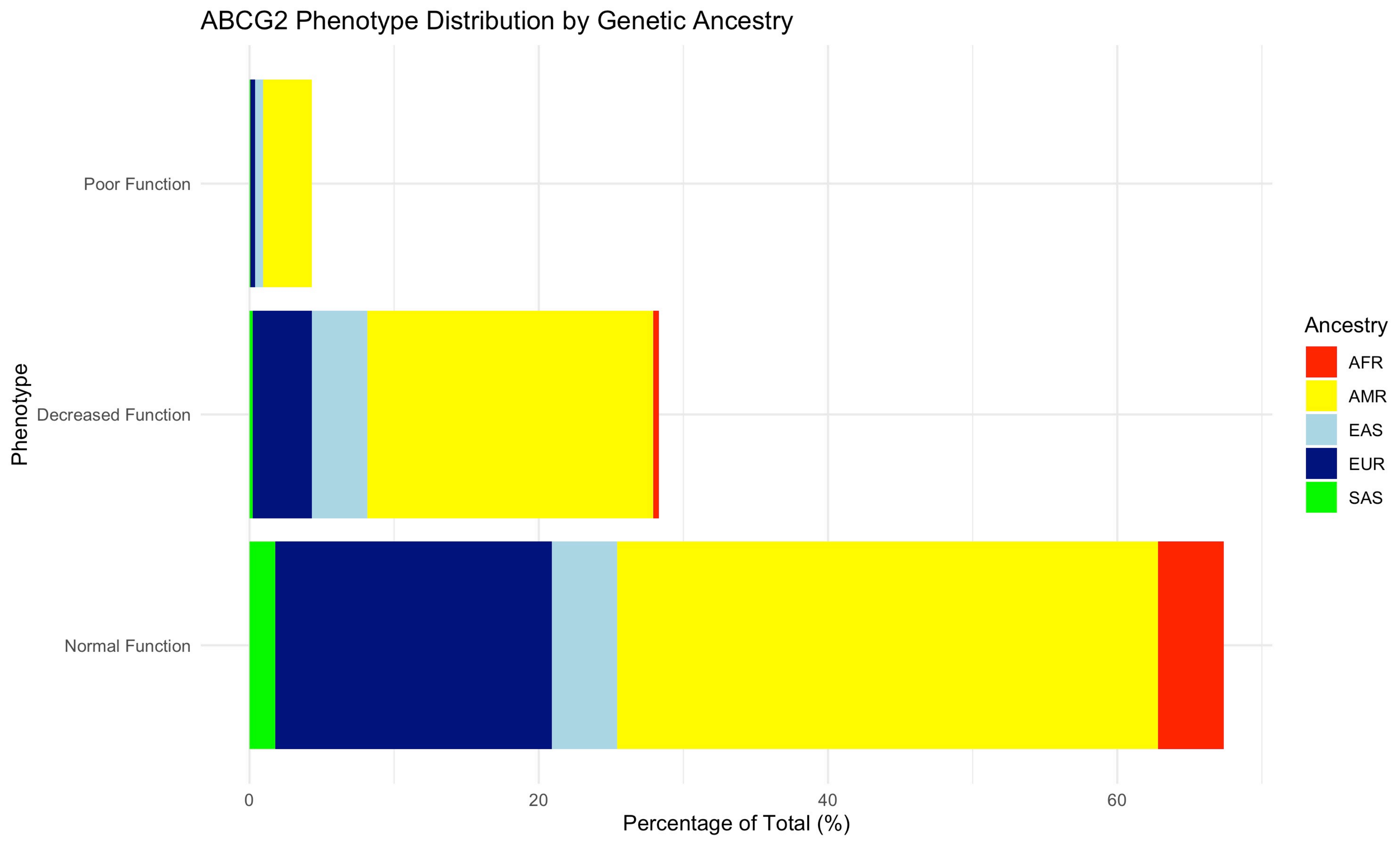
ABCG2 Phenotype Distribution by Genetic Ancestry
Phenotype
Poor Function
Decreased Function
Normal Function
0
20
40
60
Percentage of Total (%)
Ancestry
AFR
AMR
EAS
EUR
SAS

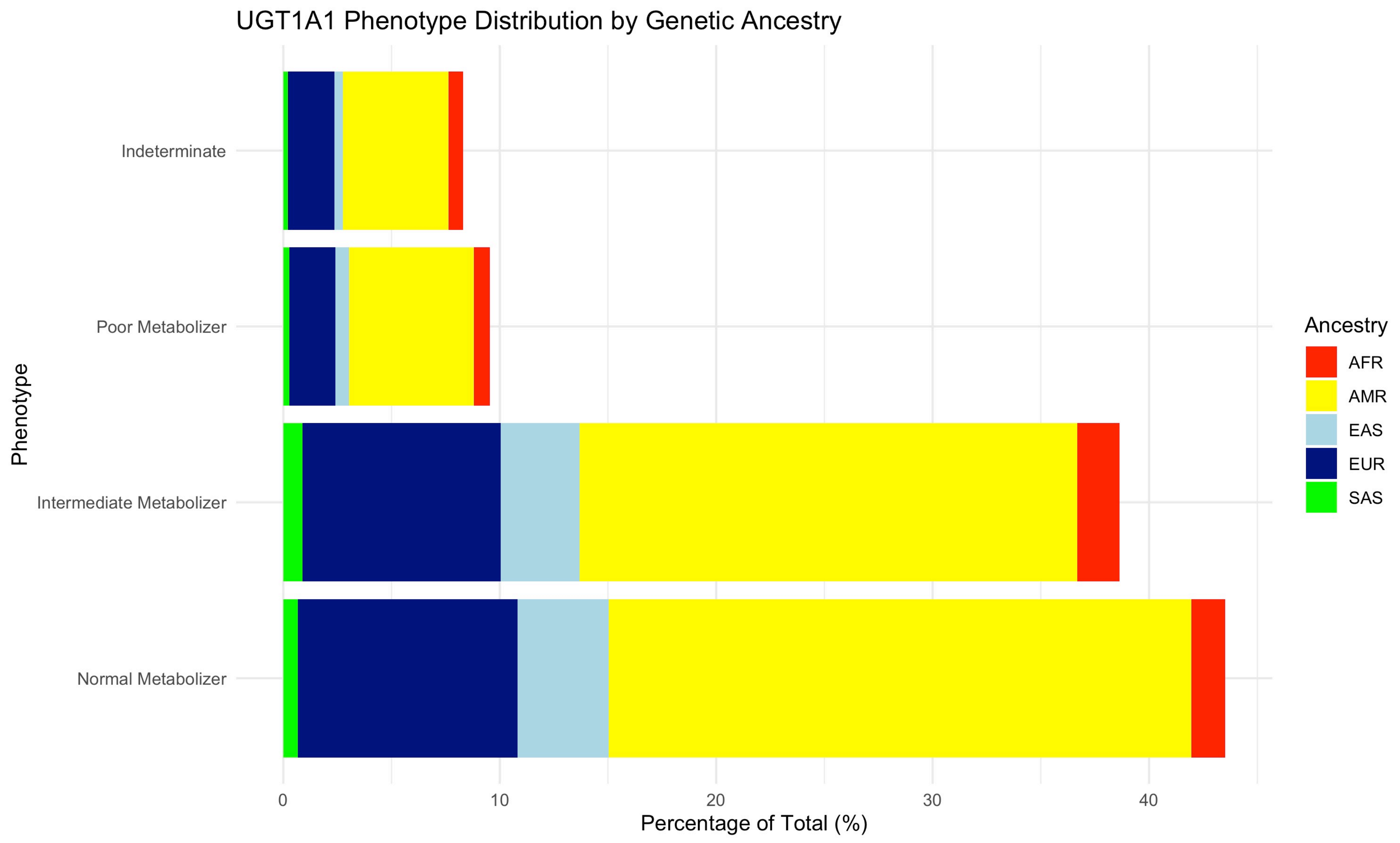
UGT1A1 Phenotype Distribution by Genetic Ancestry
Phenotype
Indeterminate
Poor Metabolizer
Intermediate Metabolizer
Normal Metabolizer
0
10
20
30
40
Percentage of Total (%)
Ancestry
AFR
AMR
EAS
EUR
SAS

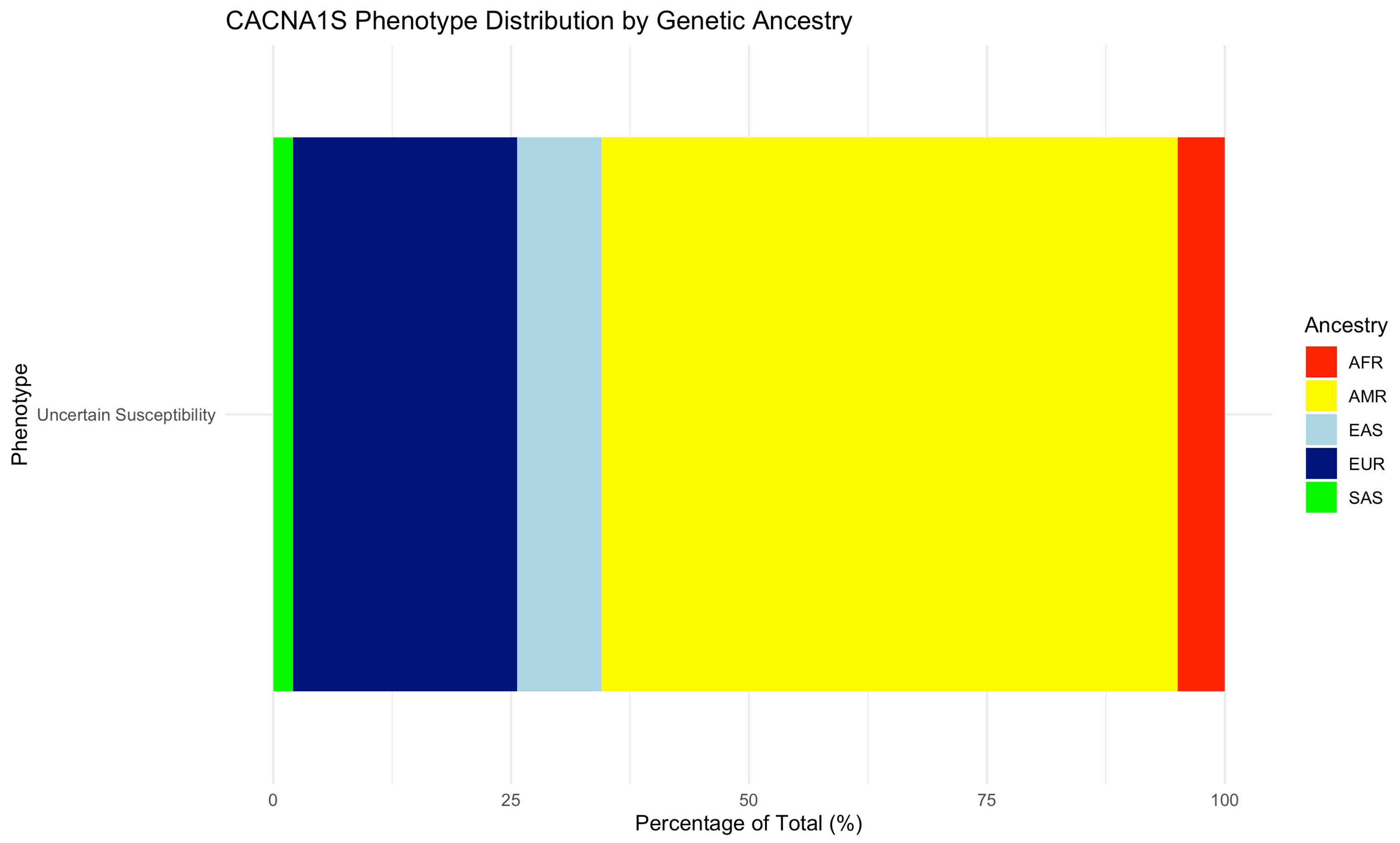
CACNA1S Phenotype Distribution by Genetic Ancestry
Phenotype
Uncertain Susceptibility
0
25
50
75
100
Percentage of Total (%)
Ancestry
AFR
AMR
EAS
EUR
SAS

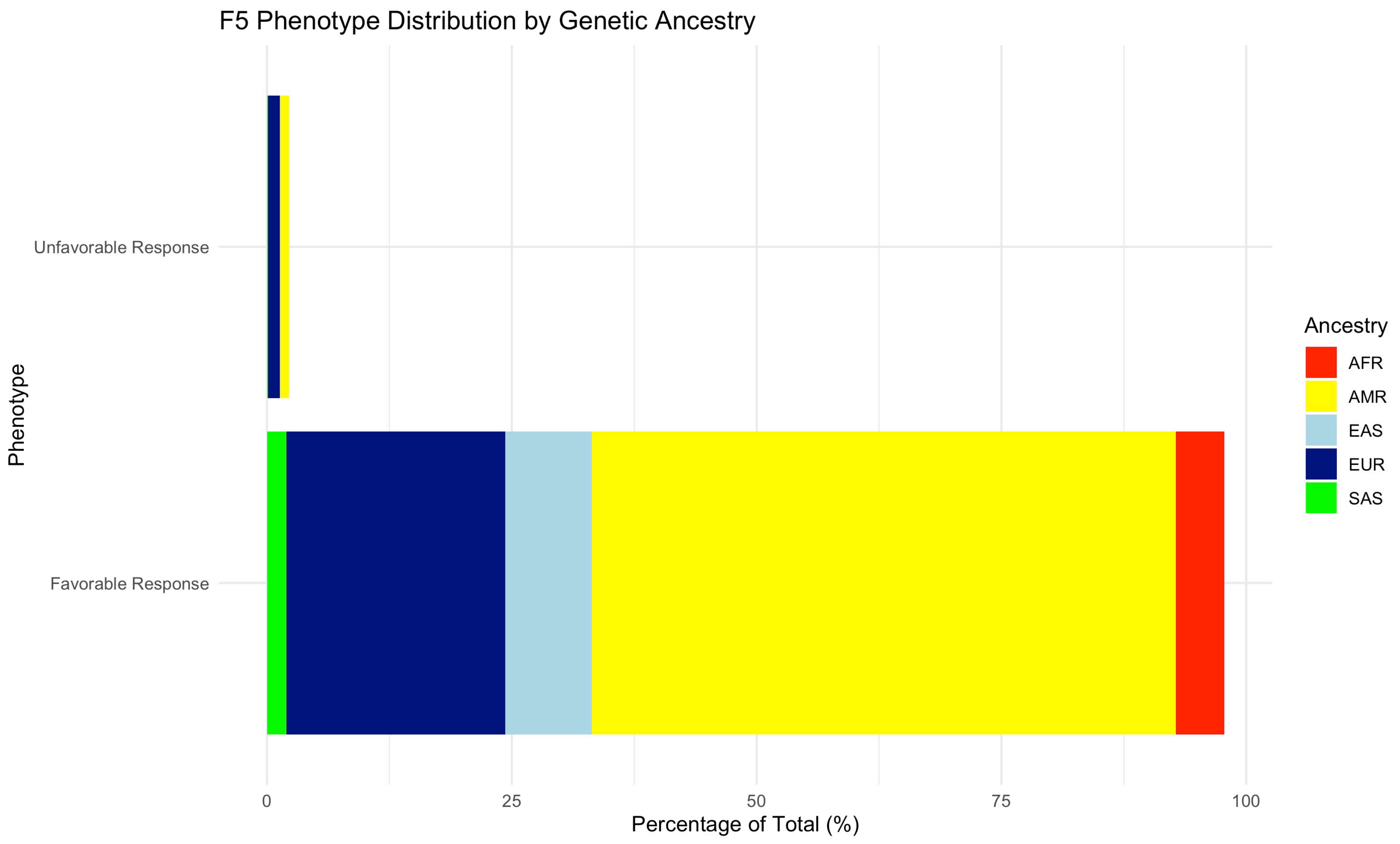
F5 Phenotype Distribution by Genetic Ancestry
Phenotype
Unfavorable Response
Favorable Response
0
25
50
75
100
Percentage of Total (%)
Ancestry
AFR
AMR
EAS
EUR
SAS

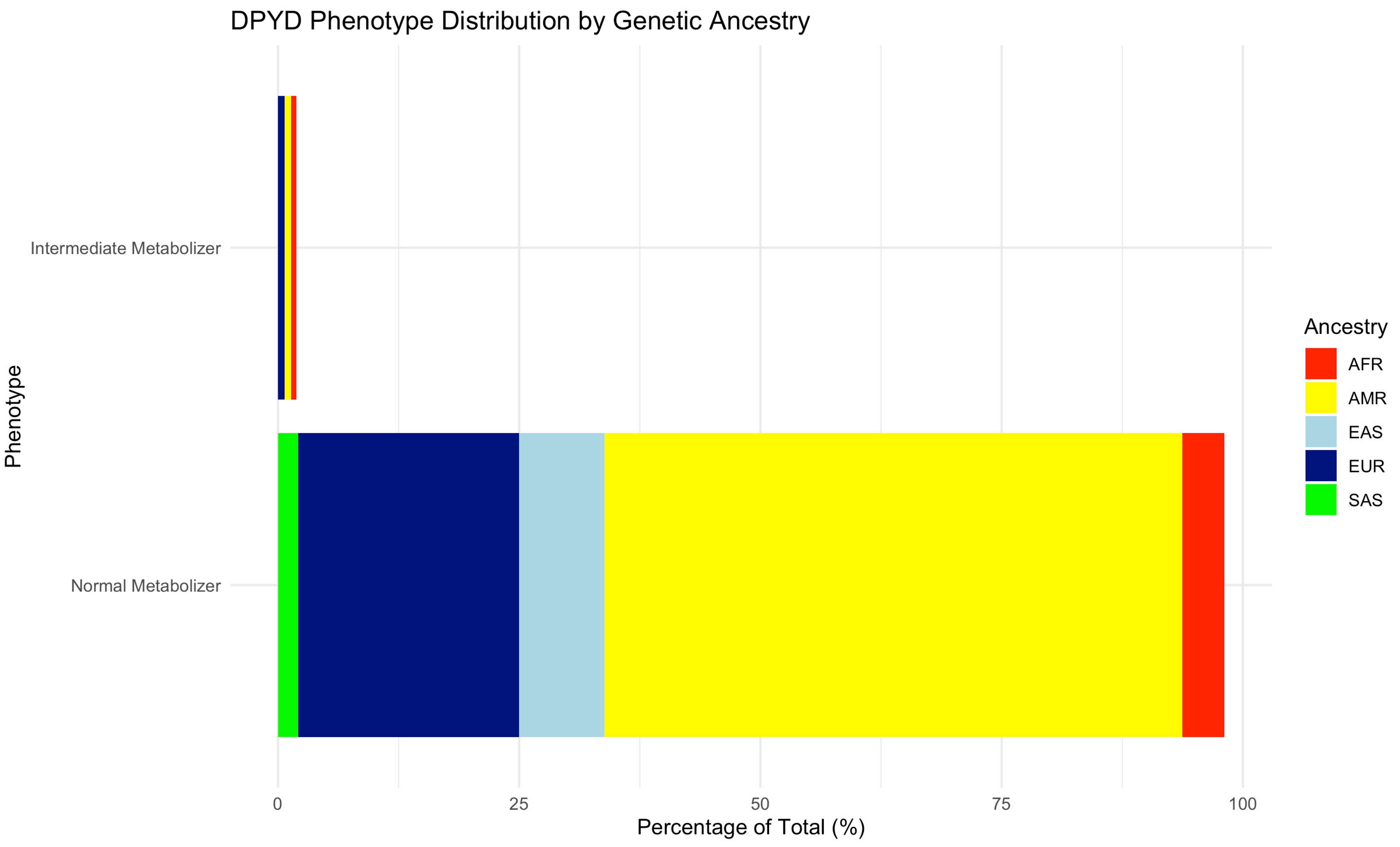
DPYD Phenotype Distribution by Genetic Ancestry
Phenotype
Intermediate Metabolizer
Normal Metabolizer
0
25
50
75
100
Percentage of Total (%)
Ancestry
AFR
AMR
EAS
EUR
SAS
