# Supplementary material for: Clinical impact of pharmacogenomics in pediatric care: insights extracted from clinical exome sequencing
Source: Front Genet. 2025 May 29;16:1574325. doi: 10.3389/fgene.2025.1574325 (PMC12159002; doi:10.3389/fgene.2025.1574325)
Supplement: Supplementary file 2 [file DataSheet2.pdf]

The Illumina DRAGEN pipeline is a command-line tool that allows users to extend its functionality by specifying various flags in conjunction with the main command. An example command to run an exome analysis, which contains steps of reads map/align, variant calling, target region coverage calculation, annotation, pharmacogenomic variants calling, and HLA variants calling is illustrated below:

```
dragen \ #Dragen main command
-r /path/to/dragen/reference \ #path where Dragen reference genome locates on the machine
-1 /path/to/read1.fastq.gz \ #path where first read of a paired-end library locates
-2 /path/to/read2.fastq.gz \ #path where second read of a paired-end library locates
--output-directory /path/to/output/dir \ #path where Dragen writes output files to
--output-file-prefix sample_001_research_pipeline \ #prefix for all output files
--RGID sample_001 \ #read group ID
--RGSM sample_001 \ #read group sample name
--enable-map-align true \ #enables the mapper/aligner
--enable-map-align-output true \ # enables saving the output (BAM) from the map/align stage
--enable-bam-indexing true \ #enables generation of a BAI index file
--enable-sort true \ #enables sorting after mapping/alignment
--enable-duplicate-marking \ #enables the flagging of duplicate output alignment records
--enable-variant-caller true \ #enables the variant caller
--vc-emit-ref-confidence GVCF \#enables generation of a GVCF file
--vc-enable-vcf-output true \#enables saving of a VCF file during a GVCF run
--enable-pgx true \ #enables Star allele caller, targeted callers for CYP2D6 and CYP2B6, and targeted caller for HLA at the same time
```

- {
- "dragenVersion": "#",
- "sample": "#",
- "pharmcatMetabolismStatusResourceUrl": "https://github.com/PharmGKB/PharmCAT/blob/aeecfe5f787e95dfb31ede62884e287affef45b3/src/main/resources/org/pharmgkb/pharmcat/definition/gene_phenotypes.json",
- "star_allele": {
- "calls": [
- {
- "gene": "DPYD",
- "lastUpdate": "10/13/2021",
- "alleleDefinitionsUrl": "https://www.pharmgkb.org/page/dpydRefMaterials",
- "genotype": "Reference/Reference",
- "pharmcatDescription": "null",
- "pharmcatMetabolismStatus": "Normal Metabolizer",
- "variants": "",
- "variantStarAllelesFound": "",
- "minGQ": "0",
- "missingGenotypes": "chr1:97579893",
- "filteredGenotypes": ""
- },
- {
- "gene": "F5",
- "lastUpdate": "-",
- "alleleDefinitionsUrl": "https://www.pharmgkb.org/gene/PA159/variantAnnotation",
- "genotype": "rs6025reference(C)/rs6025reference(C)",
- "pharmcatDescription": null,
- "pharmcatMetabolismStatus": null,
- "variants": "",
- "variantStarAllelesFound": "",
- "minGQ": "81",
- "missingGenotypes": "",
- "filteredGenotypes": ""
- },
- {
- "gene": "CACNA1S",
- "lastUpdate": "10/08/2019",
- "alleleDefinitionsUrl": "https://www.pharmgkb.org/page/cacna1sRefMaterials",
- "genotype": "Reference/Reference",
- "pharmcatDescription": "null",
- "pharmcatMetabolismStatus": "Uncertain Susceptibility",

- "variants": "",
- "variantStarAllelesFound": "",
- "minGQ": "81",
- "missingGenotypes": "",
- "filteredGenotypes": ""
- },
- {
- "gene": "UGT1A1",
- "lastUpdate": "09/24/2021",
- "alleleDefinitionsUrl": "https://www.pharmgkb.org/page/ugt1a1RefMaterials",
- "genotype": "*1/*1",
- "pharmcatDescription": "null",
- "pharmcatMetabolismStatus": "Normal Metabolizer",
- "variants": "",
- "variantStarAllelesFound": "",
- "minGQ": "0",
- "missingGenotypes": "chr2:233759924",
- "filteredGenotypes": ""
- },
- {
- "gene": "ABCG2",
- "lastUpdate": "03/01/2022",
- "alleleDefinitionsUrl": "https://www.pharmgkb.org/page/abcg2RefMaterials",
- "genotype": "rs2231142reference(G)/rs2231142reference(G)",
- "pharmcatDescription": "null",
- "pharmcatMetabolismStatus": "Normal Function",
- "variants": "",
- "variantStarAllelesFound": "",
- "minGQ": "80",
- "missingGenotypes": "",
- "filteredGenotypes": ""
- },
- {
- "gene": "TPMT",
- "lastUpdate": "09/29/2021",
- "alleleDefinitionsUrl": "https://www.pharmgkb.org/page/tpmtRefMaterials",
- "genotype": "*1/*1",
- "pharmcatDescription": "null",
- "pharmcatMetabolismStatus": "Normal Metabolizer",
- "variants": "",
- "variantStarAllelesFound": "",

- "minGQ": "41",
- "missingGenotypes": "",
- "filteredGenotypes": ""
- },
- {
- "gene": "CYP3A5",
- "lastUpdate": "09/02/2021",
- "alleleDefinitionsUrl": "https://www.pharmgkb.org/page/cyp3a5RefMaterials",
- "genotype": "*3/*1",
- "pharmcatDescription": "null",
- "pharmcatMetabolismStatus": "Intermediate Metabolizer",
- "variants": "chr7:99672916:T:C,<NON_REF>:0/1:39:14:PASS",
- "variantStarAllelesFound": "*3",
- "minGQ": "39",
- "missingGenotypes": "",
- "filteredGenotypes": ""
- },
- {
- "gene": "CFTR",
- "lastUpdate": "05/26/2020",
- "alleleDefinitionsUrl": "https://www.pharmgkb.org/page/cftrRefMaterials",
- "genotype": "ivacaftornon-responsiveCFTRsequence/ivacaftornon-responsiveCFTRsequence",
- "pharmcatDescription": "null",
- "pharmcatMetabolismStatus": "ivacaftor non-responsive in CF patients",
- "variants": "",
- "variantStarAllelesFound": "",
- "minGQ": "0",
- "missingGenotypes": "chr7:117639961",
- "filteredGenotypes": ""
- },
- {
- "gene": "NAT2",
- "lastUpdate": "4/19/16",
- "alleleDefinitionsUrl": "https://api.pharmgkb.org/v1/download/submission/1447964753",
- "genotype": "*7B/*7B",
- "pharmcatDescription": null,
- "pharmcatMetabolismStatus": null,
- "variants": "chr8:18400860:G:A,<NON_REF>:1/1:54:30:PASS;chr8:18400285:C:T,<NON_REF>:1/1:53:127:PASS",

- "variantStarAllelesFound": "*13:*13A:*7:*7A:*7B",
- "minGQ": "53",
- "missingGenotypes": "",
- "filteredGenotypes": ""
- },
- {
- "gene": "CYP2C19",
- "lastUpdate": "05/06/2021",
- "alleleDefinitionsUrl": "https://www.pharmgkb.org/page/cyp2c19RefMaterials",
- "genotype": "*1/*2",
- "pharmcatDescription": "null",
- "pharmcatMetabolismStatus": "Intermediate Metabolizer",
- "variants": "chr10:94781859:G:A,<NON_REF>:0/1:30:177:PASS;chr10:94775367:A:G,<NON_REF>:0/1:27:221:PASS;chr10:94842866:A:G,<NON_REF>:1/1:46:294:PASS",
- "variantStarAllelesFound": "*1:*2:*35",
- "minGQ": "0",
- "missingGenotypes": "chr10:94761900",
- "filteredGenotypes": ""
- },
- {
- "gene": "CYP2C9",
- "lastUpdate": "09/02/2021",
- "alleleDefinitionsUrl": "https://www.pharmgkb.org/page/cyp2c9RefMaterials",
- "genotype": "*1/*1",
- "pharmcatDescription": "null",
- "pharmcatMetabolismStatus": "Normal Metabolizer",
- "variants": "",
- "variantStarAllelesFound": "",
- "minGQ": "80",
- "missingGenotypes": "",
- "filteredGenotypes": ""
- },
- {
- "gene": "SLCO1B1",
- "lastUpdate": "07/12/2021",
- "alleleDefinitionsUrl": "https://www.pharmgkb.org/page/slco1b1RefMaterials",
- "genotype": "*37/*37",
- "pharmcatDescription": "null",
- "pharmcatMetabolismStatus": "Normal Function",
- "variants": "chr12:21176804:A:G,<NON_REF>:1/1:55:84:PASS",
- "variantStarAllelesFound": "*37",

- "minGQ": "11",
- "missingGenotypes": "",
- "filteredGenotypes": ""
- },
- {
- "gene": "NUDT15",
- "lastUpdate": "10/13/2021",
- "alleleDefinitionsUrl": "https://www.pharmgkb.org/page/nudt15RefMaterials",
- "genotype": "*1/*1",
- "pharmcatDescription": "null",
- "pharmcatMetabolismStatus": "Normal Metabolizer",
- "variants": "",
- "variantStarAllelesFound": "",
- "minGQ": "53",
- "missingGenotypes": "",
- "filteredGenotypes": ""
- },
- {
- "gene": "VKORC1",
- "lastUpdate": "10/08/19",
- "alleleDefinitionsUrl": "https://www.pharmgkb.org/page/vkorc1RefMaterials",
- "genotype": "rs9923231reference(C)/rs9923231reference(C)",
- "pharmcatDescription": null,
- "pharmcatMetabolismStatus": null,
- "variants": "",
- "variantStarAllelesFound": "",
- "minGQ": "0",
- "missingGenotypes": "",
- "filteredGenotypes": "chr16:31096368:C:<NON_REF>:0/0:0:5:LowGQ"
- },
- {
- "gene": "CYP4F2",
- "lastUpdate": "10/08/2019",
- "alleleDefinitionsUrl": "https://www.pharmgkb.org/page/cyp4f2RefMaterials",
- "genotype": "*3/*1",
- "pharmcatDescription": null,
- "pharmcatMetabolismStatus": null,
- "variants": "chr19:15879621:C:T,<NON_REF>:0/1:40:220:PASS",
- "variantStarAllelesFound": "*3",
- "minGQ": "40",
- "missingGenotypes": "",

- "filteredGenotypes": ""
- },
- {
- "gene": "RYR1",
- "lastUpdate": "10/09/2020",
- "alleleDefinitionsUrl": "https://www.pharmgkb.org/page/ryr1RefMaterials",
- "genotype": "Reference/Reference",
- "pharmcatDescription": "null",
- "pharmcatMetabolismStatus": "Uncertain Susceptibility",
- "variants": "",
- "variantStarAllelesFound": "",
- "minGQ": "23",
- "missingGenotypes": "",
- "filteredGenotypes": ""
- },
- {
- "gene": "IFNL3",
- "lastUpdate": "10/08/19",
- "alleleDefinitionsUrl": "https://www.pharmgkb.org/page/ifnl3RefMaterials",
- "genotype": "rs12979860reference(C)/rs12979860reference(C)",
- "pharmcatDescription": null,
- "pharmcatMetabolismStatus": null,
- "variants": "",
- "variantStarAllelesFound": "",
- "minGQ": "9",
- "missingGenotypes": "",
- "filteredGenotypes": ""
- },
- {
- "gene": "G6PD",
- "lastUpdate": "02/25/21",
- "alleleDefinitionsUrl": "https://www.pharmgkb.org/page/g6pdRefMaterials",
- "genotype": "B(wildtype)",
- "pharmcatDescription": null,
- "pharmcatMetabolismStatus": null,
- "variants": "",
- "variantStarAllelesFound": "",
- "minGQ": "53",
- "missingGenotypes": "",
- "filteredGenotypes": ""
- },

- {
- "gene": "MT-RNR1",
- "lastUpdate": "08/16/2021",
- "alleleDefinitionsUrl": "https://www.pharmgkb.org/page/mtrnr1RefMaterials",
- "genotype": "Reference",
- "pharmcatDescription": null,
- "pharmcatMetabolismStatus": null,
- "variants": "",
- "variantStarAllelesFound": "",
- "minGQ": "99",
- "missingGenotypes": "",
- "filteredGenotypes": ""
- },
- {
- "gene": "BCHE",
- "lastUpdate": "",
- "alleleDefinitionsUrl": "https://www.dovepress.com/getfile.php?fileID=61995",
- "genotype": "ref/ref",
- "pharmcatDescription": null,
- "pharmcatMetabolismStatus": null,
- "variants": "",
- "variantStarAllelesFound": "",
- "minGQ": "81",
- "missingGenotypes": "",
- "filteredGenotypes": ""
- },
- {
- "gene": "UGT2B17",
- "lastUpdate": "",
- "alleleDefinitionsUrl": "https://www.pharmacogenomics.pha.ulaval.ca/wp-content/uploads/2015/04/HAP-UGT2B17.htm",
- "genotype": "*1/*1",
- "pharmcatDescription": null,
- "pharmcatMetabolismStatus": null,
- "variants": "",
- "variantStarAllelesFound": "",
- "minGQ": "0",
- "missingGenotypes": "",
- "filteredGenotypes": ""
- }
-]
- },

- "cyp2d6": {
- "genotype": "*13+*2/*2",
- "genotypeFilter": "PASS",
- "pharmcatDescription": null,
- "pharmcatMetabolismStatus": null
- },
- "cyp2b6": {
- "genotype": "*30/*6",
- "genotypeFilter": "PASS",
- "pharmcatDescription": "null",
- "pharmcatMetabolismStatus": "Poor Metabolizer"
- },
- "hla": {
- "calls": [
- {
- "gene": "HLA-A",
- "genotype": "*30:01/*26:01"
- },
- {
- "gene": "HLA-B",
- "genotype": "*07:02/*13:02"
- },
- {
- "gene": "HLA-C",
- "genotype": "*07:02/*06:02"
- }
-]
- }

}
