# Supplementary material for: Clinical impact of pharmacogenomics in pediatric care: insights extracted from clinical exome sequencing
Source: Front Genet. 2025 May 29;16:1574325. doi: 10.3389/fgene.2025.1574325 (PMC12159002; doi:10.3389/fgene.2025.1574325)
Supplement: Supplementary file 4 [file DataSheet4.pdf]

```
import os
import json

def generate_report(data):
    # Extract sample ID
    sample_id = data.get('sample', '')

    # Create the report header and table header
    report = f"Pharmacogenomic report generated by Dragen V4.2 using
PHARMCAT\nSample ID: {sample_id}\n\n"
    table_header = "Gene
Name\tGenotype\tpharmcatMetabolismStatus\tvariantStarAllelesFound\tv
ariants\tmissingGenotypes\n"

    # Initialize the table rows
    table_rows = []

    # Process the "star_allele" data
    star_allele_calls = data.get('star_allele', {}).get('calls', [])
    for call in star_allele_calls:
        gene_name = call.get('gene', '')
        genotype = call.get('genotype', '')
        metabolism_status = call.get('pharmcatMetabolismStatus', '')
        variant_star_alleles_found =
call.get('variantStarAllelesFound', '')
        variants = call.get('variants', '')
        missing_genotypes = call.get('missingGenotypes', '')

        table_row = f"{gene_name}\t{genotype}\t{metabolism_status}
\t{variant_star_alleles_found}\t{variants}\t{missing_genotypes}"
        table_rows.append(table_row)

    # Process the "cyp2d6" data
    cyp2d6_genotype = data.get('cyp2d6', {}).get('genotype', '')
    cyp2d6_metabolism_status = data.get('cyp2d6',
{}).get('pharmcatMetabolismStatus', '')
    cyp2d6_row = f"cyp2d6\t{cyp2d6_genotype}
\t{cyp2d6_metabolism_status}\t\t\t"
    table_rows.append(cyp2d6_row)

    # Process the "cyp2b6" data
    cyp2b6_genotype = data.get('cyp2b6', {}).get('genotype', '')
    cyp2b6_metabolism_status = data.get('cyp2b6',
{}).get('pharmcatMetabolismStatus', '')
    cyp2b6_row = f"cyp2b6\t{cyp2b6_genotype}
\t{cyp2b6_metabolism_status}\t\t\t"
    table_rows.append(cyp2b6_row)

    # Process the "hla" data
    hla_calls = data.get('hla', {}).get('calls', [])
    for call in hla_calls:
        gene_name = call.get('gene', '')
        genotype = call.get('genotype', '')
```

```
        table_row = f"{gene_name}\t{genotype}\t\t\t\t"
        table_rows.append(table_row)

    # Combine the header and table rows
    report += table_header + '\n'.join(table_rows)
    return report

def process_json_files(input_directory, output_directory):
    for filename in os.listdir(input_directory):
        if filename.endswith('.json'):
            json_path = os.path.join(input_directory, filename)
            with open(json_path, 'r') as json_file:
                data = json.load(json_file)

            report = generate_report(data)

            # Output report to a corresponding text file in the
output directory
            report_filename = filename.replace('.json',
'_report.txt')
            report_path = os.path.join(output_directory,
report_filename)
            with open(report_path, 'w') as report_file:
                report_file.write(report)

if __name__ == "__main__":
    input_directory = '#'  # Replace with your input directory path
    output_directory = '#'  # Replace with your output directory
path
    process_json_files(input_directory, output_directory)
```
