# Supplementary figures and images for: Clinical impact of pharmacogenomics in pediatric care: insights extracted from clinical exome sequencing
Source: Front Genet. 2025 May 29;16:1574325. doi: 10.3389/fgene.2025.1574325 (PMC12159002; doi:10.3389/fgene.2025.1574325)

Average Coverage for CYP3A5

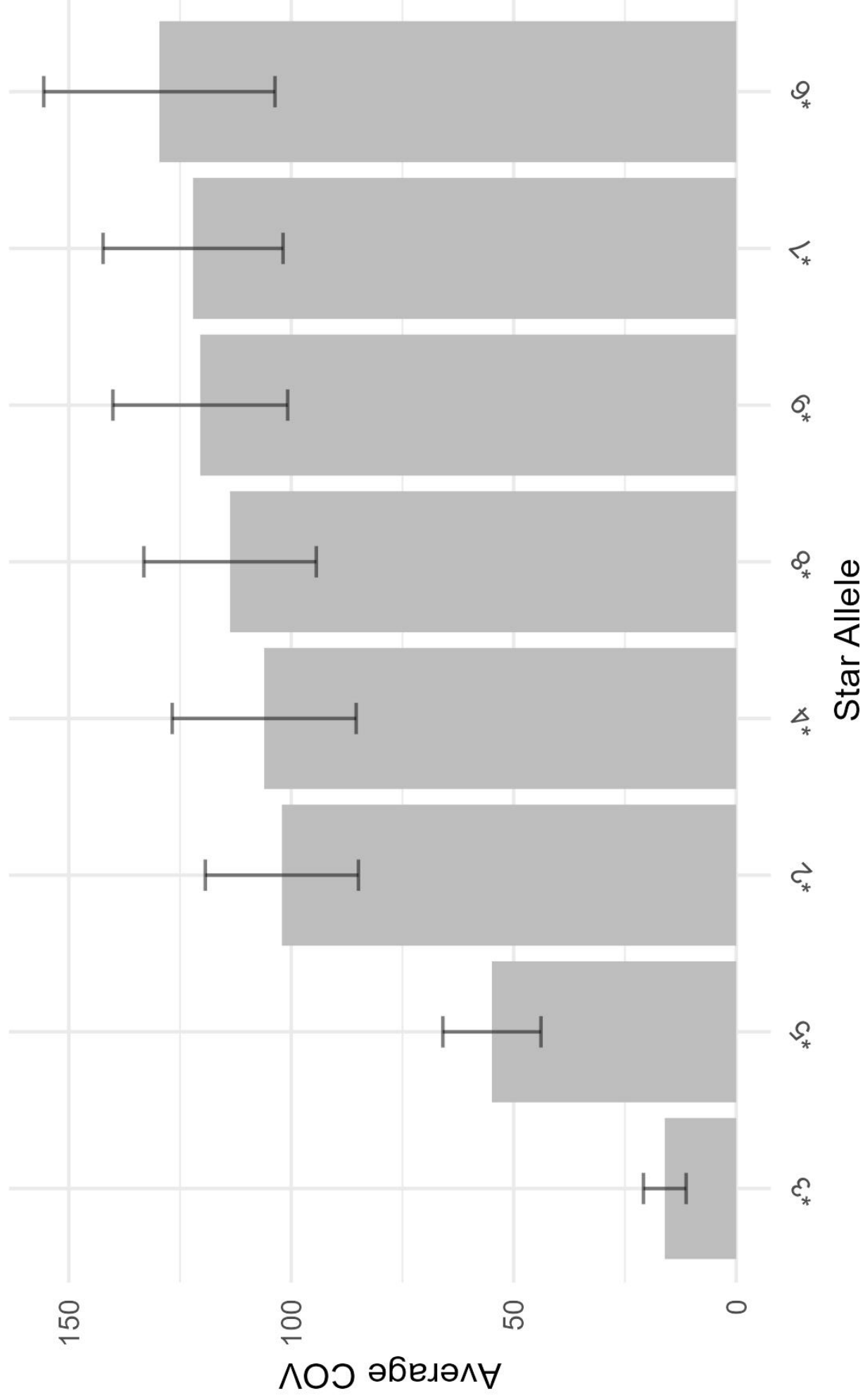

Supplement: Supplementary file 5 [file DataSheet6.pdf]

a

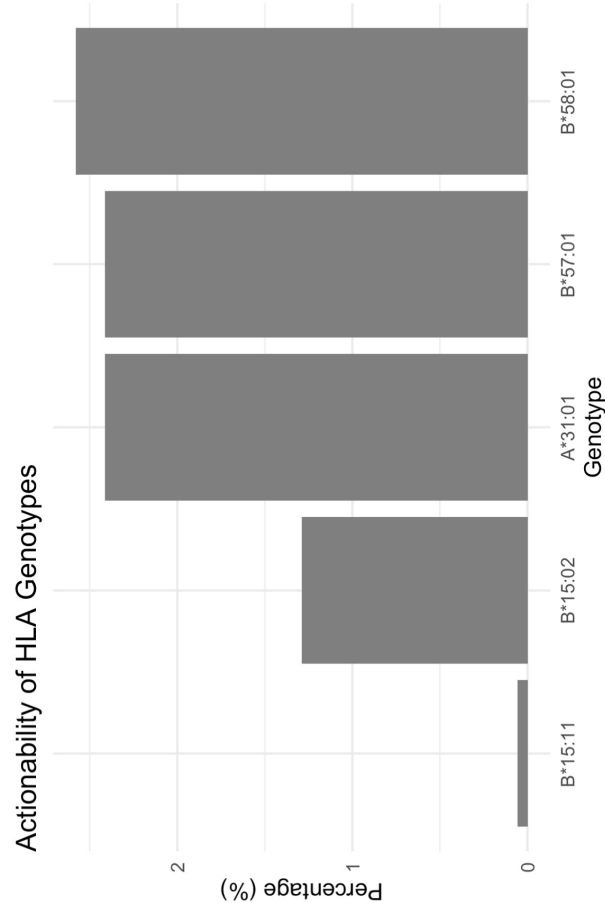

b

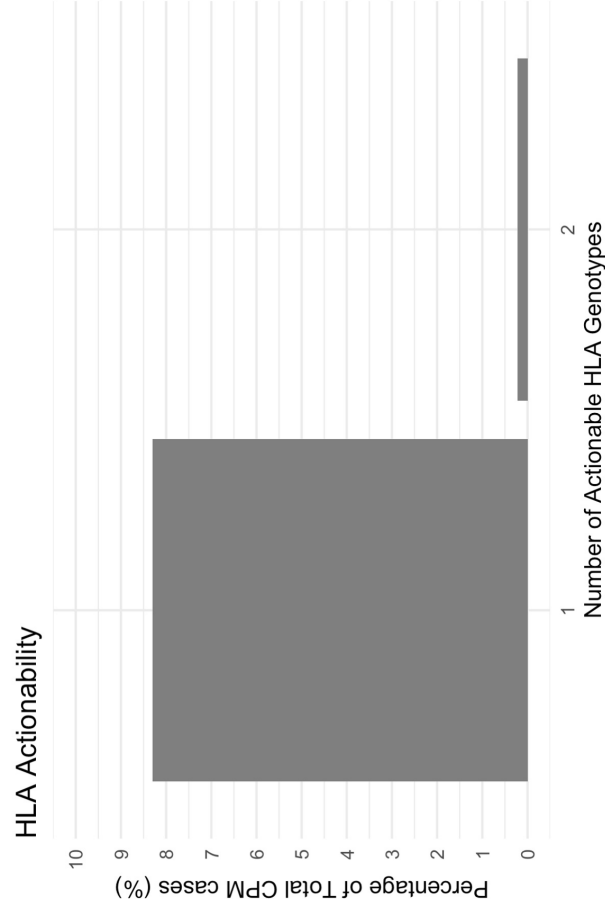

HLA Actionable Medication by Genetic Ancestry

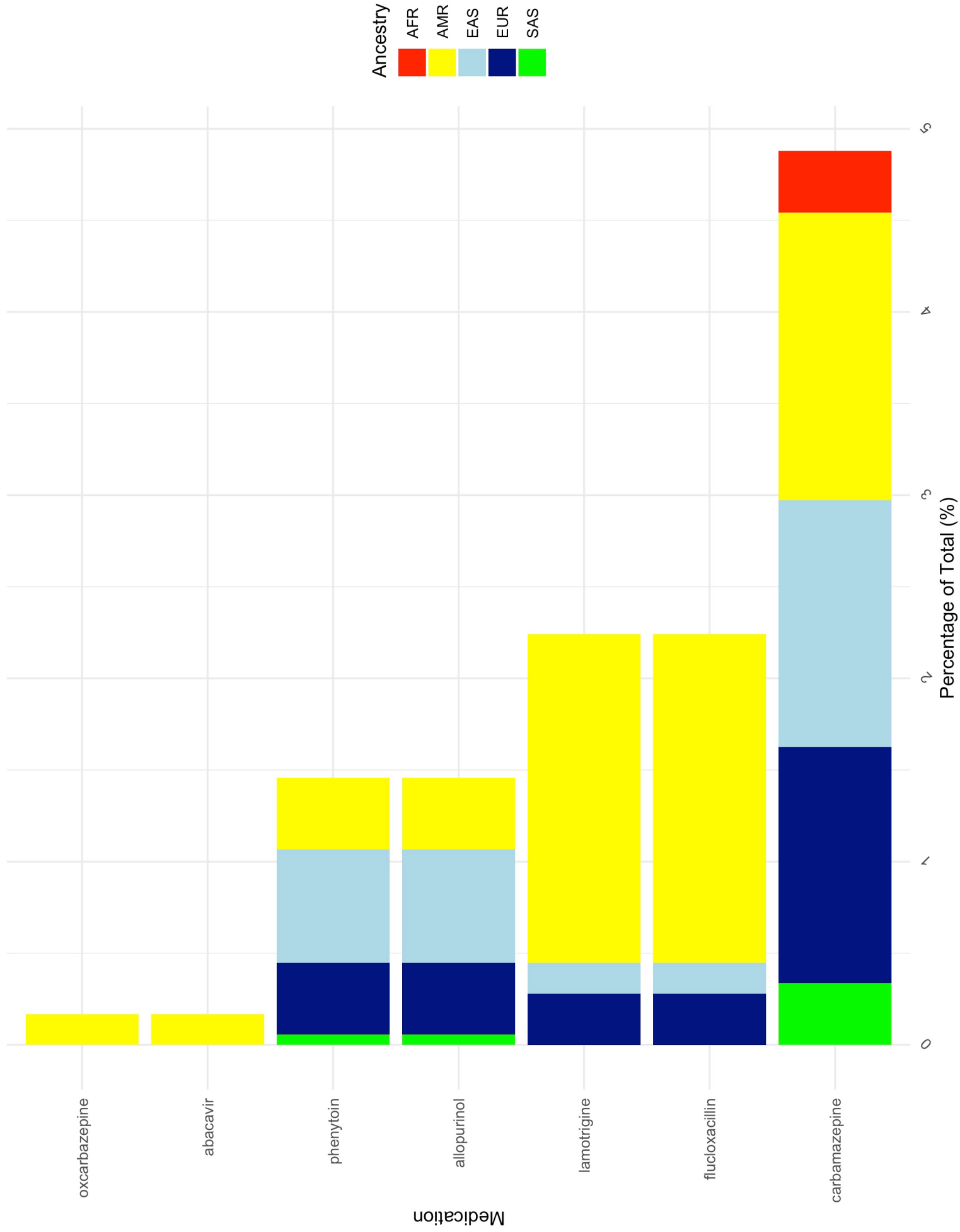

Supplement: Supplementary file 8 [file DataSheet9.pdf]
