# Supplementary material for: Clinical impact of pharmacogenomics in pediatric care: insights extracted from clinical exome sequencing
Source: Front Genet. 2025 May 29;16:1574325. doi: 10.3389/fgene.2025.1574325 (PMC12159002; doi:10.3389/fgene.2025.1574325)
Supplement: Supplementary file 9 [file DataSheet5.pdf]

Average Coverage for G6PD

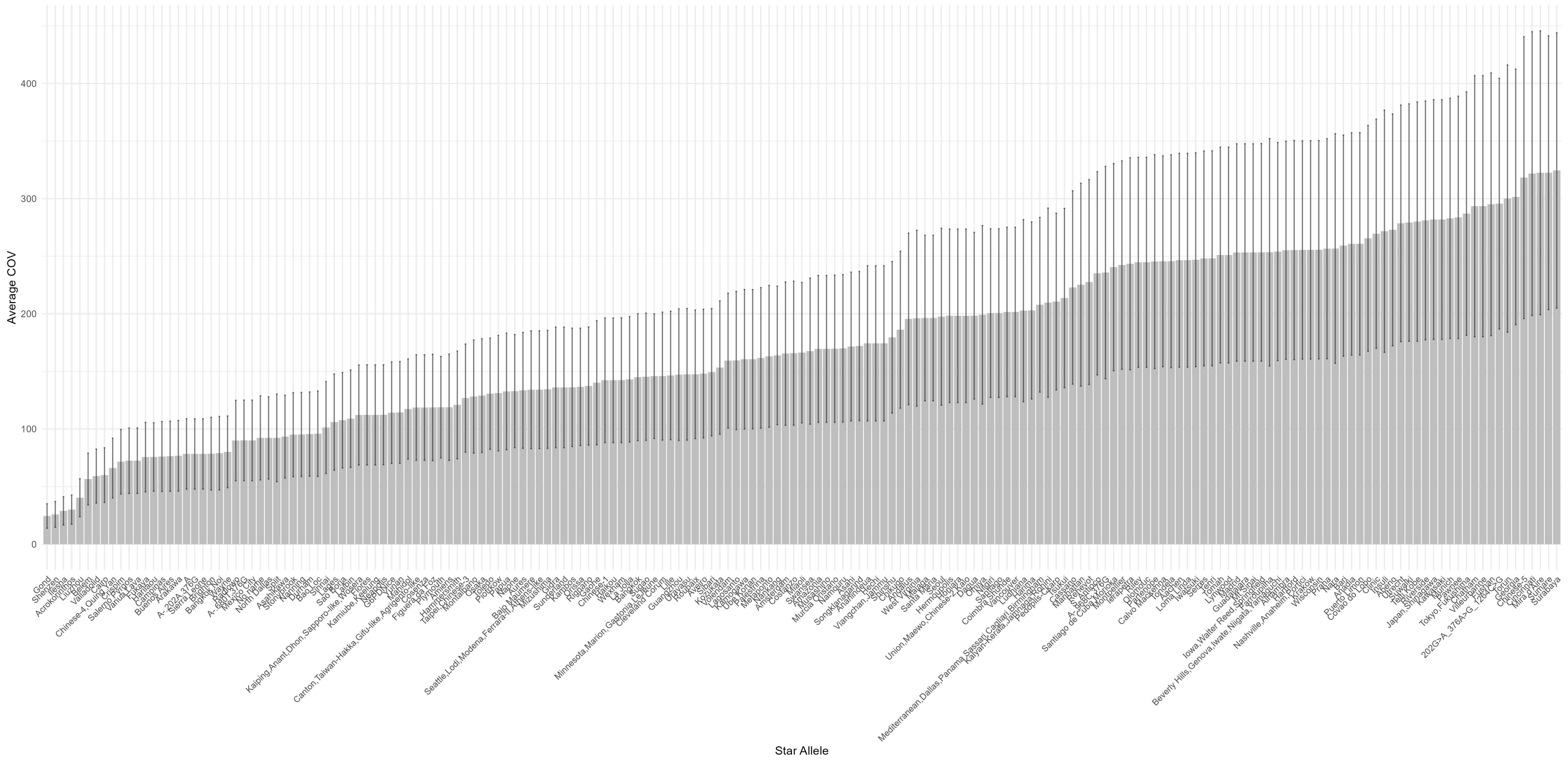

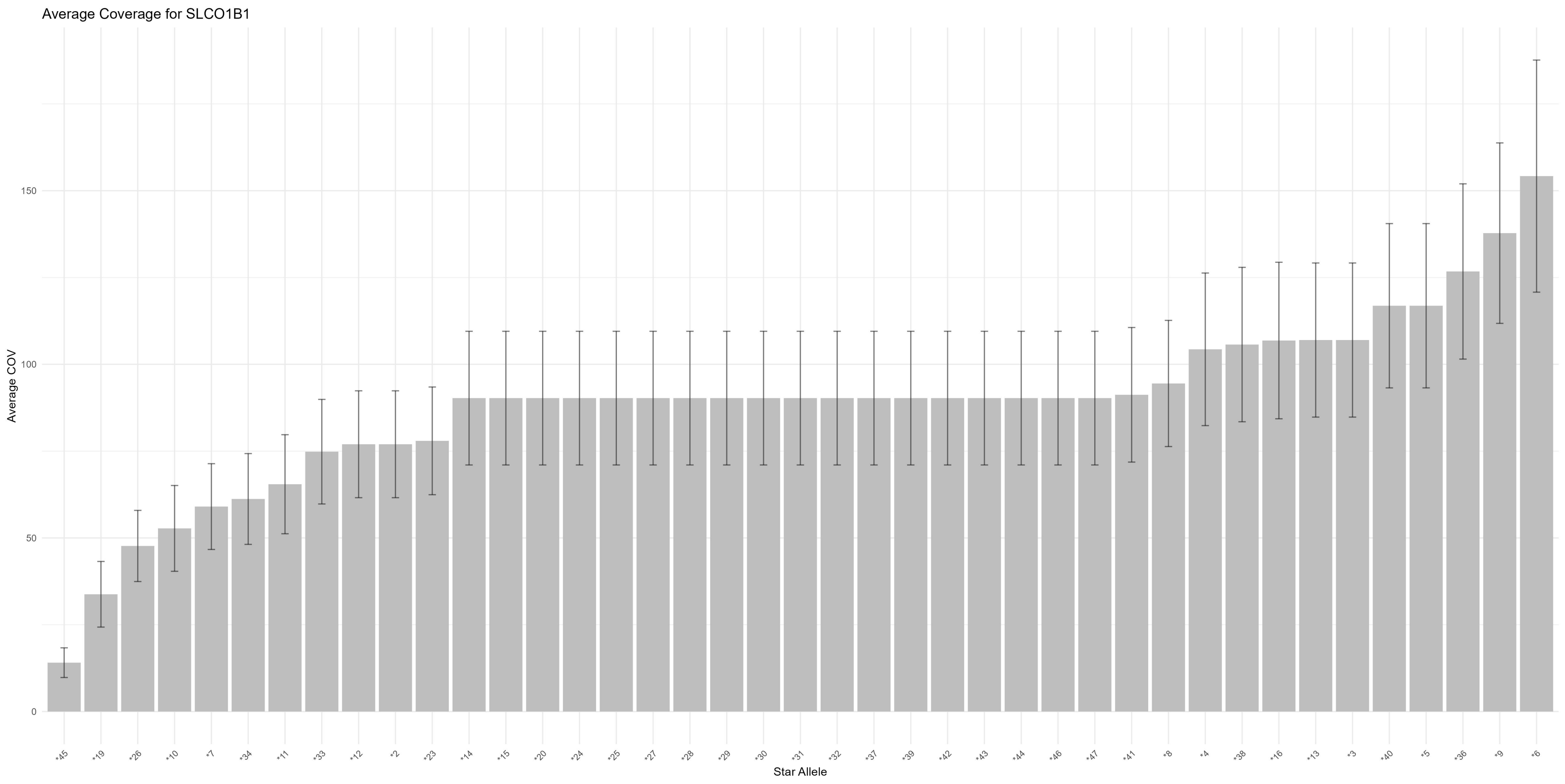

Average Coverage for SLCO1B1
Average COV
0
50
100
150
Star Allele
*45
*19
*26
*10
*7
*34
*11
*33
*12
*2
*23
*14
*15
*20
*24
*25
*27
*28
*29
*30
*31
*32
*37
*39
*42
*43
*44
*46
*47
*41
*8
*4
*38
*16
*13
*3
*40
*5
*36
*9
*6

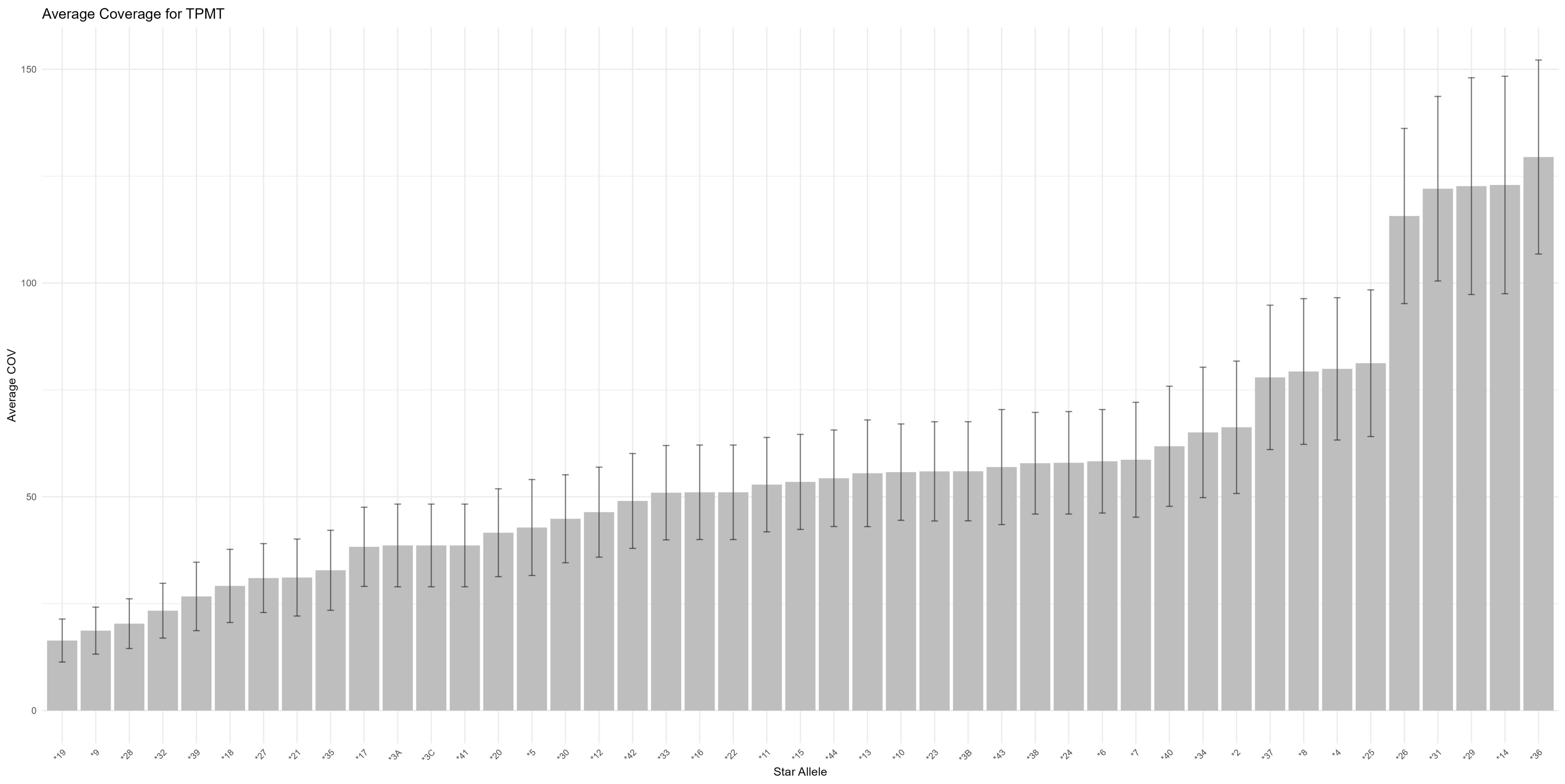
Average Coverage for TPMT
Average COV
150
100
50
0
*19
*9
*28
*32
*39
*18
*27
*21
*35
*17
*3A
*3C
*41
*20
*5
*30
*12
*42
*33
*16
*22
*11
*15
*44
*13
*10
*23
*3B
*43
*38
*24
*6
*7
*40
*34
*2
*37
*8
*4
*25
*26
*31
*29
*14
*36
Star Allele

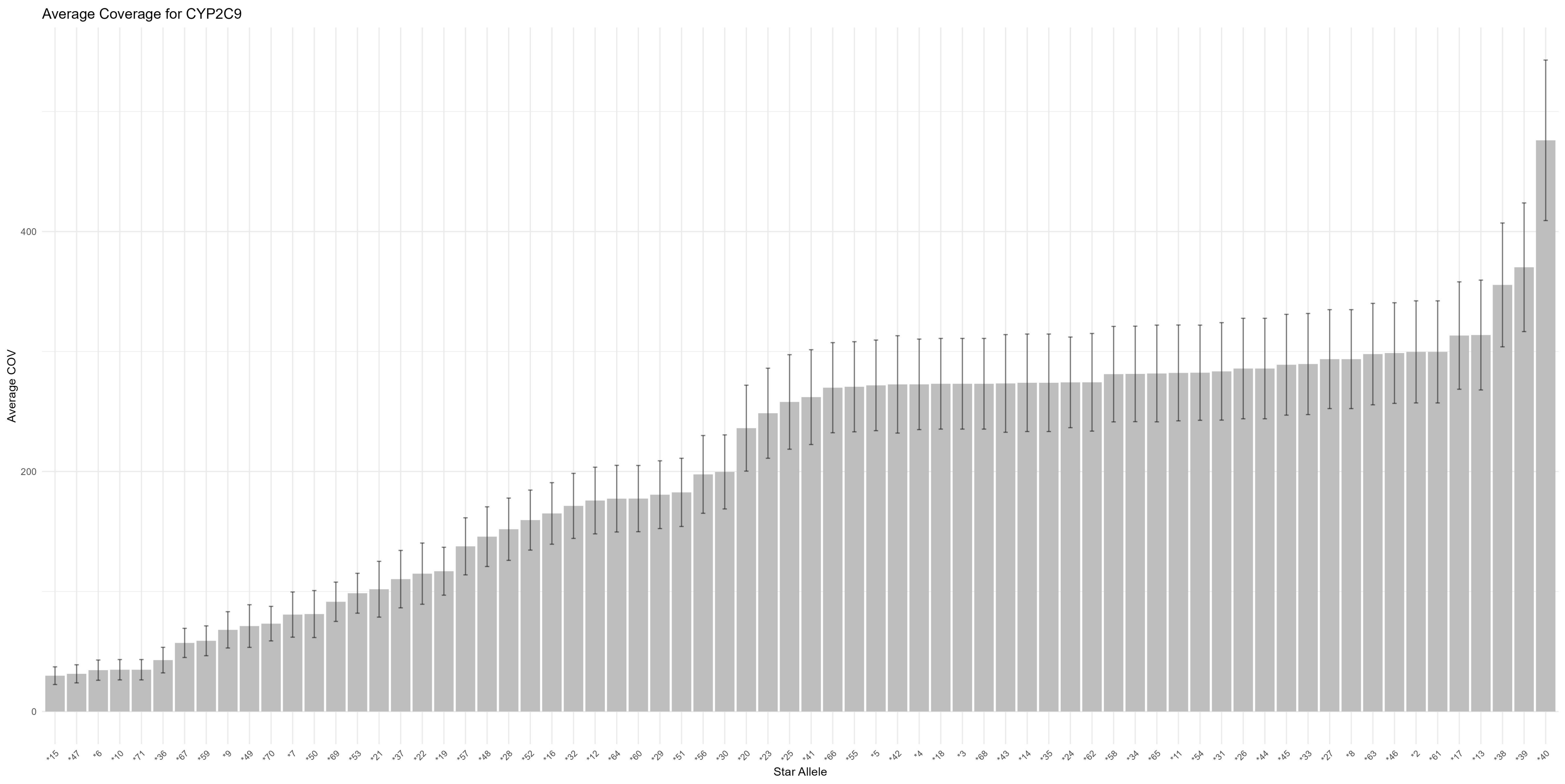
Average Coverage for CYP2C9
Average COV
400
200
0
*15 *47 *6 *10 *71 *36 *67 *59 *9 *49 *70 *7 *50 *69 *53 *21 *37 *22 *19 *57 *48 *28 *52 *16 *32 *12 *64 *60 *29 *51 *56 *30 *20 *23 *25 *41 *66 *55 *5 *42 *4 *18 *3 *68 *43 *14 *35 *24 *62 *58 *34 *65 *11 *54 *31 *26 *44 *45 *33 *27 *8 *63 *46 *2 *61 *17 *13 *38 *39 *40
Star Allele

Average Coverage for CYP2D6

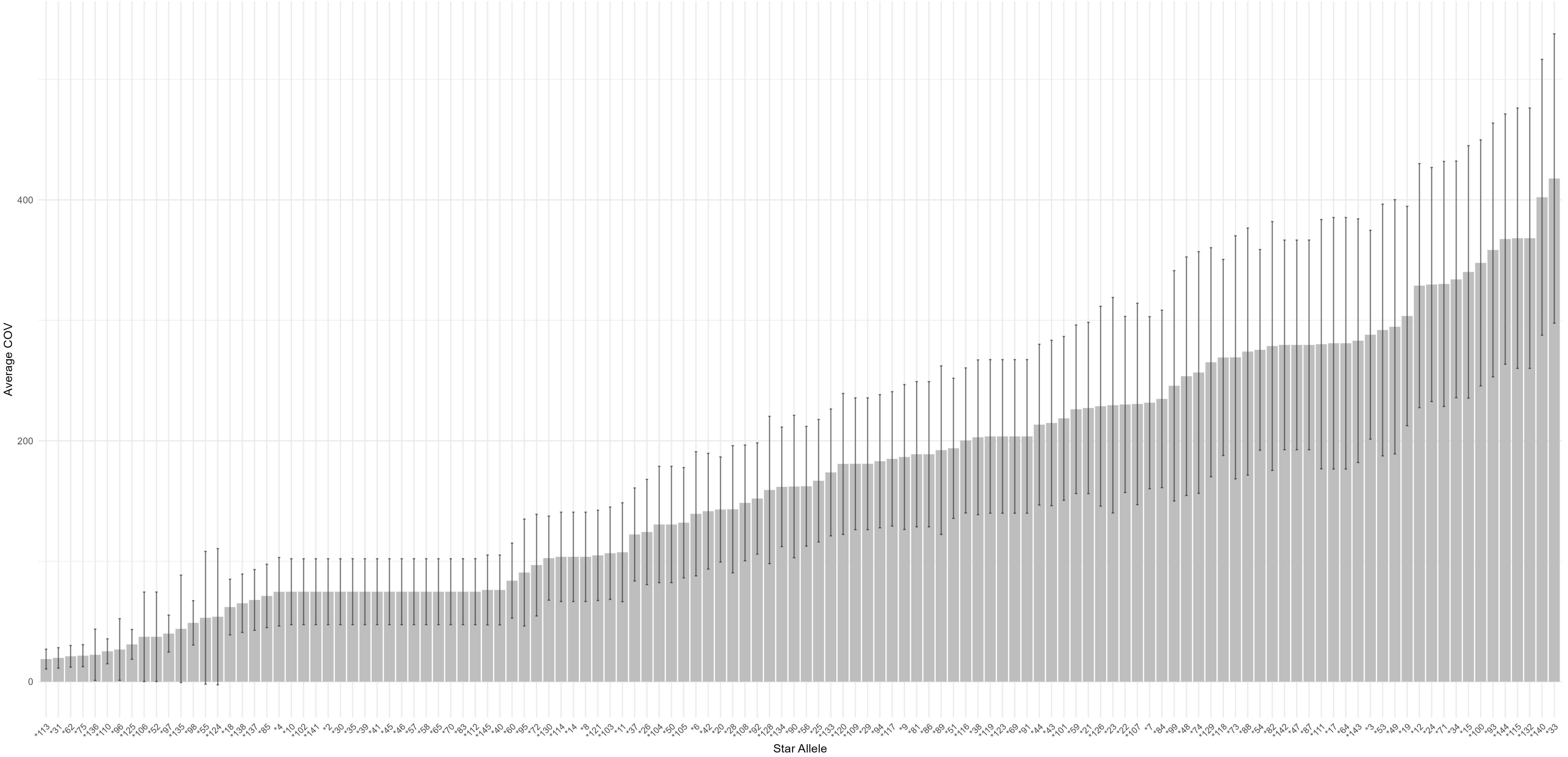

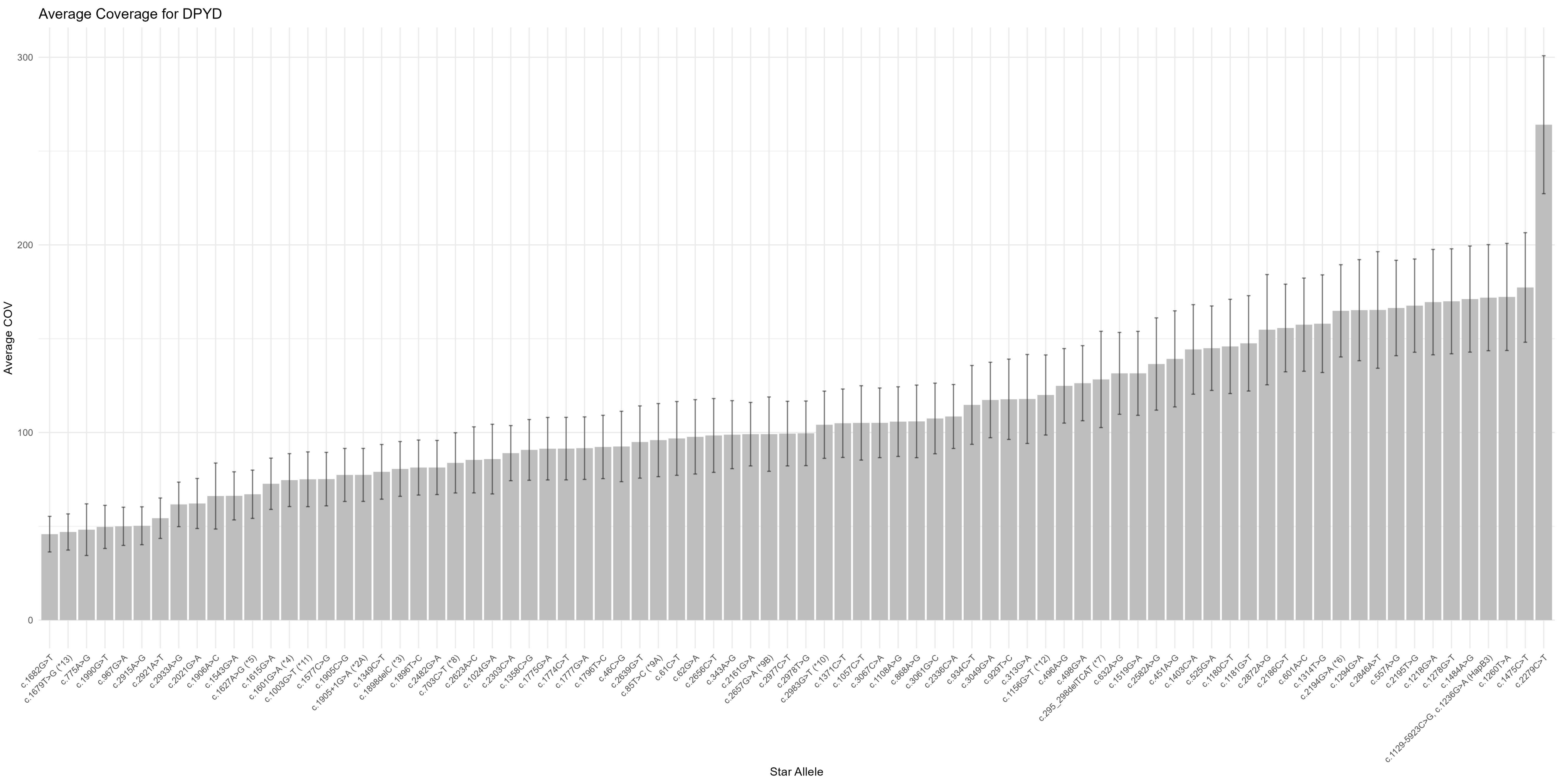

Average Coverage for DPYD
Average COV
0
100
200
300
Star Allele
c.1682G>T
c.1679T>G (*13)
c.775A>G
c.1990G>T
c.967G>A
c.2915A>G
c.2921A>T
c.2933A>G
c.2021G>A
c.1906A>C
c.1543G>A
c.1627A>G (*5)
c.1615G>A
c.1601G>A (*4)
c.1003G>T (*11)
c.1577C>G
c.1905C>G
c.1905+1G>A (*2A)
c.1349C>T
c.1898delC (*3)
c.1896T>C
c.2482G>A
c.703C>T (*8)
c.2623A>C
c.1024G>A
c.2303C>A
c.1358C>G
c.1775G>A
c.1774C>T
c.1777G>A
c.1796T>C
c.46C>G
c.2639G>T
c.85T>C (*9A)
c.61C>T
c.62G>A
c.2656C>T
c.343A>G
c.2161G>A
c.2657G>A (*9B)
c.2977C>T
c.2978T>G
c.2983G>T (*10)
c.1371C>T
c.1057C>T
c.3067C>A
c.1108A>G
c.868A>G
c.3061G>C
c.2336C>A
c.934C>T
c.3049G>A
c.929T>C
c.313G>A
c.1156G>T (*12)
c.496A>G
c.498G>A
c.295_298delTCAT (*7)
c.632A>G
c.1519G>A
c.2582A>G
c.451A>G
c.1403C>A
c.525G>A
c.1180C>T
c.1181G>T
c.2872A>G
c.2186C>T
c.601A>C
c.1314T>G
c.2194G>A (*6)
c.1294G>A
c.2846A>T
c.557A>G
c.2195T>G
c.1218G>A
c.1278G>T
c.1484A>G
c.1129-5923C>G, c.1236G>A (HapB3)
c.1260T>A
c.1475C>T
c.2279C>T

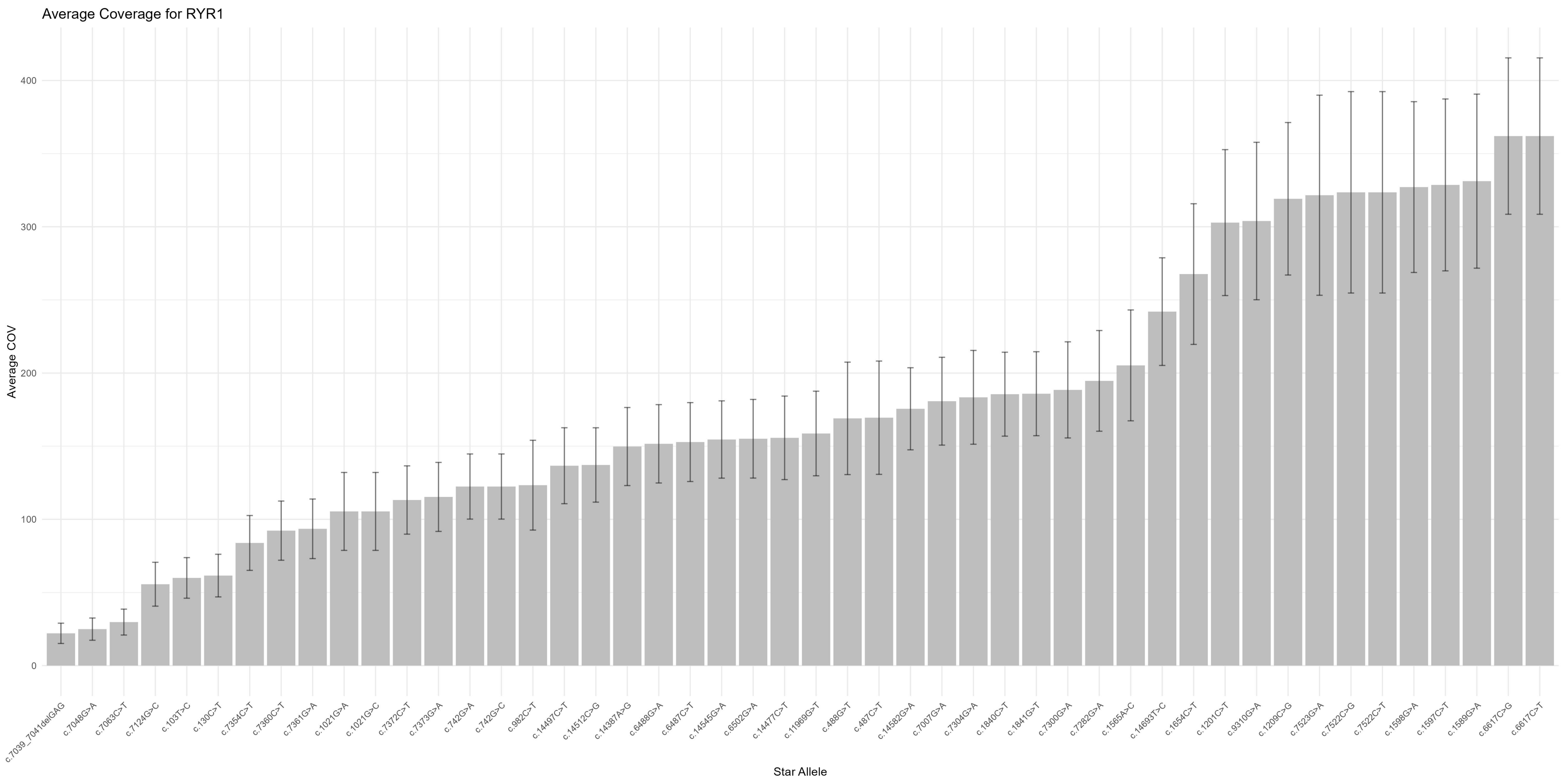

Average Coverage for RYR1
Average COV
Star Allele
0
100
200
300
400
c.7039_7041delGAG
c.7048G>A
c.7063C>T
c.7124G>C
c.103T>C
c.130C>T
c.7354C>T
c.7360C>T
c.7361G>A
c.1021G>A
c.1021G>C
c.7372C>T
c.7373G>A
c.742G>A
c.742G>C
c.982C>T
c.14497C>T
c.14512C>G
c.14387A>G
c.6488G>A
c.6487C>T
c.14545G>A
c.6502G>A
c.14477C>T
c.11969G>T
c.488G>T
c.487C>T
c.14582G>A
c.7007G>A
c.7304G>A
c.1840C>T
c.1841G>T
c.7300G>A
c.7282G>A
c.1565A>C
c.14693T>C
c.1654C>T
c.1201C>T
c.9310G>A
c.1209C>G
c.7523G>A
c.7522C>G
c.7522C>T
c.1598G>A
c.1597C>T
c.1589G>A
c.6617C>G
c.6617C>T

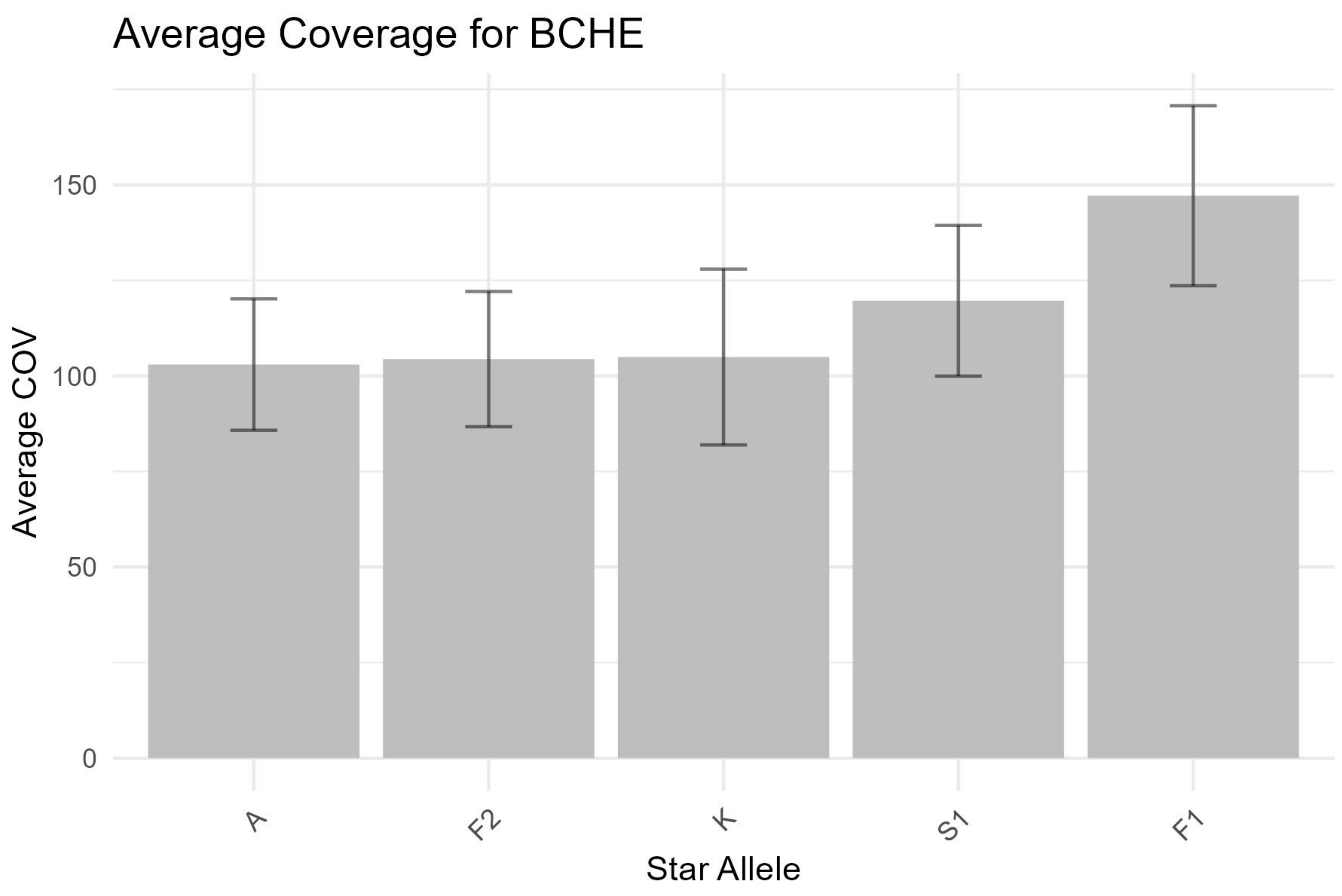
Average Coverage for BCHE
Average COV
150
100
50
0
A
F2
K
S1
F1
Star Allele

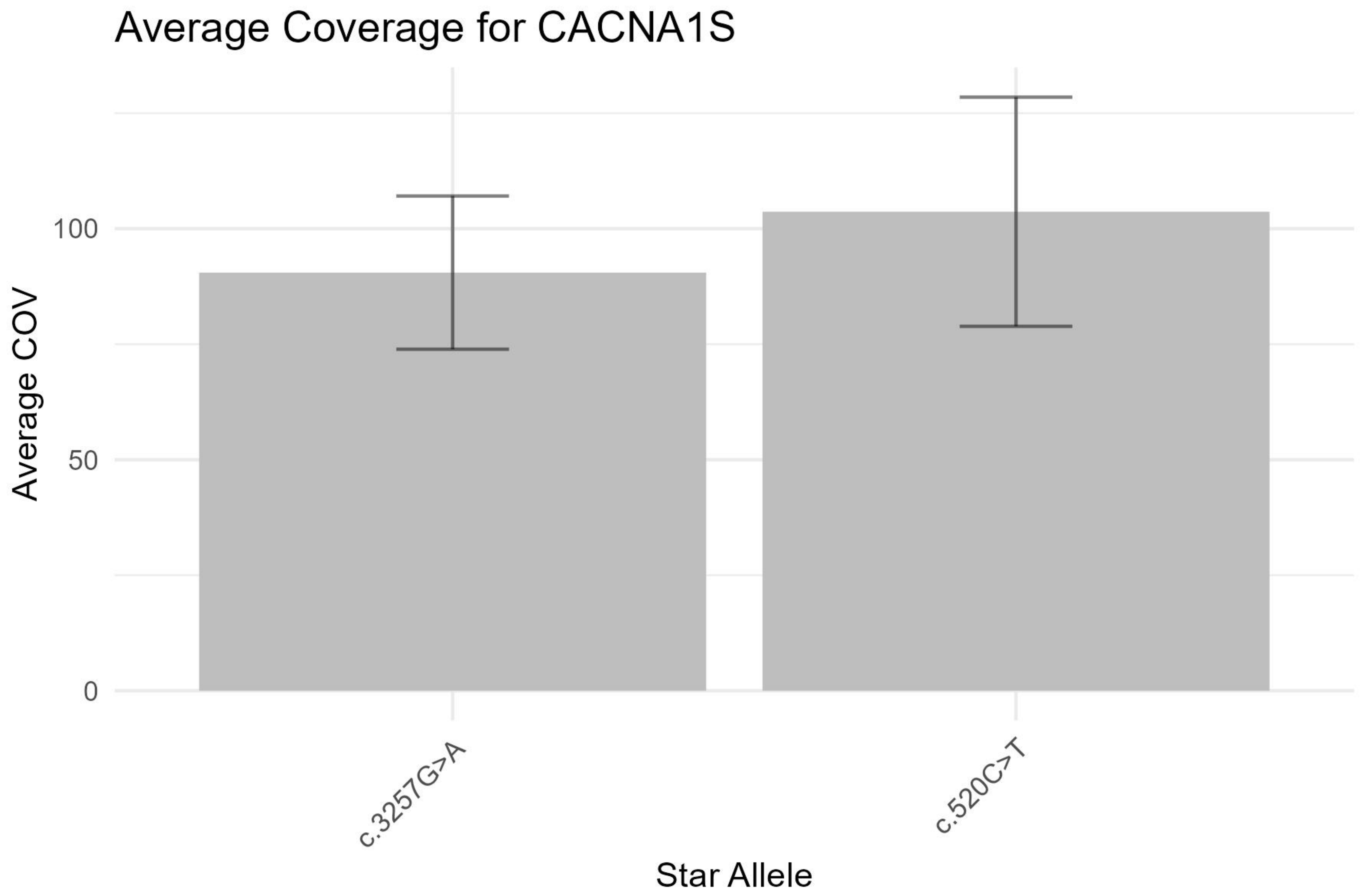
Average Coverage for CACNA1S
Average COV
100
50
0
c.3257G>A
c.520C>T
Star Allele

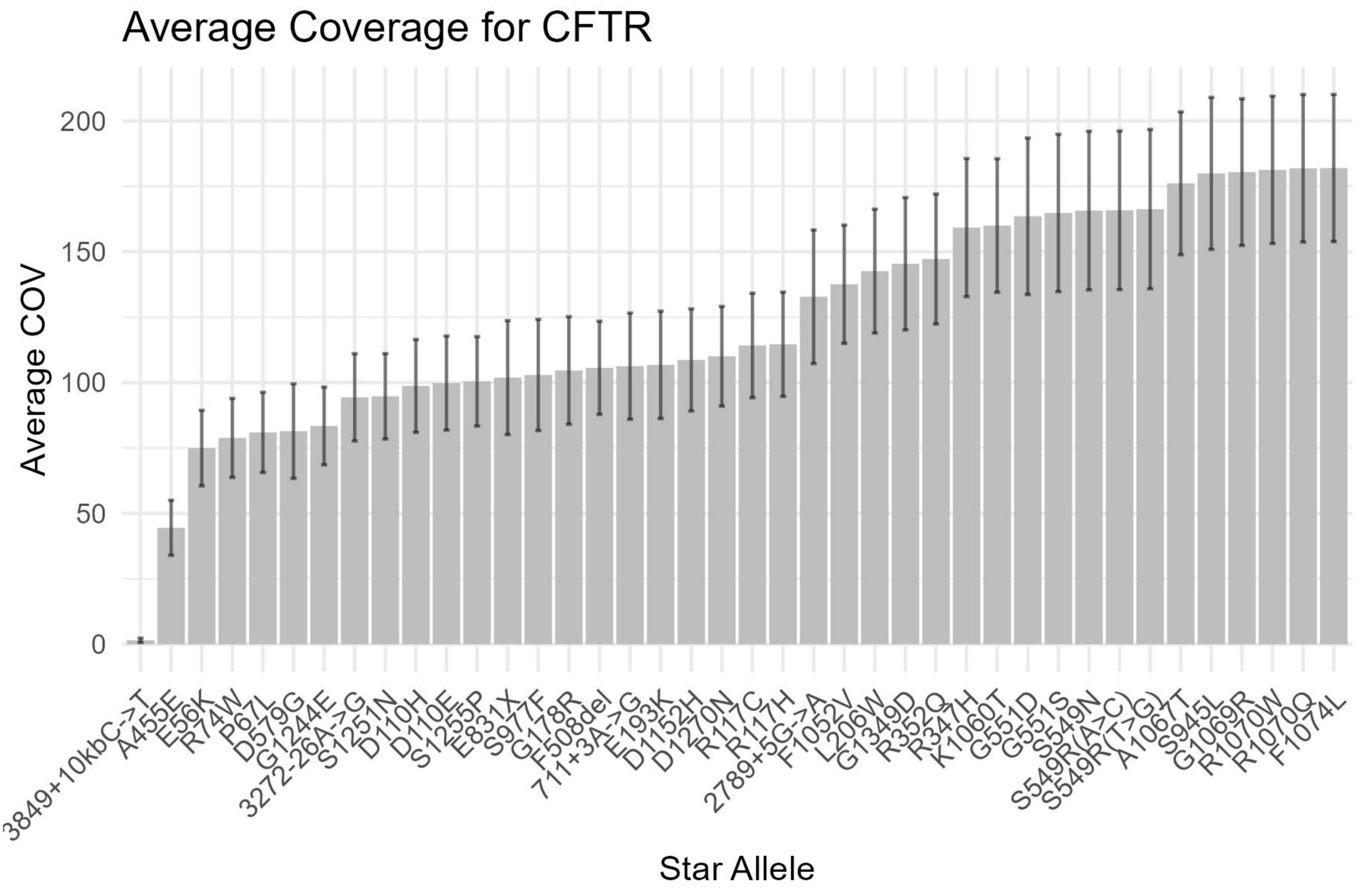

Average Coverage for CFTR
Average COV
0
50
100
150
200
3849+10kbC->T
A455E
E56K
R74W
P67L
D579G
G1244E
3272-26A->G
S1251N
D110H
D110E
S1255P
E831X
S977F
G178R
F508del
711+3A->G
E193K
D1152H
D1270N
R117C
R117H
2789+5G->A
F1052V
L206W
G1349D
R352Q
R347H
K1060T
G551D
G551S
S549N
S549R(A>C)
S549R(T>G)
A1067T
S945L
G1069R
R1070W
R1070Q
F1074L
Star Allele

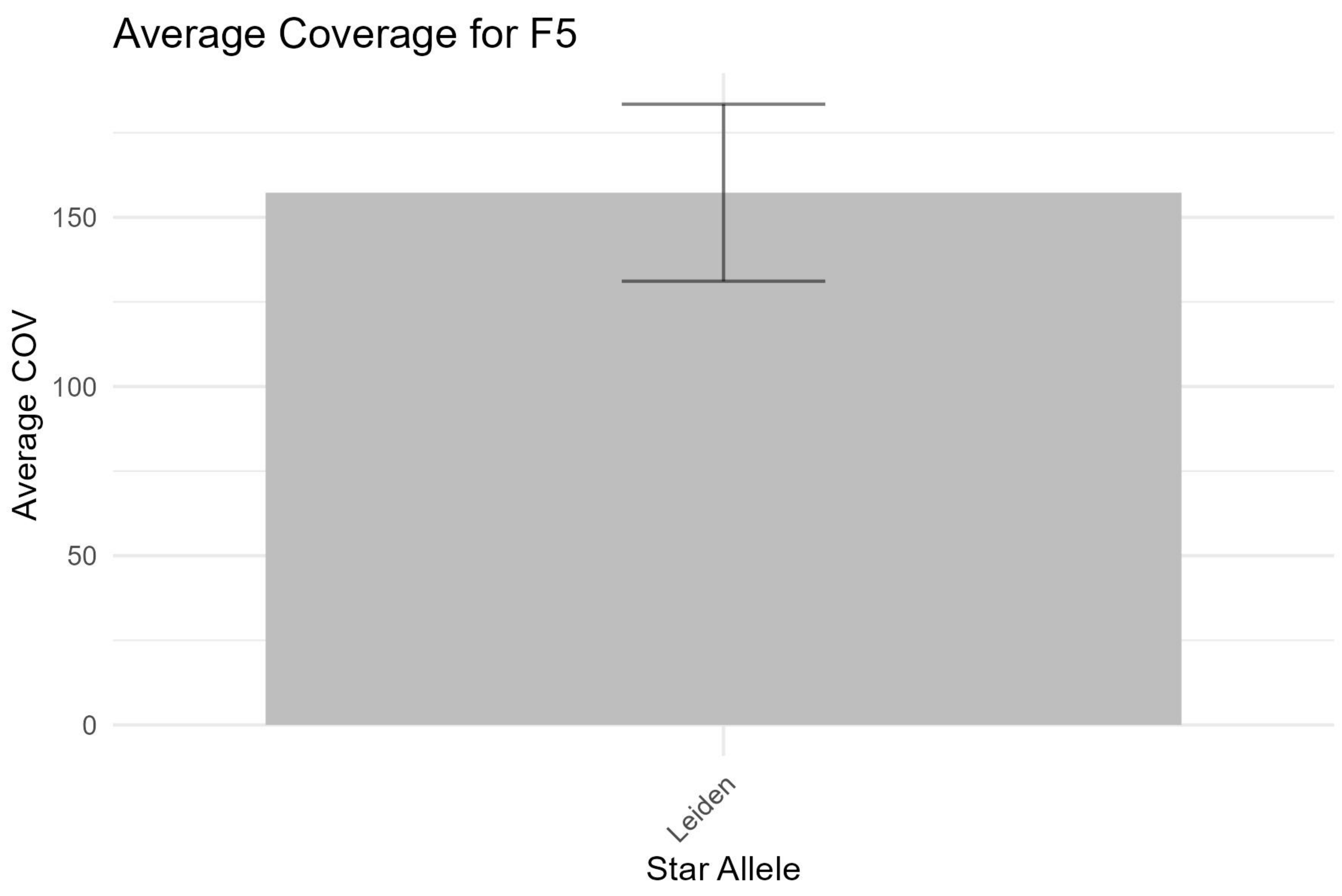
Average Coverage for F5
Average COV
150
100
50
0
Leiden
Star Allele

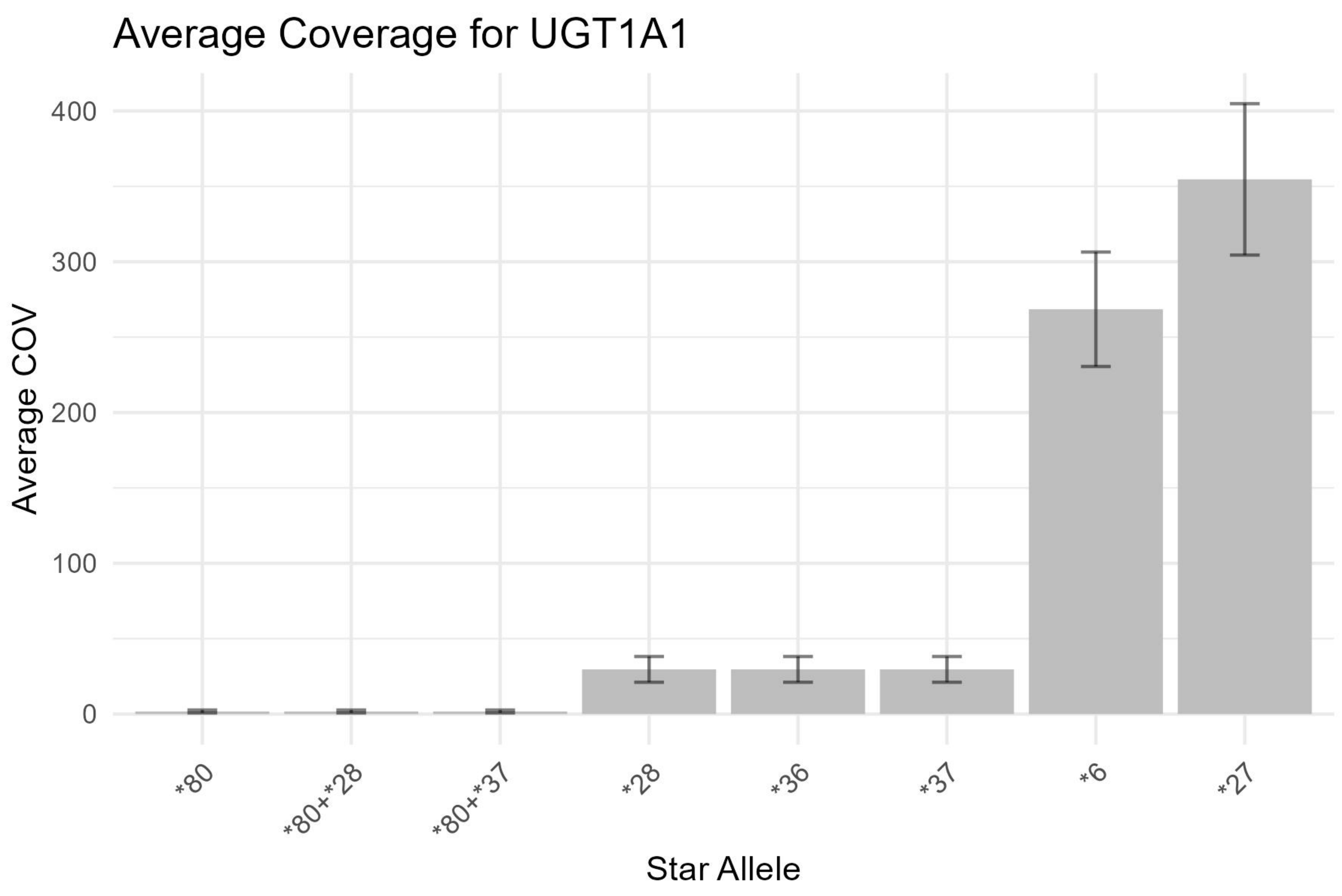
Average Coverage for UGT1A1
Average COV
400
300
200
100
0
*80
*80+*28
*80+*37
*28
*36
*37
*6
*27
Star Allele

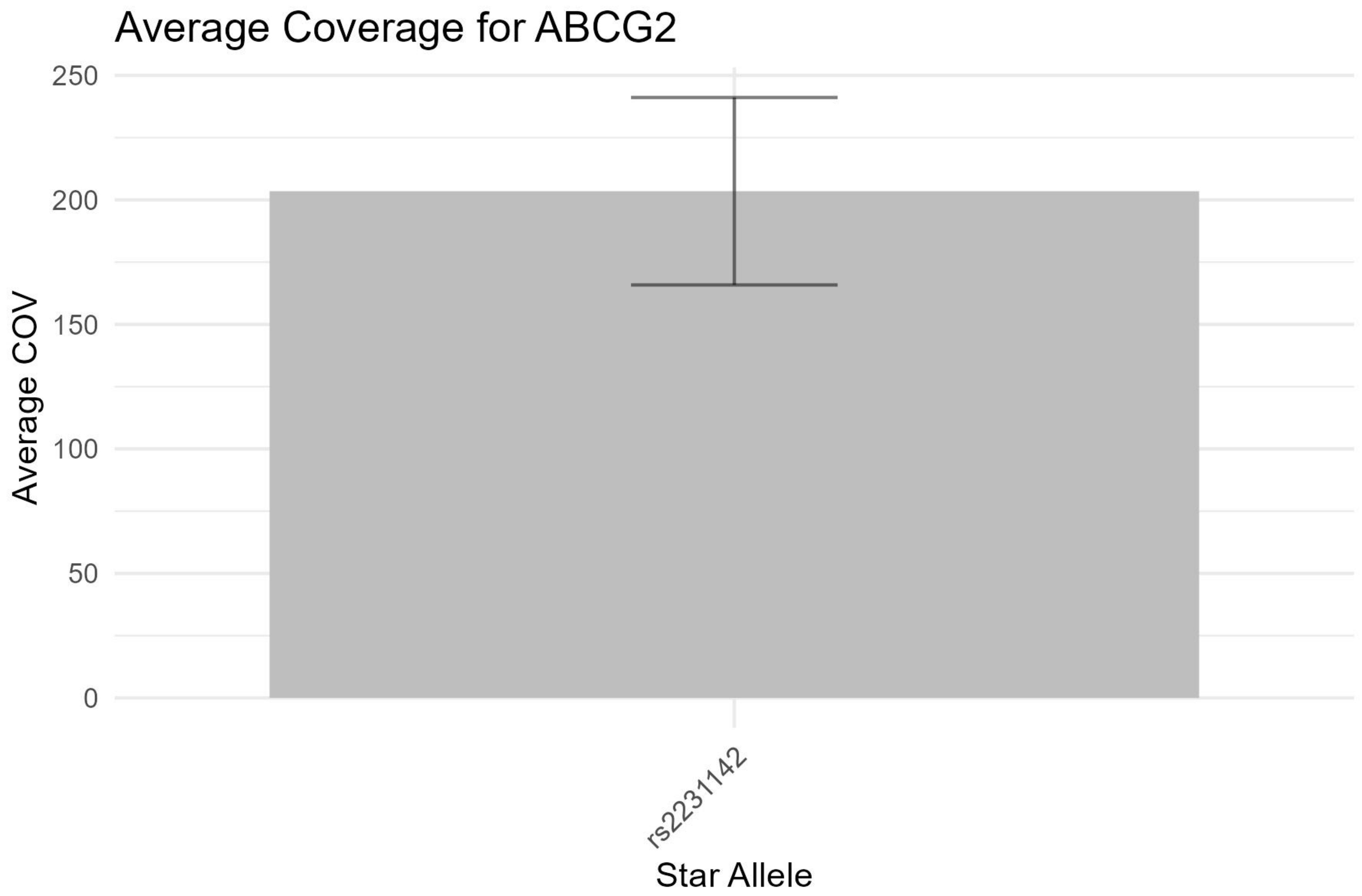

Average Coverage for ABCG2
250
200
150
100
50
0
Average COV
rs2231142
Star Allele

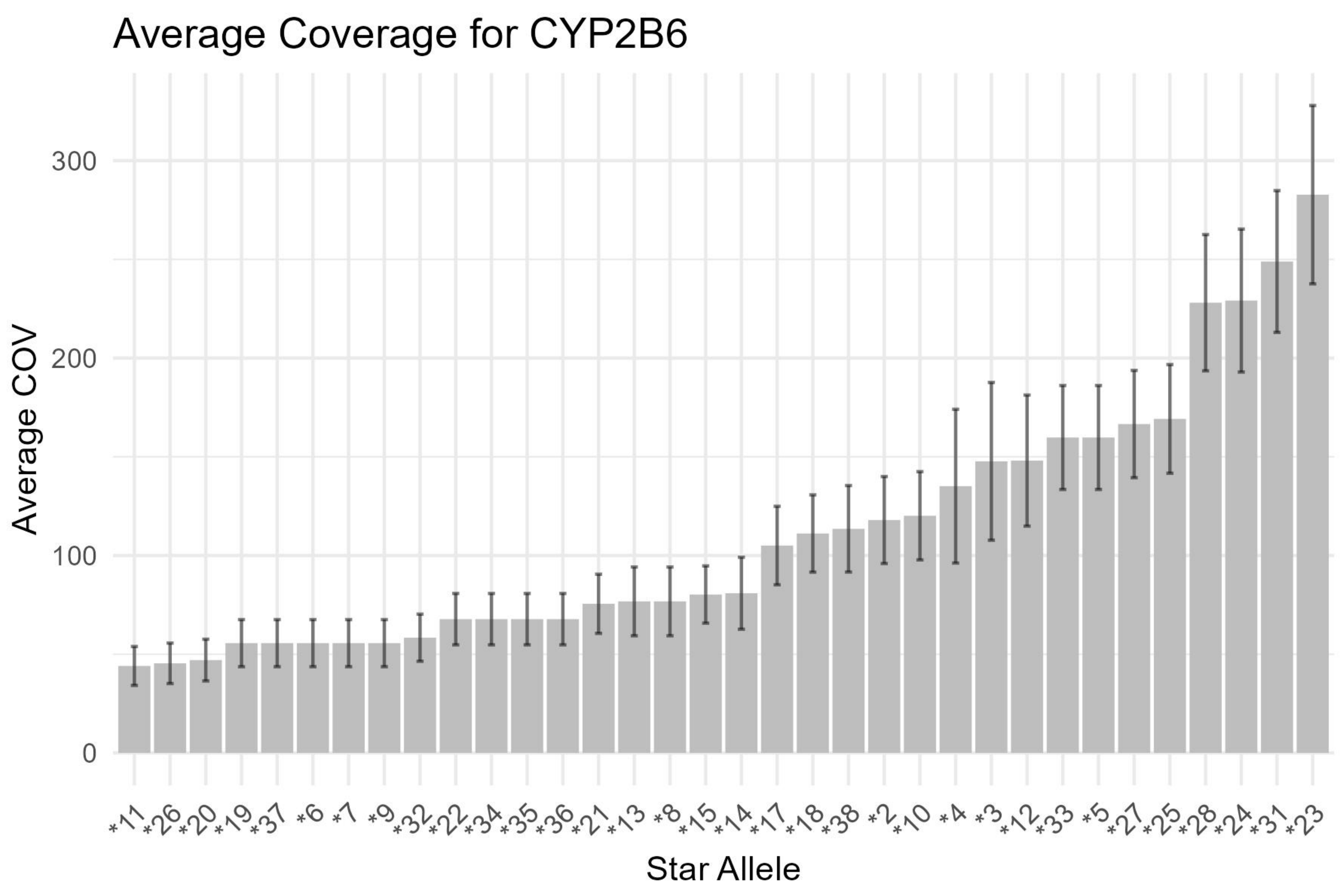
Average Coverage for CYP2B6
Average COV
0
100
200
300
*11
*26
*20
*19
*37
*6
*7
*9
*32
*22
*34
*35
*36
*21
*13
*8
*15
*14
*17
*18
*38
*2
*10
*4
*3
*12
*33
*5
*27
*25
*28
*24
*31
*23
Star Allele

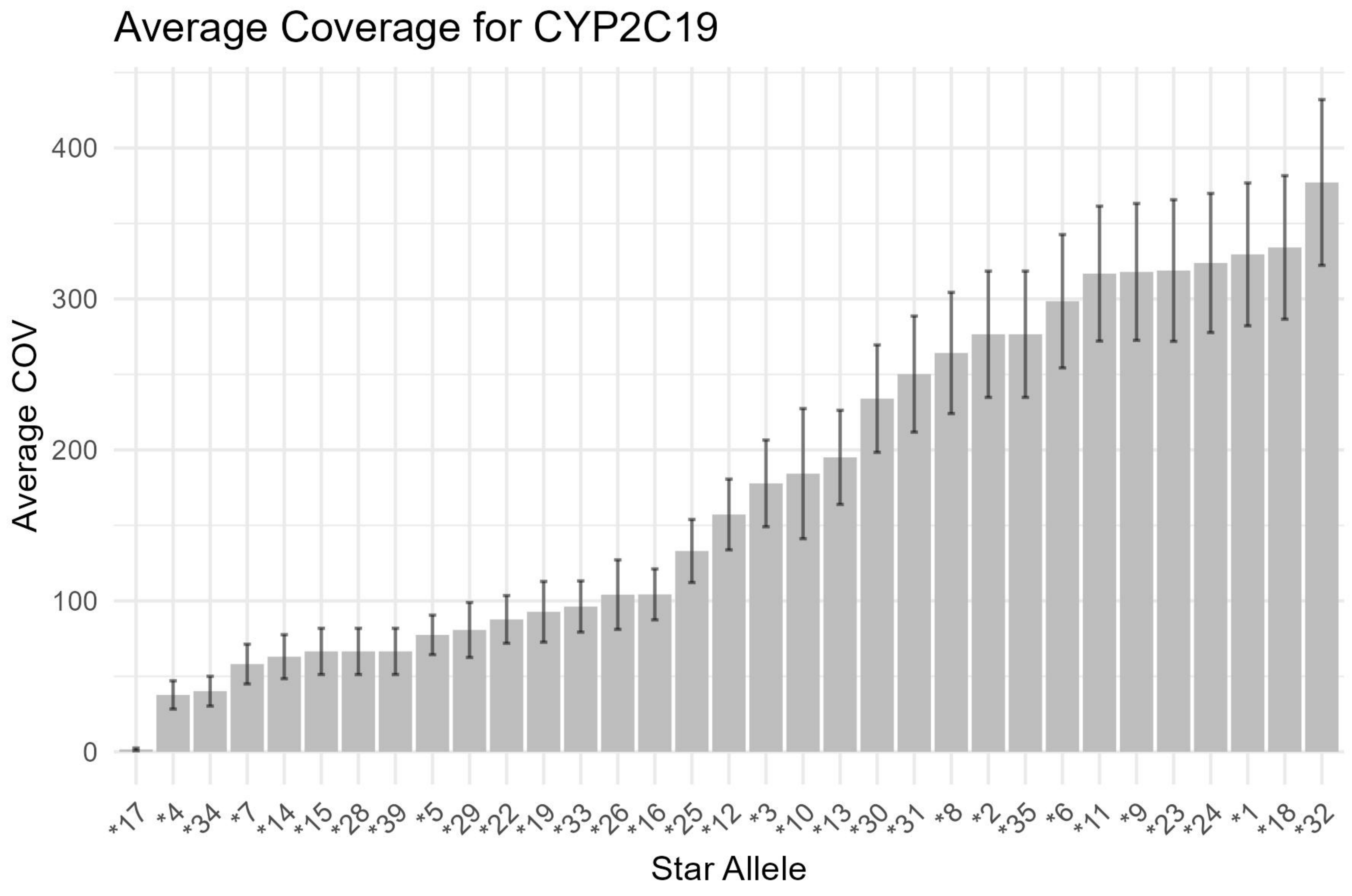
Average Coverage for CYP2C19
Average COV
400
300
200
100
0
*17
*4
*34
*7
*14
*15
*28
*39
*5
*29
*22
*19
*33
*26
*16
*25
*12
*3
*10
*13
*30
*31
*8
*2
*35
*6
*11
*9
*23
*24
*1
*18
*32
Star Allele

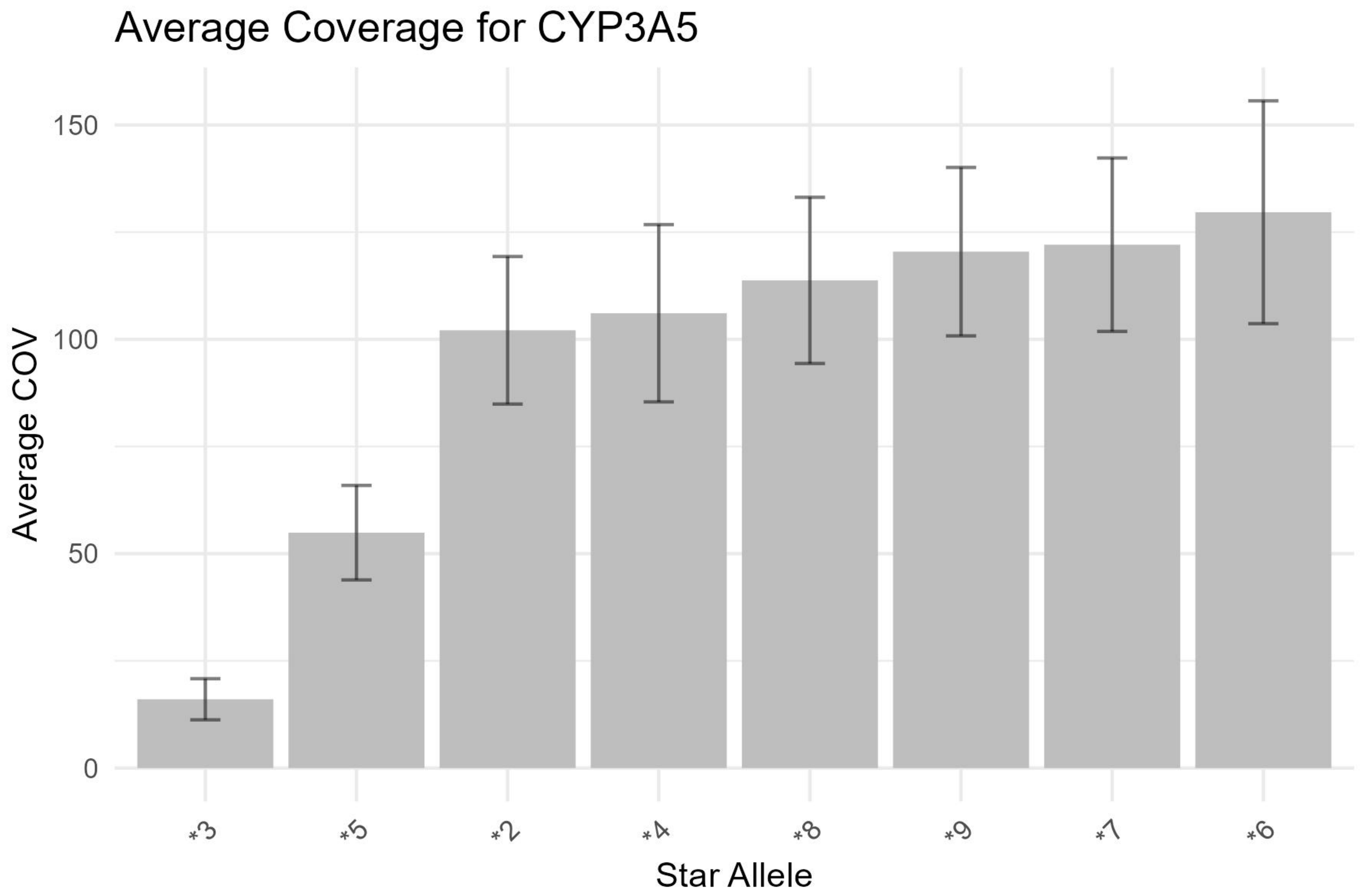
Average Coverage for CYP3A5
Average COV
0
50
100
150
*3
*5
*2
*4
*8
*9
*7
*6
Star Allele

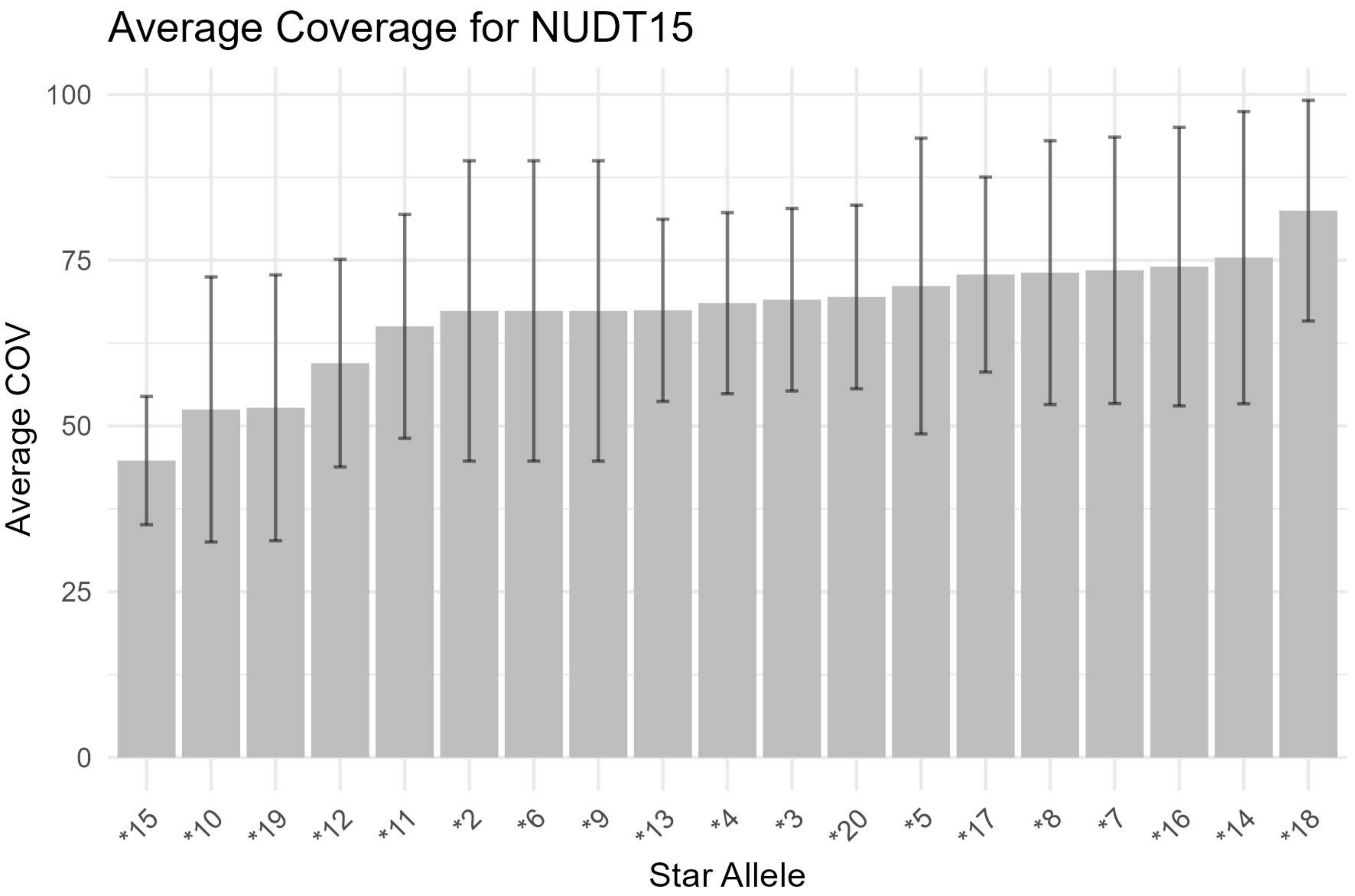
Average Coverage for NUDT15
Average COV
100
75
50
25
0
*15
*10
*19
*12
*11
*2
*6
*9
*13
*4
*3
*20
*5
*17
*8
*7
*16
*14
*18
Star Allele

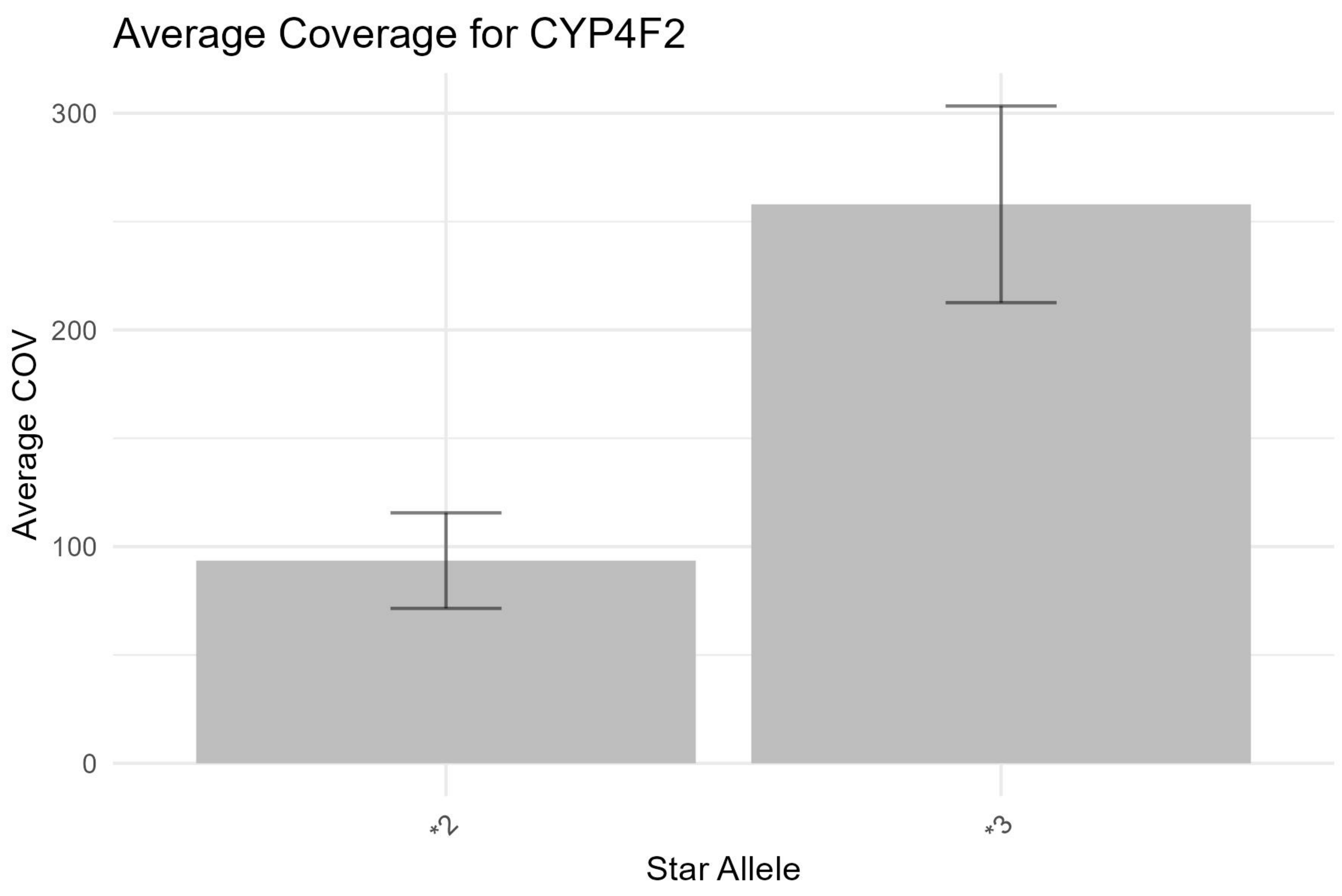
Average Coverage for CYP4F2
300
200
100
0
Average COV
*2
*3
Star Allele

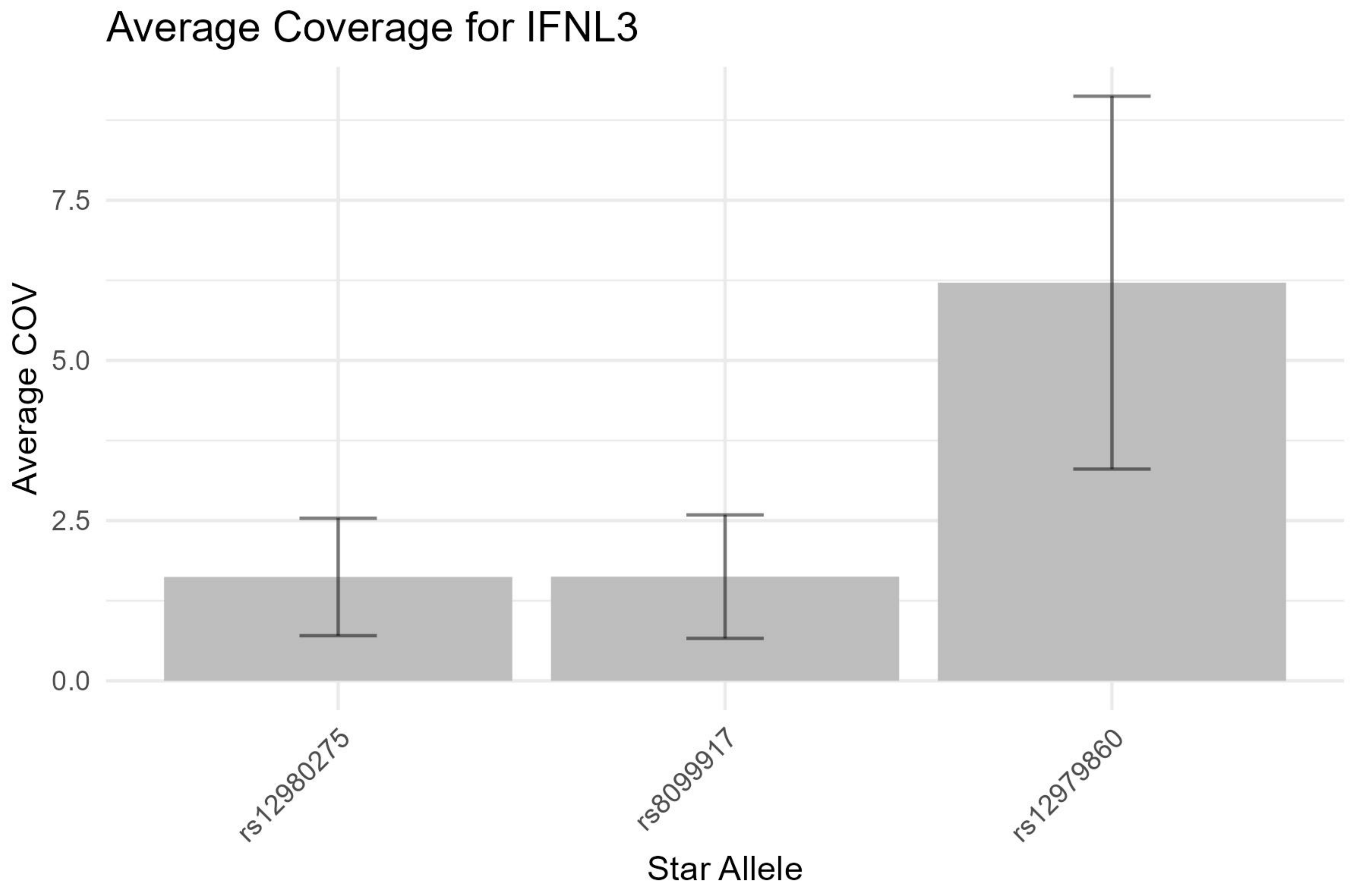
Average Coverage for IFNL3
Average COV
7.5
5.0
2.5
0.0
rs12980275
rs8099917
rs12979860
Star Allele

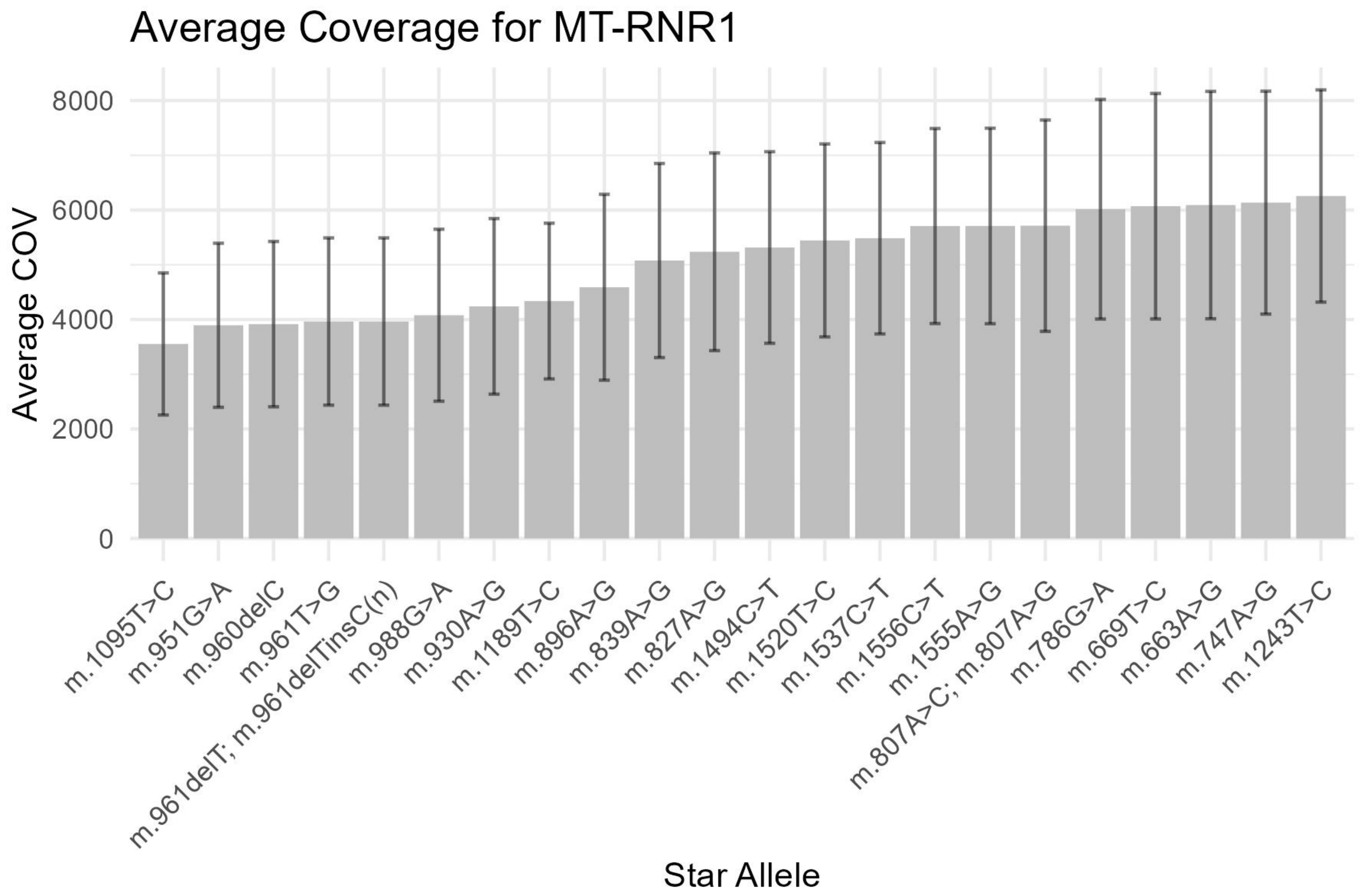

Average Coverage for MT-RNR1
Average COV
8000
6000
4000
2000
0
m.1095T>C
m.951G>A
m.960delC
m.961T>G
m.961delT; m.961delTinsC(n)
m.988G>A
m.930A>G
m.1189T>C
m.896A>G
m.839A>G
m.827A>G
m.1494C>T
m.1520T>C
m.1537C>T
m.1556C>T
m.1555A>G
m.807A>C; m.807A>G
m.786G>A
m.669T>C
m.663A>G
m.747A>G
m.1243T>C
Star Allele

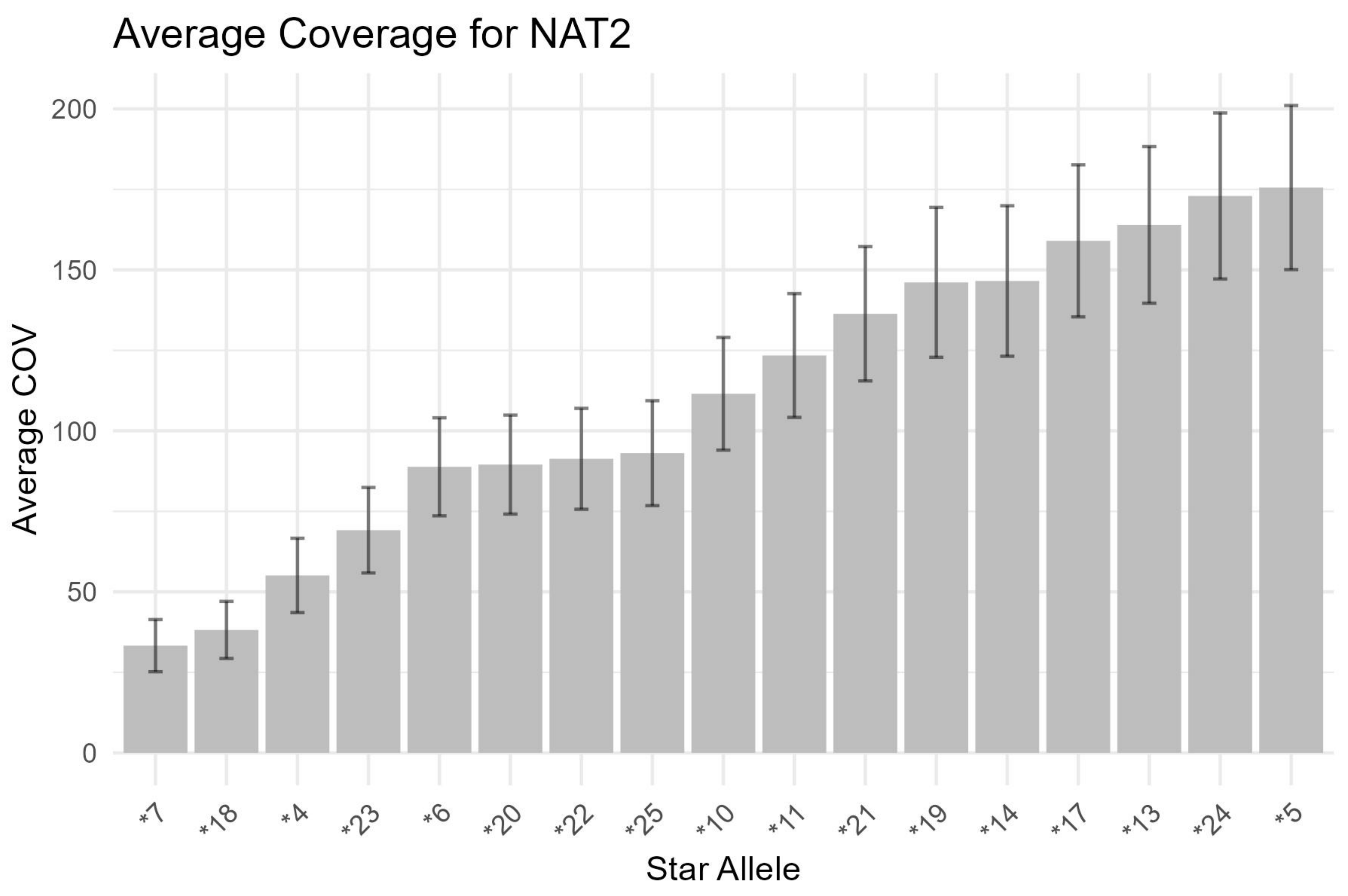

Average Coverage for NAT2
Average COV
200
150
100
50
0
*7
*18
*4
*23
*6
*20
*22
*25
*10
*11
*21
*19
*14
*17
*13
*24
*5
Star Allele

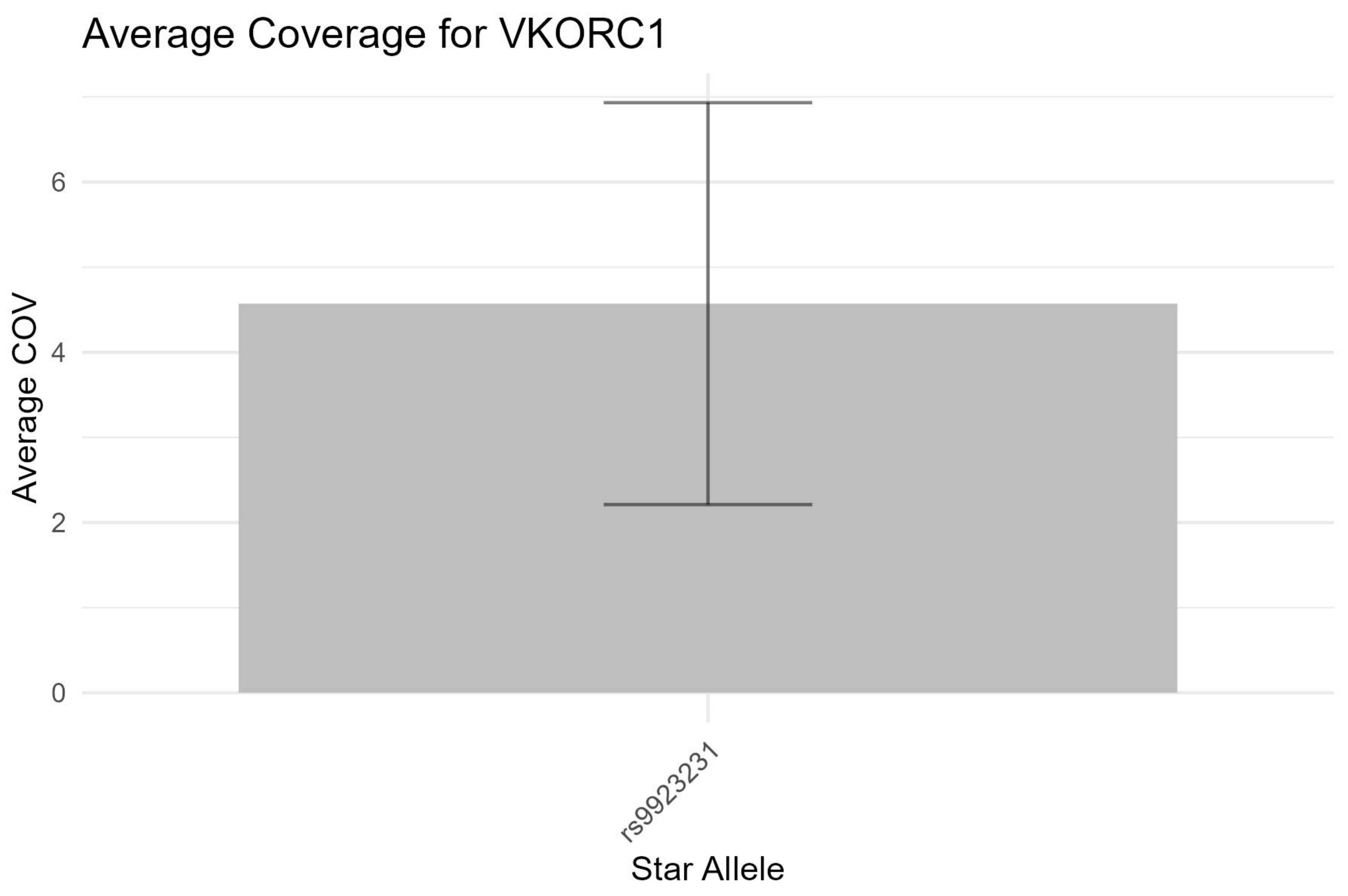
Average Coverage for VKORC1
Average COV
0
2
4
6
rs9923231
Star Allele
