# Supplementary material for: Clinical impact of pharmacogenomics in pediatric care: insights extracted from clinical exome sequencing
Source: Front Genet. 2025 May 29;16:1574325. doi: 10.3389/fgene.2025.1574325 (PMC12159002; doi:10.3389/fgene.2025.1574325)
Supplement: Supplementary file 11 [file DataSheet8.pdf]

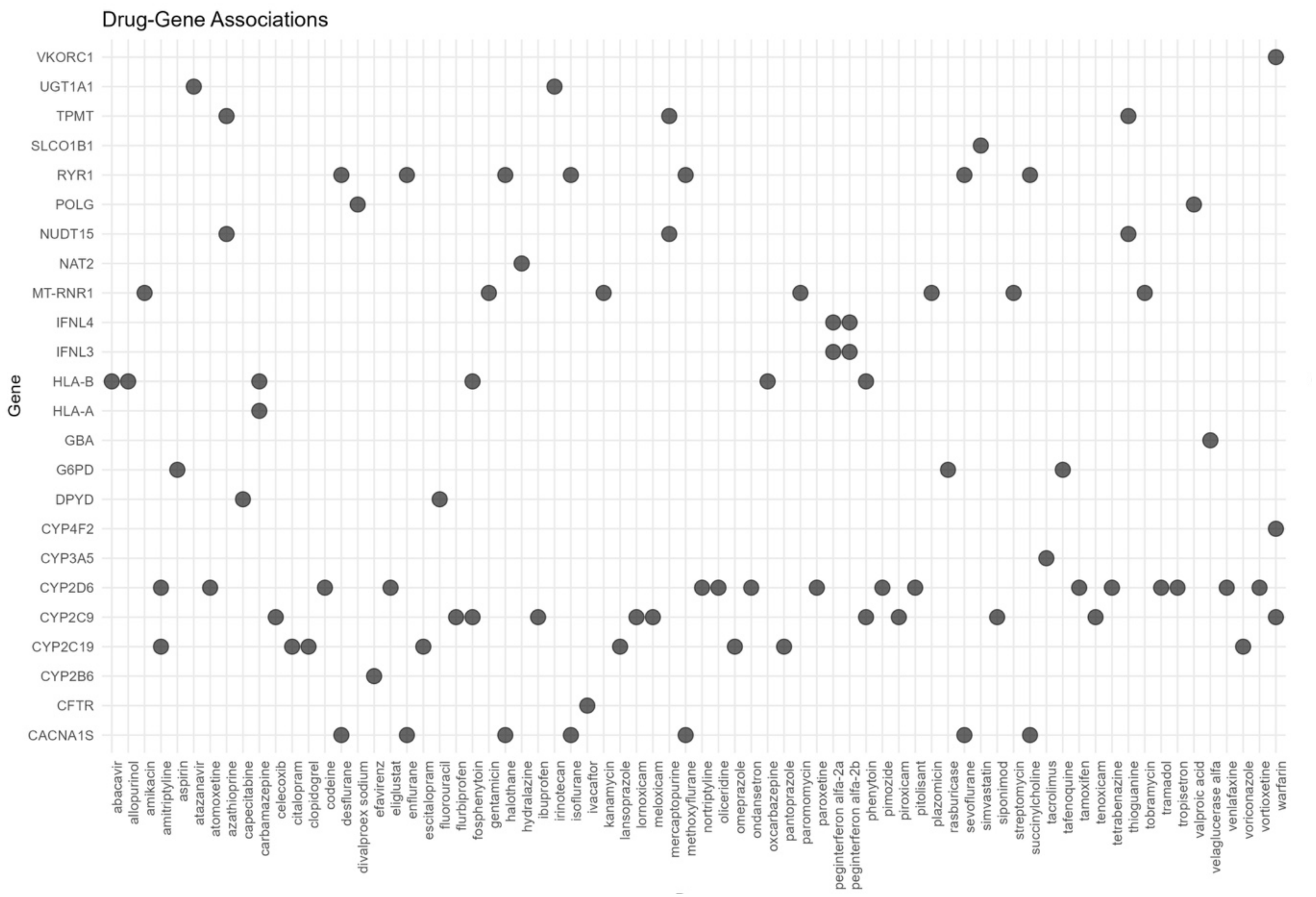

Data sheet 9. Illustrates drug-gene associations, majority of which are classified as CPIC level A/B and have existing prescribing guidelines.
